# Impact of the anti-inflammatory macrolide glasmacinal on the gut microbiota of healthy adults: an open-label trial

**DOI:** 10.1038/s41467-026-76867-9

**Published:** 2026-08-20

**Authors:** Tsegaye Sewunet, Mohammad Razavi, Kate Hanrott, Virginia Norris, Jennifer Kricker, Christian G. Giske

**Affiliations:** 1 Division of Clinical Microbiology, Department of Laboratory Medicine, Karolinska Institutet, Stockholm, Sweden; 2 EpiEndo Pharmaceuticals, Reykjavík, Iceland; 3 Department of Clinical Microbiology, Karolinska University Hospital, Stockholm, Sweden

**Keywords:** Outcomes research, Microbiome

## Abstract

Glasmacinal (EP395) is an oral macrolide, with immunomodulatory properties like antibiotic macrolides (such as azithromycin), but with negligible in vitro antimicrobial activity, which is being developed as a potential treatment to reduce exacerbations in respiratory conditions. In an open-label healthy participant trial (ClinicalTrials.gov: NCT06118684), with the primary objective of assessing potential drug-drug interactions of glasmacinal, faecal samples were collected to evaluate the impact on the gut microbiota of daily doses of glasmacinal for 2 weeks, using both phenotypic and 16S rRNA gene sequencing. Glasmacinal produced modest effects: reduced phylogenetic richness (p < 0.0001), but not evenness, and altered beta-diversity (PERMANOVA R^2^ = 0.104 p.adj=0.0018 after 1 week, and *R*^2^ = 0.059, p.adj=0.028 after 2 weeks) with reductions in *Clostridiaceae*, *Enterobacteriaceae*, and *Sutterellaceae* abundance. The community shift was approximately 10% after 1-week glasmacinal, and 6% after 2-weeks. With quantitative culture, only Enterobacterales was impacted. No increase or selection of resistance to azithromycin in Enterobacterales and *Bacteroides*, nor increase in *Candida spp*. or *Clostridioides difficile*, were detected. Overall, glasmacinal induced limited restructuring of the gut microbiome (without the defining hallmarks of antibiotic-like dysbiosis), core anaerobic phyla of the gut (Firmicutes and Bacteroidetes) were preserved, and there was no selection of antimicrobial resistance.

## Abstract (web summary)

Here, in a clinical trial in healthy participants, the authors show that glasmacinal, an oral macrolide with immunomodulatory properties, exerts limited changes to the gut microbiome and does select for antimicrobial resistance, unlike conventional antibiotics.

## Introduction

Macrolides, a class of broad-spectrum antibiotics of large molecular size, have been used for more than half a century to treat both local and systemic infections, including those of the skin, respiratory tract, gastrointestinal tract, and genital tract^1–3^, and include azithromycin, erythromycin, and clarithromycin. In addition to their antimicrobial effects, some macrolides, such as azithromycin, have immunomodulatory properties^4–6^, and when taken chronically reduce exacerbations of respiratory conditions, such as chronic obstructive pulmonary disease (COPD), bronchiectasis and asthma^7–11^. However, long-term use of antimicrobial macrolides is associated with the risk of increased selection of resistant fractions of microbiota or development of resistance against macrolides^12,13^. Whilst off-label azithromycin is included in the Global Initiative for Chronic Obstructive Lung Disease (GOLD) treatment guidelines as an option for patients who continue to exacerbate despite optimised standard of care, the benefit-risk of long-term use of antimicrobial macrolides in COPD patients has been questioned^14^. In addition to concerns about macrolide resistance, chronic antimicrobial use may lead to alterations in microbiome balance^15–17^. The balance of the microbiome in both the lung^18,19^ and gut^20^ shown to influence the occurrence and frequency of COPD symptoms. Dysbiosis (loss of the anaerobic taxa and increase in proteobacteria) has been shown to increase the risk of worsening COPD symptoms^21,22^. As such, chronic use of antibiotic macrolides may disrupt the gut microbiome with undesirable consequences, as well as increasing macrolide resistance. These findings led to the development of glasmacinal (EP395), an oral macrolide selected to retain the immunomodulatory properties of azithromycin, but lacking antimicrobial effects. Glasmacinal has the same backbone structure as azithromycin (including the large macrocyclic lactone ring), but with the cladinose moiety removed^23^. In both preclinical and clinical studies, glasmacinal has immunomodulatory effects^24–26^ but negligible in vitro antimicrobial activity (shown by minimal inhibitory concentration [MIC] testing against 117 isolates from 17 bacterial species)^23^. Glasmacinal is being developed as a potential treatment to reduce exacerbations in respiratory conditions, without the risk of developing antimicrobial resistance or induction of clinically meaningful gut dysbiosis.

The term ‘dysbiosis’ is frequently used to describe microbiome changes following an external perturbation, yet it encompasses a wide spectrum of phenomena that differ substantially in biological and clinical significance^27^. At an ecological level, restructuring of a microbial community, through transient changes in richness, relative abundance, or beta diversity, is an expected response to many stimuli, such as diet^28^, drugs^29,30^, inflammation^31^, or host physiology^32^, and does not always inherently imply pathology^33,34^. In contrast, clinically meaningful dysbiosis is typically characterised by persistent loss of key commensal anaerobic taxa, pronounced community dominance by pathobiont organisms, reduced ecological resilience, and increased rate of antimicrobial resistance or selection of resistant fractions of the gut microbe^33,35,36^. Importantly, these features tend to coincide and are sustained over time, reflecting a shift toward an unstable or pathogenic community state rather than adaptive reorganisation^33^. It is therefore important to distinguish between therapeutic microbiome modulation and clinically meaningful dysbiosis.

This paper reports an evaluation of the impact of glasmacinal on the gut microbiome, assessing the potential for glasmacinal to induce antibiotic-like gut dysbiosis and selection of resistance. The study aimed to evaluate loss of beneficial anaerobic taxa (Firmicutes and Bacteroidetes), expansion of pathobionts (such as Proteobacteria) and taxa typically associated with antimicrobial selection, and significant loss of microbial diversity following administration of glasmacinal. The effect of daily glasmacinal for 2 weeks on the gut microbiota of healthy adults was assessed as an exploratory endpoint in a clinical pharmacology trial^37^, using both phenotypic and 16S rRNA gene sequencing. The trial shows that glasmacinal induces limited restructuring of the gut microbiome, without the defining hallmarks of antibiotic-like dysbiosis.

## Results

### Summary of trial population and sample collection

**Fig. 1:**
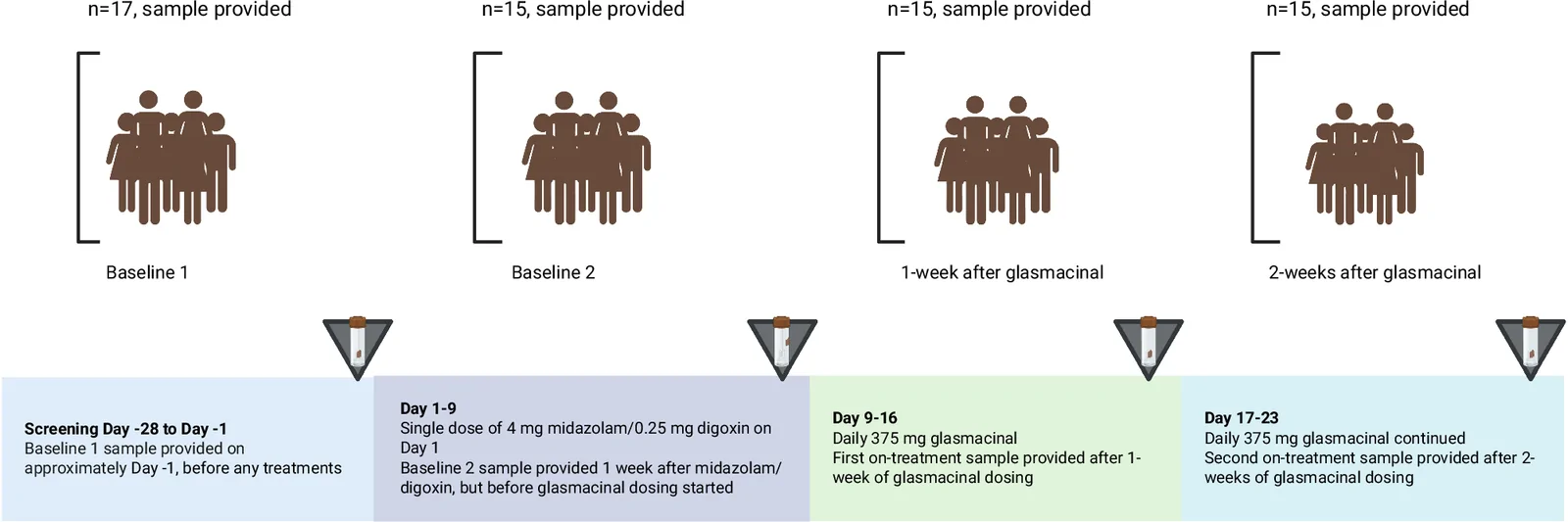
Trial design and faecal sample collection time-points. The primary objective of the trial was to evaluate potential drug-drug interactions of glasmacinal (refer to separate publication^37^). An exploratory objective was to assess the effect of daily doses of glasmacinal (375 mg) for 2 weeks on the gut microbiome. Participants were screened, and eligible participants provided a sample on Day -1, before any trial treatments were administered (Baseline 1; *n* = 17). Participants received a single dose of 4 mg midazolam and 0.25 mg digoxin on Day 1. One week later, they provided a faecal sample (Baseline 2; *n* = 15). Glasmacinal (375 mg) was administered daily from Days 9–28, after at least 10 h fasting. One week after starting glasmacinal, the first on-treatment sample was collected (1-week glasmacinal; *n* = 15). Two weeks after starting glasmacinal, the second on-treatment sample was collected (2-weeks glasmacinal; *n* = 15). Created in BioRender. Sewunet, T. (2026) https://BioRender.com/cc39ccy.

The faecal samples analysed in this study were collected from a clinical pharmacology trial with the primary objective of assessing potential drug-drug interactions of glasmacinal^37^. The details of the trial design and the sample collection time-points are presented in the methods section and summarised in Fig. 1. Briefly, two baseline faecal samples were collected (Baseline 1, before any trial treatments; and Baseline 2, 1-week after a single dose of midazolam and digoxin), and two on-treatment faecal samples (after 1-week of glasmacinal and 2-weeks of glasmacinal). Nineteen participants were enroled in the trial, and 17 participants were dosed with glasmacinal, and provided faecal samples. Participant demographic characteristics and disposition details are summarised in Table 1. The number of samples available at each time-point is detailed in the Methods section and Fig. 1. None of the participants had any significant medical history, and the only concomitant medications taken during the trial were paracetamol, ibuprofen, and hormonal contraceptives.

**Table 1:** Demographic characteristics of participants in the trial

Participants enroled in the trial (*N*)	19
Participants dosed with at least one dose of glasmacinal and included in the microbiome exploratory analysis (*N*)	17
Participants completed (*N*)	14^a^
Female, *n* (%)	11 (58)
Male, *n* (%)	8 (42)
Age, years, median (min, max)	34.0 (19–52)
Body mass index, kg/m^2^, mean (SD)	25.19 (2.82)
Race, White, *n* (%)	17 (89)
Race, Asian, *n* (%)	2 (11)
Ethnicity, Not Hispanic or Latino, *n* (%)	17 (89)
Ethnicity, Hispanic or Latino, *n* (%)	1 (5.3)
Ethnicity, Unknown, *n* (%)	1 (5.3)

^a^5 participants did not complete the trial: 2 withdrew consent, 1 was withdrawn by the investigator because of anxiety, 1 was withdrawn because the participant could not comply with the trial schedule, and 1 completed all visits, but missed the end of trial telephone follow-up (this participant provided faecal samples at all time-points).

### Diversity metrics

**Fig. 2:**
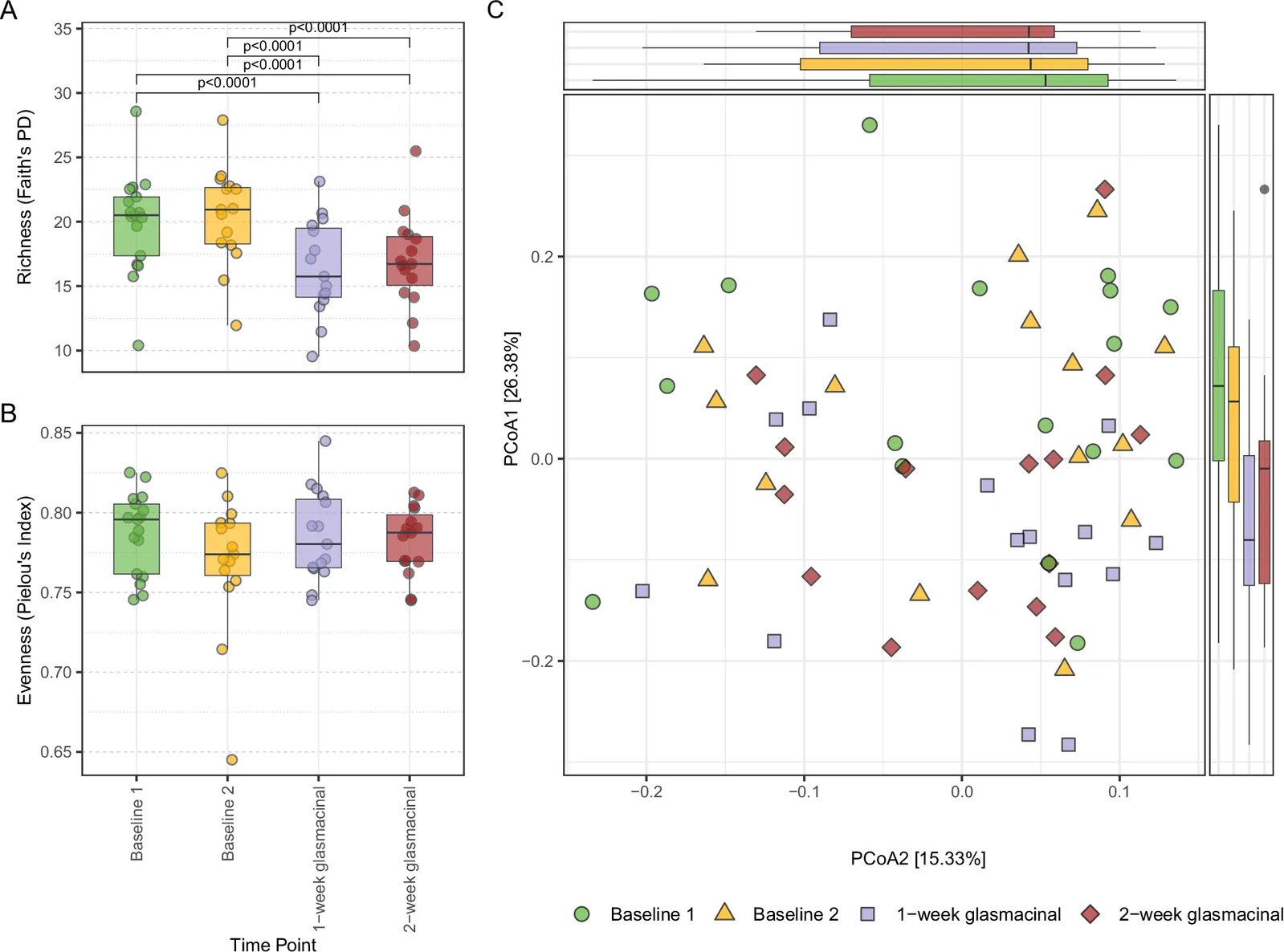
The effect of glasmacinal treatment on microbiome diversity and composition. Two baseline faecal samples were collected (Baseline 1 [*n* = 17] and Baseline 2 [*n* = 15]), together with samples collected after 1 week (*n* = 15) and 2 weeks (*n* = 15) of glasmacinal treatment. **A** Faith’s phylogenetic diversity (PD) and **B** Pielou’s evenness. Differences between visits were assessed using two-sided linear mixed-effects models with participant as a random intercept, followed by two-sided estimated-marginal-means comparisons with Benjamini–Hochberg false-discovery-rate adjustment. Faith’s PD differed across visits, *F* (3, 42.19) = 31.36, *P* = 8.01 × 10^−11^, and was lower after 1 week than at Baseline 1 (estimated difference = 3.44, 95% CI 2.48–4.41; *t* (42.08) = 7.20, adjusted *P* = 2.27 × 10^−8^) and Baseline 2 (3.63, 95% CI 2.63–4.64; *t* (42.18) = 7.28, adjusted *P* = 2.27 × 10^−8^), and after 2 weeks than at Baseline 1 (3.05, 95% CI 2.08–4.02; *t* (42.08) = 6.37, adjusted *P* = 1.71 × 10^−7^) and Baseline 2 (3.24, 95% CI 2.23–4.25; *t* (42.18) = 6.49, adjusted *P* = 1.54 × 10^−7^). Pielou’s evenness did not differ across visits, *F* (3, 43.10) = 1.11, *P* = 0.355. **C** Principal coordinates analysis based on weighted UniFrac distances. Samples are distinguished by colour and marker shape, with marginal box plots summarising their distributions along each axis. In all box plots, centre lines indicate medians, boxes indicate the 25th–75th percentiles, and whiskers extend to the most extreme values within 1.5 times the interquartile range.

Alpha diversity, as measured by Faith’s phylogenetic diversity (PD), showed transient reductions following glasmacinal administration. Both Baseline 1 and Baseline 2 were significantly higher than after 1-week and 2-weeks glasmacinal treatment, with the greatest change observed at 1-week and a partial recovery by 2-weeks (Fig. 2A). Faith’s PD reflects the total branch length of the phylogenetic tree spanned by the observed taxa, so this decrease indicates that some phylogenetically unique or distantly related lineages were selectively depleted. Pielou’s evenness (alpha diversity), however, remained largely unchanged (Fig. 2B), meaning that the relative abundance distribution among the taxa that persisted in the community did not undergo major reordering; no strong dominance by a few taxa emerged, and no large-scale expansion of opportunists occurred.

Beta diversity, represented by the weighted UniFrac metric, revealed modest but significant restructuring of the gut microbiome following glasmacinal administration (Table 2 and Fig. 2C). The largest changes were observed after 1-week glasmacinal (Baseline 1 versus 1-week R² = 0.104, p.adj = 0.0018; Baseline 2 versus 1-week *R*² = 0.067, p.adj = 0.0048). Differences after 2-weeks glasmacinal were smaller (Baseline 1 versus 2-weeks *R*² = 0.059, p.adj = 0.028; Baseline 2 versus 2-weeks not significant). These results indicate that glasmacinal induced limited, transient shifts across phylogenetically related taxa, without causing sustained community-level disruptions. Participant level alpha and beta diversity values are given in Supplementary Data 2 and Supplementary Data 3, respectively.

**Table 2:** Pairwise PERMANOVA results for weighted UniFrac distances across visits

Comparison	F.stat	*R*^2^	*P*-value	P.adj	Significance
Baseline-1−1-week glasmacinal	3.4971	0.1044	0.0003	0.0018	**
Baseline 1−2-weeks glasmacinal	1.8743	0.0588	0.014	0.028	*
Baseline-1 – Baseline 2	0.3013	0.0099	0.7977	0.7977	
Baseline 2 – 1-week glasmacinal	2.0093	0.067	0.0016	0.0048	**
Baseline 2–2-weeks glasmacinal	1.1889	0.0407	0.0642	0.0963	
1-week glasmacinal – 2-weeks glasmacinal	0.6188	0.0216	0.2271	0.2725	

Comparison between pairs of time-points (Baseline 1 [*n* = 17], Baseline 2 [*n* = 15], 1-week glasmacinal [*n* = 15], and 2-weeks glasmacinal [*n* = 15]). Pairwise non-directional PERMANOVA was conducted using 9999 permutations stratified by participant. F.stat denotes the pseudo-F statistic, *R*² the proportion of variance explained by visit, *P*-value the nominal permutation-derived *P* value, and P.adj the Benjamini–Hochberg false-discovery-rate-adjusted *P* value. Significance is indicated as **P.adj ≤ 0.01, *P.adj ≤ 0.05, blank entries indicate non-significant comparisons.

### Microbial community composition: differential abundance analysis

Differential abundance was first assessed using ANCOM-BC2^38^ on the unrarefied raw feature-count table (Supplementary Data 4), with time points included as a fixed effect and participant as a random intercept to account for repeated measurements. At ASV level, ANCOM-BC2 identified only one differentially abundant ASV (Supplementary Data 5), despite evidence of treatment-associated community change from the alpha-diversity, beta-diversity, and culture-based analyses. This ASV was classified as *Christensenellaceae R-7 group* (family *Christensenellaceae*) and decreased in abundance at 1-week (log2FC = −4.93, p.adj = 0.031).

This discrepancy likely reflects the modest cohort size, high inter-individual variability, and the sparsity of ASV-level count data, combined with the burden of testing more than a thousand features. ANCOM-BC2’s bias-correction and pseudo-count sensitivity procedures are designed to protect against false-positive associations under these conditions, but this comes at the cost of reduced sensitivity when applied at fine taxonomic resolution in a small, sparse dataset. Because the restricted ASV-level result provided limited resolution of the taxonomic response in this longitudinal cohort, LinDA^39^ was subsequently used to characterise the broader pattern of differential abundance while retaining the same mixed-effects structure. Differential abundance analysis using LinDA identified a broad but sparse set of amplicon sequence variant (ASVs) exhibiting significant changes relative to Baseline 1 (Supplementary Data 6). After prevalence filtering, 1180 ASVs were tested, of which 76 were differentially abundant at 1-week and 59 at 2-weeks (adjusted *p* < 0.05 and |log2FC | > 1; Supplementary Data 6). Differentially abundant ASVs included both increases and decreases in abundance and were observed across multiple taxonomic families.

The difference between methods was also evident after taxonomic aggregation. Across all separately analysed taxonomic ranks, LinDA identified 291 significant taxon-by-timepoint contrasts, comprising 135 at ASV, 75 at genus, 39 at family, 25 at order, 10 at class, and 7 at phylum level. ANCOM-BC2 identified 22 robust contrasts: 1 at ASV, 12 at genus, 1 at family, 4 at order, 2 at class, and 2 at phylum level. Because findings at adjacent taxonomic ranks are hierarchically related, these totals do not represent independent biological events. Apart from two genus-level associations, every contrast identified by ANCOM-BC2 was also identified by LinDA, consistent with ANCOM-BC2 operating as a more conservative subset of the LinDA-identified response rather than an independent or contradictory one. Critically, the direction of effect was concordant for all shared associations without exception (Supplementary Data 7). ANCOM-BC2 uniquely identified increases in *Dorea* and *Parabacteroides* genera at 1-week; both passed pseudo-count sensitivity testing but were not significant at 2-weeks.

**Fig. 3:**
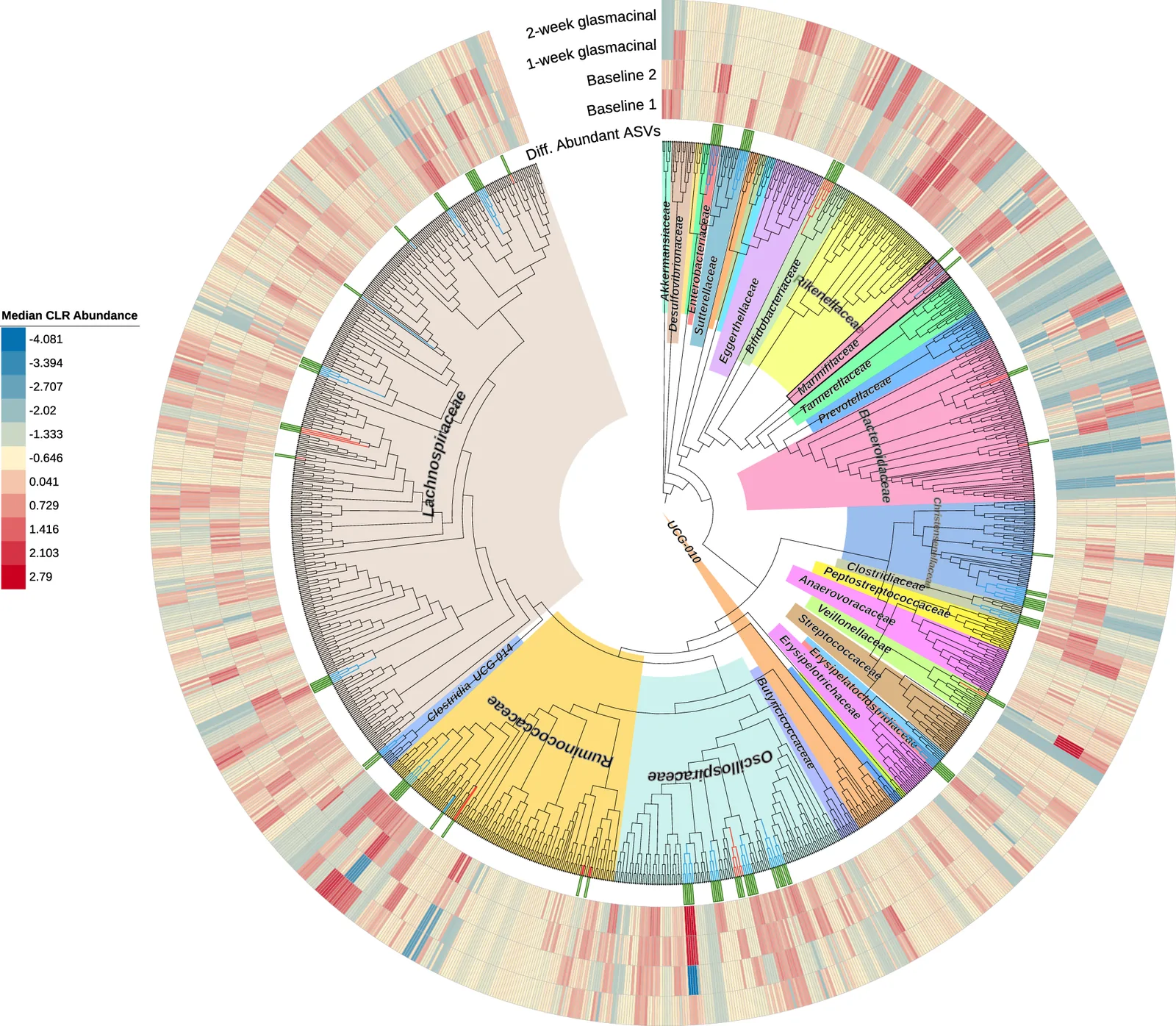
Phylogenetic context and temporal patterns of studied amplicon sequence variants (ASVs). Phylogenetic tree of ASVs included in the differential abundance analysis (prevalence ≥30% in baseline or on-treatment samples), with branches coloured by taxonomic family. Heatmap columns show median centred log-ratio (CLR)-transformed abundances across time-points, illustrating temporal abundance patterns for each ASV. The additional annotation indicates ASVs identified as differentially abundant at 1-week glasmacinal (76 ASVs) or 2-weeks glasmacinal (59 ASVs) relative to Baseline 1 (linDA; adjusted *p* < 0.05, |log2 fold change | > 1). Phylogenetic clustering/dispersion of all FDR-significant ASVs was assessed using a two-sided permutation test of mean pairwise phylogenetic distance with 9999 permutations. No significant departure from the background phylogenetic distribution was detected (standardised effect size = −1.66, *P* = 0.053).

Visualisation of linDA differentially abundant ASVs on a phylogenetic tree (Fig. 3 and Supplementary Data 6) revealed that these changes were distributed across the tree rather than concentrated within a single lineage or taxonomic clade. This was supported by permutation-based phylogenetic dispersion analysis using all FDR-significant ASVs as the foreground set, which showed no significant phylogenetic clustering relative to the tested ASV background after 9999 permutations (*p* = 0.053, standardised effect size = −1.66). No individual family accounted for a dominant proportion of the changes detected. Heatmap representation of median centred log-ratio (CLR) abundances across visits further illustrated heterogeneous temporal patterns among affected ASVs, with some changes evident at 1 week only, while others persisted to the 2-week time-point.

**Fig. 4:**
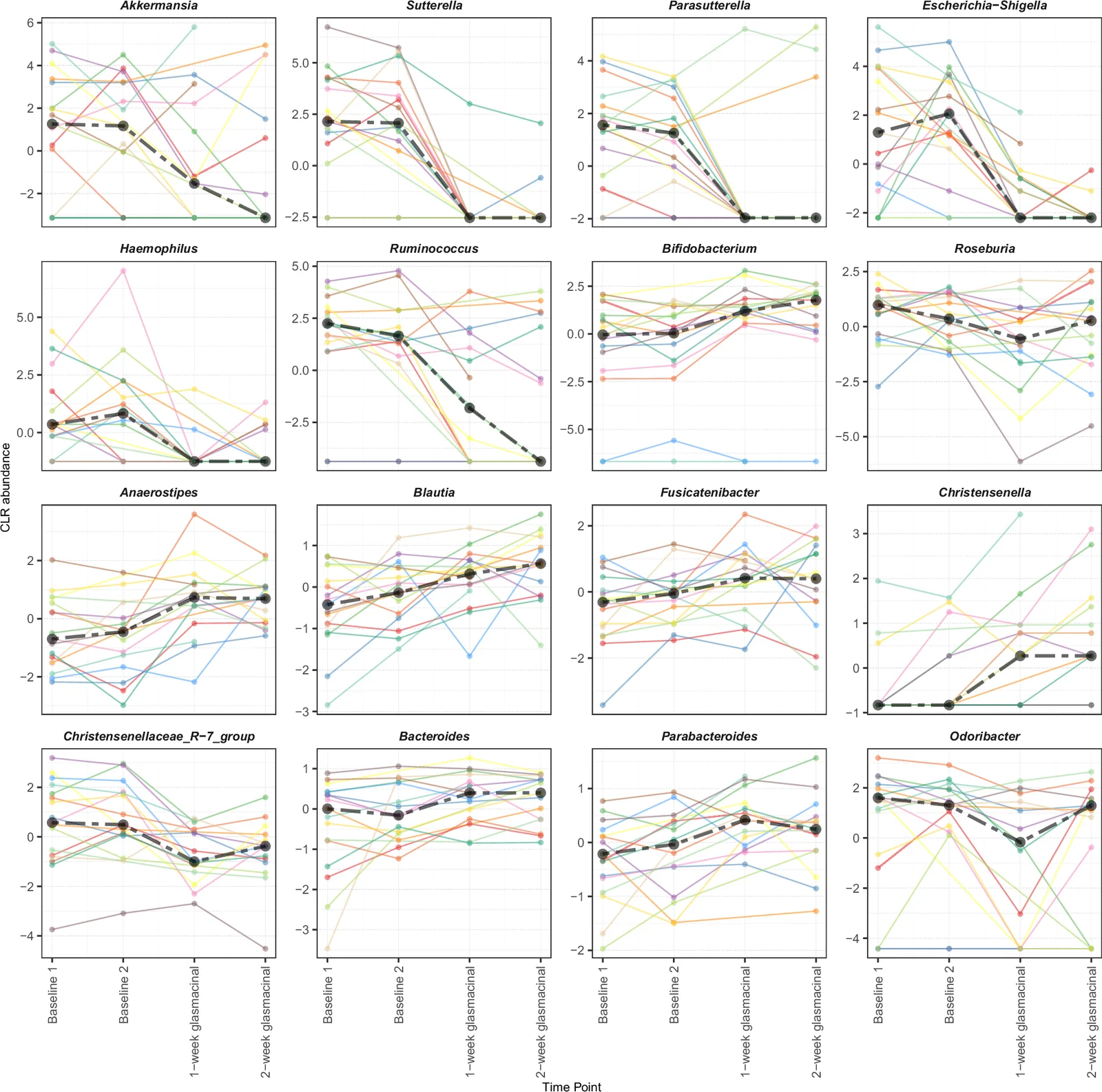
Longitudinal centred log-ratio (CLR) abundance of bacterial genera. Two baseline faecal samples were collected (Baseline 1 [*n* = 17] and Baseline 2 [*n* = 15]), and two on-treatment samples (after 1-week [*n* = 15] and 2-weeks [*n* = 15] of glasmacinal). Individual participant trajectories (coloured lines) are overlaid with population-level median trends (black line).

To contextualise the distributed differential abundance patterns observed across the phylogenetic tree, longitudinal trajectories of selected genera with well-established roles in gut microbial ecology were examined (Fig. 4). When visualised as CLR-transformed abundances across visits, several genera exhibited coherent, genus-specific temporal responses rather than uniform shifts across the community. Genera that were relatively abundant at baseline, including *Akkermansia*, *Ruminococcus*, *Sutterella*, *Parasutterella*, *Escherichia/Shigella*, and *Haemophilus*, showed pronounced reductions at 1-week, with low median abundances persisting or further declining at 2-weeks. In contrast, multiple commensal genera, such as Bifidobacterium, *Blautia*, *Anaerostipes*, *Fusicatenibacter*, *Parabacteroides*, and *Christensenella*, displayed increased median abundance following treatment, with gains evident at 1-week and generally maintained at 2-weeks. A subset of taxa, including *Odoribacter* and *Roseburia*, showed partial recovery or non-monotonic trajectories, suggesting heterogeneous temporal responses within functionally related groups.

### Sensitivity analysis of 16S gene copy-number correction

To evaluate whether 16S gene-copy number (GCN) variation influenced the findings, the complete microbiome analysis was repeated after 16S GCN correction. Correction reduced effective feature-table abundance, as expected, while preserving nearly all ASVs (7626/7644; 99.8%). Alpha-diversity conclusions were unchanged for Faith’s phylogenetic diversity and Pielou’s evenness. Weighted UniFrac beta-diversity structure was also strongly preserved between branches (Procrustes *M*² = 0.1626, *r* = 0.9151, Protest *p* = 0.0001). Pairwise PERMANOVA results were largely consistent, with only the borderline baseline 2–2-week glasmacinal contrast changing significance after correction despite similar R² values. Differential-abundance effect sizes remained strongly correlated across taxonomic levels; although more ASVs were significant after correction, higher-rank taxa were generally similar or fewer. Overall, copy-number correction did not materially alter the primary conclusions and supports the interpretation of limited ecological restructuring rather than broad antibiotic-like dysbiosis. The full comparison is provided in Supplementary information, section 1 and Supplementary Data 10–13.

### Sensitivity of the paired design to antibiotic-like dysbiosis

Moreover, to place the microbiome findings in context and assess whether the paired longitudinal framework was sensitive to large antibiotic-like perturbations, we performed a post-hoc sensitivity analysis (Supplementary information, section 2). This analysis used the glasmacinal dataset itself as an internal calibration of sensitivity and used external antibiotic datasets as benchmarks for the magnitude and pattern of effects expected during antibiotic-associated dysbiosis. The negligible baseline-to-baseline drift showed that the paired design was not dominated by background temporal variation, while the detection of large on-treatment reductions in Faith’s PD showed that the available sample size was sufficient to detect alpha-diversity changes of this magnitude when they occur consistently across participants. Similarly, the detection of a small but significant beta-diversity shift after glasmacinal indicated that the beta-diversity analysis was sensitive even to modest community displacement. The sensitivity analysis does not imply that the study was powered for all microbiome outcomes, but it supports that the cohort and paired analytical framework were adequate to detect large, coordinated antibiotic-like perturbations had they occurred.

### Quantitative culture and selection of resistance

**Fig. 5:**
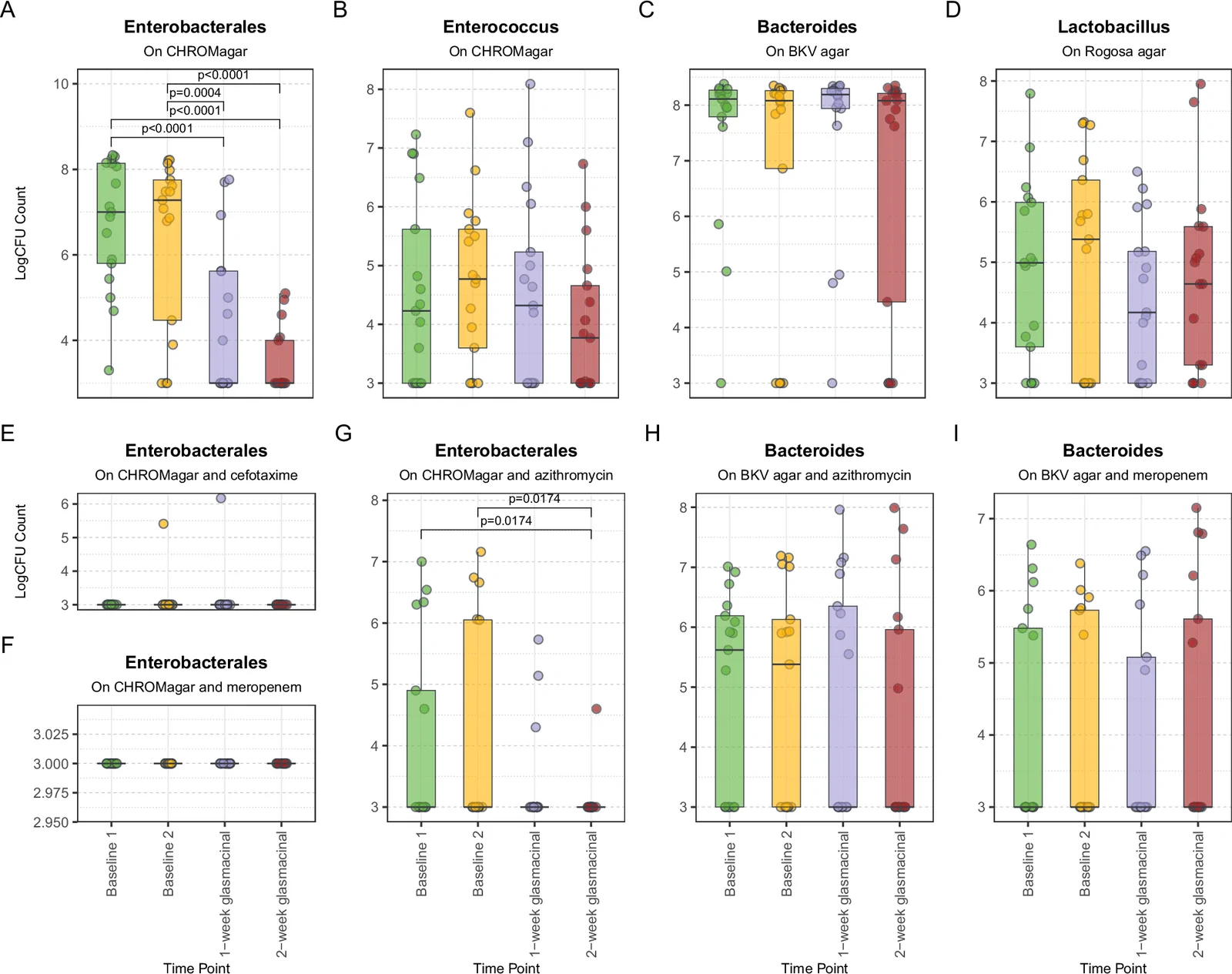
Quantitative culture and selection of resistance. Clinically relevant taxa were cultivated, measured using quantified by culture. Colony counts were corrected for dilution and plated volume, log-transformed as logCFU, and analysed using linear mixed-effects models with visit as a fixed effect and participant as a random intercept. Overall visit effects were assessed using F tests, followed by two-sided estimated-marginal-means t tests with Benjamini–Hochberg false-discovery-rate adjustment for pairwise comparisons. Two baseline faecal samples were collected (Baseline 1 [*n* = 17] and Baseline 2 [*n* = 15]), together with samples collected after 1 week (*n* = 15) and 2 weeks (*n* = 15) of glasmacinal treatment. In all box plots, centre lines indicate medians, boxes indicate the 25th–75th percentiles, and whiskers extend to the most extreme values within 1.5 times the interquartile range. **A** Enterobacterales: a significant effect of visit was detected, *F* (3, 48) = 19.6, *P* = 1.94 × 10^−8^. Counts were lower after 1 week than at Baseline 1 (estimated difference = 2.37 logCFU, 95% CI 1.36–3.38; *t*(48) = 4.71, FDR-adjusted *P* = 4.36 × 10^−5^) and Baseline 2 (estimated difference =  2.00 logCFU, 95% CI 0.99–3.01; *t*(48) = 3.97, FDR-adjusted P = 3.59 × 10^−4^), and after 2 weeks than at Baseline 1 (estimated difference = 3.28 logCFU, 95% CI 2.27–4.30; *t*(48) = 6.52, FDR-adjusted *P* = 2.42 × 10^−7^) and Baseline 2 (estimated difference = 2.91 logCFU, 95% CI 1.90–3.93; *t*(48) = 5.78, FDR-adjusted *P* = 1.61 × 10^−6^). **B** *Enterococcus*: no effect of visit, *F* (3, 48) = 1.19, P = 0.324. **C** *Bacteroides*: no effect of visit, *F* (3, 48) = 1.20, *P* = 0.321. **D** *Lactobacillus:* no effect of visit, *F* (3, 48) = 0.50, *P* = 0.687. **E** Enterobacterales cultured on CHROMagar supplemented with cefotaxime (2 mg/L): growth was observed in only two samples,statistical analysis was not performed. **F** Enterobacterales cultured on CHROMagar supplemented with meropenem (2 mg/L): no growth, statistical analysis was not performed. **G** Enterobacterales cultured on CHROMagar supplemented with azithromycin (16 mg/L): a significant effect of visit was detected, *F* (3, 48) = 4.32, *P* = 0.00895. Counts were lower after 2 weeks than at Baseline 1 (estimated difference = 0.946 logCFU, 95% CI 0.29–1.60; *t*(48) = 2.89, FDR-adjusted *P* = 0.0174) and Baseline 2 (estimated difference = 0.945 logCFU, 95% CI 0.29–1.60; *t* (48) = 2.89, FDR-adjusted *P* = 0.0174). **H** Bacteroides cultured on BKV agar supplemented with azithromycin (16 mg/L): no effect of visit, F(3, 48) = 1.10, *P* = 0.359. **I** Bacteroides cultured on BKV agar supplemented with meropenem (2 mg/L): no effect of visit, *F*(3, 48) = 0.20, *P* = 0.89.

For the phenotypic analysis, clinically important taxa were selected: Enterobacterales, *Enterococcus* spp.*, Bacteroides* spp., *Lactobacillus* spp.*, Clostridioides difficile* (*C. difficile*), and *Candida* spp. Except for Enterobacterales, none of these taxa showed significant differences in the log count of colony forming units (log10CFU) after 1- or 2-weeks glasmacinal compared with either baseline time-point (Fig. 5). For Enterobacterales, there was a significant decrease in logCFU after 1- and 2-weeks glasmacinal compared with both baseline time-points. The relative abundance analysis demonstrated a reduction in the *Escherichia*-*Shigella* genera, which may explain the decrease in Enterobacterales.

Isolates with resistance to azithromycin were observed among Enterobacterales at all time-points (Baseline 1 [6 samples], Baseline 2 [5 samples], 1-week glasmacinal [3 samples], 2-weeks glasmacinal [1 sample]) and *Bacteroides* (Baseline 1 [10 samples], Baseline 2 [9 samples], 1-week glasmacinal [8 samples], 2-weeks glasmacinal [6 samples]). Furthermore, exploratory tests were conducted for the presence of resistant fractions of Enterobacterales and *Bacteroides* against other clinically important antibiotics: carbapenems (meropenem for Enterobacterales and *Bacteroides*) and the third-generation cephalosporins (cefotaxime for Enterobacterales). No meropenem or cefotaxime resistant isolates were observed in any of the samples. Selection of *C. difficile* or *Candida* spp. was not observed in any of the samples. The findings of all samples with growth are presented in Fig. 5 and participant-level quantitative culture measurements for all taxa, media, and study visits are provided in Supplementary Data 14.

## Discussion

This trial was a clinical evaluation of the effects of the anti-inflammatory macrolide, glasmacinal, on the gut microbiome, using both 16S rRNA gene sequencing and phenotypic methods. In vitro, glasmacinal has negligible antimicrobial activity^23^, and when administered to COPD patients for 3 months, had no detectable impact on the lung microbiome^24^. This trial aimed to assess the potential for glasmacinal to induce antibiotic-like gut dysbiosis and selection of resistance in healthy adults. Two weeks’ treatment of glasmacinal was associated with a transient reduction in phylogenetic richness, and modest phylogenetically structured shifts in community composition, without corresponding changes in evenness or evidence of community collapse. Quantitative culture analyses independently demonstrated stability of key anaerobic and commensal groups, alongside a reduction in Enterobacterales abundance, with no detectable emergence of resistance to azithromycin or expansion of opportunistic pathogens. Together, these findings indicate that glasmacinal exposure produced measurable, but limited, gut microbiome perturbations that are distinct from the hallmark signatures of dysbiosis induced by antibiotics (characterised by significant reduction in key commensal anaerobic phyla, expansion of taxa typically associated with antimicrobial selection, and increase in dominance of pathobionts, such as Proteobacteria^40,41^).

Glasmacinal administration was associated with a transient reduction in phylogenetic richness, indicating selective depletion or suppression of a subset of phylogenetically distinct taxa, while Pielou’s evenness remained stable, arguing against major reordering of relative abundances or dominance by a limited number of organisms. Beta-diversity analysis using weighted UniFrac revealed modest, phylogenetically structured shifts at 1-week, explaining approximately 10% of community variation., with attenuation to approximately 6% by the 2-week time-point, suggesting partial community adaptation rather than progressive divergence. Differential abundance analysis further demonstrated that changes were distributed across multiple phylogenetic lineages rather than concentrated within specific families or functional guilds, and they were not accompanied by expansion of known opportunistic or resistant taxa. Together with the absence of resistance selection in culture-based assays and the lack of sustained compositional collapse, these findings show that glasmacinal induced limited ecological perturbations without the defining hallmarks of antibiotic-like dysbiosis.

Although generalisation of the observed microbiome restructuring beyond this cohort cannot be assumed, the longitudinal trajectories of selected genera point to a coherent ecological pattern rather than a random fluctuation and are distinctly different to those associated with antibiotic macrolides. Several taxa that declined following glasmacinal treatment, including *Akkermansia*, *Sutterella*, *Parasutterella*, *Escherichia/Shigella*, and *Haemophilus*, are typically associated with the host-proximal niche of the gut^42–45^. *Akkermansia* has shown resistance to several antibiotics^46^, and has been reported to increase in the human gut following treatment with the antibiotic macrolides, azithromycin and erythromycin^47^. This was likely due to selective antibiotic pressure following macrolide exposure leading to conditions favouring *Akkermansia* expansion. In contrast, as glasmacinal lacks antibiotic activity^23^, ecological reshaping of the gut was limited, and the reduction in *Akkermansia* was potentially due to the immunomodulatory properties of glasmacinal. Macrolides, including glasmacinal, have been shown to reduce mucus^1,48^. Unlike most anaerobes, mucin is the main carbon source for Akkermansia in the gut, so a reduction in mucus could potentially lead to a decrease in the abundance of this genus, but not other anaerobes. Moreover, the genera that increased after treatment, such as *Bifidobacterium*, *Blautia*, *Anaerostipes*, and *Fusicatenibacter*, are strict anaerobic fermenters that dominate the luminal compartment of the colon and specialise in carbohydrate fermentation and short-chain fatty acid production^49^. This reciprocal pattern suggests a redistribution of microbial niches, with reduced representation of taxa adapted to host-derived substrates and conditions, alongside relative enrichment of metabolically fermentative taxa favoured in a stable, anaerobic environment. In contrast, the antibiotic macrolide, azithromycin, significantly reduced anaerobic taxa, including *Bifidobacterium longum*, when administered for 6-days to healthy participants^50^. Even though the anti-inflammatory and immunomodulatory effects of glasmacinal did not significantly disrupt the anaerobic taxa, competitive exclusion and antagonistic effects of *Bifidobacterium* (increased abundance in this study) might partly explain the reduction of clostridia (*Clostridium sensu stricto*-I)^51,52^. Importantly, *Bacteroides* and *Parabacteroides* remained largely stable across time-points, and unlisted genera were non-differentially abundant, indicating that glasmacinal induced selective rather than broad taxonomic changes. These shifts occurred without dominance by a single taxon or collapse of overall community structure, consistent with ecological rebalancing rather than disruption. Nonetheless, these interpretations are constrained by 16S rRNA gene resolution and lack of strain- or function-level profiling.

The current trial did not include post-treatment faecal collections, so no comment can be made about the recovery duration of the changes observed, which is a limitation of our trial design. However, the limited ecological reshaping of the gut following glasmacinal treatment is supported by the side effect profile observed in glasmacinal clinical trials to date. The impact of antimicrobials on the gut microbiome often manifests in gastrointestinal disturbance (such as diarrhoea), which is a commonly reported side effect of many antimicrobials, including azithromycin^53^. Notably, across the four clinical trials of glasmacinal conducted to date (including dosing of COPD patients for 3 months), the proportion of gastrointestinal adverse events reported after glasmacinal has been comparable to placebo^24,26,37^.

Antibiotic exposure is associated with selection of antibiotic resistance genes in both commensals and pathogens. In clinical trials assessing the long-term use of antimicrobial macrolides to treat respiratory diseases, emergence of macrolide resistance has been reported^12,13,54–56^. As glasmacinal is in development as a chronic treatment for COPD, a key part of this trial was to assess glasmacinal’s potential to induce antimicrobial resistance. Following 2 weeks’ of glasmacinal, there was no emergence of cross resistance to azithromycin (used as a class representative of macrolides), and selection of *Candida* spp. and *C. difficile* was not observed. Enterobacterales and *Bacteroides* spp. with resistance to azithromycin were detected at all time-points, with no change in logCFU counts of azithromycin resistant Bacteroides between time-points, but there was a significant reduction in azithromycin resistant Enterobacterales after 2-weeks glasmacinal. This was likely due to the significant decrease in Enterobacterales after glasmacinal, demonstrated by both quantitative culture and relative abundance analysis. Taken together, these data show that glasmacinal did not impose selection pressure over the 2 weeks of treatment.

Clinical trials that have assessed chronic use of azithromycin to treat respiratory conditions and have reported emergence of resistance to azithromycin, are typically a year or more^54–56^. Therefore, a limitation of this trial is the relatively short treatment period of 2 weeks compared with azithromycin trials. In one previous study, treatment of *Mycobacterium abscessus* infections for 2 weeks with azithromycin induced azithromycin resistance^57^. Another study conducted to evaluate the lag time in pharyngeal carriage of macrolide resistance after azithromycin and clarithromycin administration showed that the peak resistance was seen at day 8 in streptococci^58^. Considering that Enterobacterales and *Bacteroides* exhibit faster growth rates (24–48 h) than mycobacterial species and probably comparable growth rates to streptococci, the 2-week treatment period in this trial was likely sufficient for the detection of at least the earliest resistance phenotype. However, it should be noted that the mechanisms of resistance emergence in Enterobacterales are most likely different from *M. abscessus* and streptococci. As glasmacinal is intended as a chronic treatment, microbial testing in longer-term trials will be important.

In addition to the limitations described above (short treatment duration and lack of recovery sampling after cessation of treatment), this trial design has several further limitations. The faecal samples were collected during a drug-drug-interaction trial, so participants received a single dose of the probe substrates midazolam and digoxin 1 week before glasmacinal treatment was initiated. Both the phenotypic and 16S rRNA gene sequencing analyses suggest that midazolam and digoxin had no detectable impact on microbiome composition (Baseline 1 versus Baseline 2 comparison); however, studies have shown that many non-antibiotic drugs can impact the gut microbiome, including proton pump inhibitors, lipid-lowering agents, anti-hyperglycaemic drugs, non-steroidal anti-inflammatory drugs and pain-relieving agents^59,60^. Though we did not observe changes in diversity metrics between Baseline 1 and Baseline 2, it cannot be ruled out that the changes observed after glasmacinal treatment were influenced by midazolam and/or digoxin. A potential impact from midazolam and/or digoxin is supported by the finding that the significant changes observed in beta-diversity 1-week after glasmacinal are reduced after 2-weeks. A further limitation of the trial design is the lack of placebo control, which was unnecessary for the primary pharmacokinetic objective of the trial, but would have aided interpretation of the exploratory microbiology endpoints. However, the objective of the microbiology analysis was not to detect subtle microbiome perturbations, but rather to identify major disruptions akin to those of antibiotics. In this context, assessment of intra-participant changes was considered sufficient, and strengthened by two pre-treatment baseline time-points. This intra-participant control strengthens confidence that post-administration changes were attributable to glasmacinal rather than time alone. Overall, the results from this trial are considered preliminary and further microbial testing in an optimised trial design is needed (placebo-controlled, without administration of other medicinal agents, longer term dosing, and with a recovery sampling phase).

This trial was conducted in healthy participants with a median age of 34 years. This is a potential limitation, since the gut microbiome of healthy participants is likely different than in COPD patients (the intended patient population), who are typically over 40 years, take multiple concomitant medications and often have comorbidities. However, the trial represents an initial clinical assessment of the potential effects of glasmacinal on the gut microbiota, and although the baseline gut microbiota of a COPD patient may differ, the finding that glasmacinal induced limited ecological perturbations and did not impose selection pressure, is translatable to a COPD population. Interestingly, genotypic assessment of sputum from COPD patients treated for 3 months with daily 375 mg glasmacinal, showed no detectable impact on the lung microbiome^24^. The highest potential clinical dose of glasmacinal (375 mg) was tested in this trial, but if a lower dose is efficacious, the clinical dose will be reduced and the modest effects on microbial composition observed in this trial may also be reduced. Microbial testing will be continued in further longer-term trials of glasmacinal in the target population, at the clinical dose, and with an optimised trial design.

In conclusion, daily treatment of 375 mg glasmacinal for 2 weeks in healthy adults was associated with a transient reduction in phylogenetic richness, and modest phylogenetically structured shifts in community composition, without corresponding changes in evenness or evidence of community collapse (significant changes in approximately 10% of community variation at 1-week, and 6% at 2-weeks, were observed). There was no increase in the abundance of pathobionts and no loss of the major anaerobic phyla of the gut microbiota (Firmicutes and Bacteroidetes). Furthermore, emergence or selection of resistance against azithromycin was not observed, nor selection of clinically important *Candida* spp. or *C. difficile*. Overall, the trial showed that glasmacinal induced limited restructuring of the gut microbiome, without the defining hallmarks of antibiotic-like dysbiosis.

## Methods

### Trial design

An open-label clinical pharmacology trial (ClinicalTrials.gov: NCT06118684, registration date 20-Oct-2023 [https://www.clinicaltrials.gov/study/NCT06118684?term=NCT06118684&rank=1]; EU number: 2023-506345-34) was conducted in healthy participants at one site in Sweden (CTC Clinical Trial Consultants AB) from October 2023 to January 2024^37^. The trial was approved by the Swedish Ethical Review Board (25-Sep-2023) and included in the Clinical Trial Regulation approval issued by the Swedish Medical Product Agency (reference: 5.1.1-2023-59011). The trial was conducted in accordance with ICH Good Clinical Practice, and all participants provided full written informed consent. The participants received financial compensation for their time and study-related commitments in accordance with the policies of the clinical pharmacology unit (CTC Clinical Trial Consultants AB) and applicable local regulations. Compensation was not contingent upon completion of the study, and the compensation arrangements were reviewed and approved by the Swedish Ethical Review Board. The primary objective was to evaluate potential drug-drug interactions of glasmacinal (refer to the primary publication of the trial, which includes safety and tolerability data)^37^. An exploratory objective of the trial was to assess the effect of daily doses of glasmacinal (375 mg) for 2 weeks on the gut microbiome by collecting pre- and on-treatment faecal samples.

Participants received a single dose of 4 mg midazolam (oral solution, 1 mg/mL) and 0.25 mg digoxin (tablet, 0.25 mg) on Days 1 and 24. Glasmacinal (375 mg fasted for at least 10 h) was administered daily from Days 9–28. Participants took glasmacinal at home until Day 23, when they were admitted to the clinical site and the second midazolam/digoxin co-administration was given on Day 24 (after the last faecal sample had been collected). Glasmacinal was administered in capsules with excipients. Faecal samples were collected at the following 4 time-points (see Fig. 1):

- Baseline 1: prior to any trial treatments
- Baseline 2: 1 week after the first dose of midazolam/digoxin, but before starting glasmacinal dosing
- 1-week glasmacinal: after 6 to 7 days of glasmacinal daily
- 2-weeks glasmacinal: after 13 to 14 days of glasmacinal daily, and before the second dose of midazolam/digoxin

No formal microbiome-specific sample-size calculation was performed. The planned sample size of 18 participants was selected to meet the primary clinical objective of evaluating potential drug-drug interactions of glasmacinal, while also allowing an exploratory assessment of gut microbiome effects. The microbiome analysis was therefore designed to detect large, consistent antibiotic-like perturbations, rather than subtle, rare, transient, or highly heterogeneous microbiome changes.

To further evaluate whether the study design and analytical framework had sufficient sensitivity to detect large antibiotic-like microbiome perturbations, we performed a post-hoc sensitivity analysis, reported in detail in Supplementary information, section 2. This analysis used the longitudinal paired structure of the glasmacinal dataset as an internal sensitivity check and contextualised the observed effect sizes using external antibiotic benchmark datasets. The purpose was not to exclude all possible microbiome effects, but to assess whether the study could detect large, consistent within-subject changes relevant to antibiotic-like dysbiosis. Overall, the simulations confirm that the analyses were sufficiently sensitive to detect antibiotic-like dysbiosis had it occurred.

### Trial population

Healthy male and female participants with no ongoing medical conditions or significant medical history were included in the trial. Participant sex was recorded at screening based on self-report. Sex was not used as a stratification factor, and the trial was not designed or powered to evaluate sex-specific differences. All participants were non-smokers or former smokers who had stopped smoking (including e-cigarettes) for at least 6 months. Except contraceptives, regular use of other medications was prohibited. The eligible age range was 18 to 55 years, and the BMI range was 18 to 33 kg/m^2^.

### Faecal samples

In total, 62 faecal samples were collected and analysed from 17 participants. After collection, samples were stored at 4 °C in cold boxes until participants visited the clinic, when they were transferred to −80 °C. At the end of the trial, samples were shipped on dry ice (temperature controlled) to the Karolinska Institutet for analysis. Samples were stored for a maximum of 3 months before transfer to the Karolinska Institutet for analysis. All participants provided a baseline sample (Baseline 1) and were retained in the analysis irrespective of missing follow-up samples. The 15 participants that provided samples at all time-points were 100% compliant for glasmacinal intake via eDiary reporting, which was supported by glasmacinal plasma concentrations^37^. The total number of samples analysed per time-point were as follows (refer to Table 1 for trial disposition information, and Fig. 1).

- Baseline 1: 17 samples
- Baseline 2: 15 samples
- 1-week glasmacinal: 15 samples
- 2-weeks glasmacinal: 15 samples

### Phenotypic analysis

*Bacteroides*, Enterobacterales, *Enterococcus*, and *Lactobacillus* were selected as strategic sentinel taxa because they represent key ecological and clinical dimensions of macrolide-induced gut microbiome perturbation, including community stability^61,62^, dysbiosis^63^, antimicrobial resistance reservoirs^64^, and beneficial microbial function^65,66^. *Enterococcus* and *Lactobacillus* were also specifically selected for quantitative culture because they may be under-detected by sequencing due to DNA extraction^67^ and lysis limitations^68^. Other taxa that are important for gut health, such as *Faecalibacterium* and *Bifidobacterium*, were not selected for quantitative culture because they are better assessed via sequencing-based approaches, and because they are either difficult to culture reliably (e.g. *Faecalibacterium*^69^) or are less directly associated with macrolide resistance dynamics (e.g. *Bifidobacterium*^70,71^). Overall, the selected taxa provide clinically relevant and cultivable markers for monitoring antibiotic-associated microbiome disruption, recovery, and resistance emergence across both Gram-positive and Gram-negative bacteria^66,70^. Selection/emergence of resistance to azithromycin and related antimicrobials was also assessed; for this purpose, media supplemented with azithromycin (16 mg/L) were used to test emergence of acquired resistance (MIC > 16 mg/L) to the class of macrolides in Enterobacterales and *Bacteroides*^72,73^ (as indicators of aerobic and anaerobic taxa, respectively).

### Culture media and antibiotics

All culture media were manufactured at the Karolinska University Hospital (Stockholm, Sweden). The substrate unit at Karolinska University Hospital is a nationally accredited centre for production of laboratory consumables for diagnostic services (https://www.karolinska.se/for-vardgivare/karolinska-universitetslaboratoriet/klinisk-mikrobiologi/substrat/). Customised culture media were used for phenotypic studies of selectively targeted clinically relevant taxa, and for detecting selection/emergence of resistance to azithromycin and/or potential overgrowth of *Candida* spp. and *Clostridioides difficile*. For selection/emergence of resistance, culture media supplemented with azithromycin (16 mg/L), meropenem (2 mg/L), or cefotaxime (2 mg/L), were used.

### Sample processing and colony counting

Faeces (0.5 g) were weighed using a scale and suspended in 4.5 mL of pre-reduced peptone yeast extract broth. The suspension was serially diluted, and from each dilution, 100 µL of the suspension was plated on several selective and differential media with and/or without antibiotics. The number of colonies were counted after overnight incubation for non-fastidious Gram-negative bacteria, and after 48 h for anaerobic bacteria. Colonies were counted by focusing on typical morphologies and, where necessary, using MALDI-TOF to differentiate those with atypical appearances. Rogosa agar was prepared strictly at pH 5.5, which significantly reduces non-Lactobacillus species. Gram-staining was not used as it is generally unreliable and difficult to apply to every colony on the plate. On some culture plates a few small non-lactobacilli colonies identified as enterococci, and pediococci were confirmed by MALDI-TOF and excluded during counting, as they represented only few colonies per plate. Similarly, Prevotella species that were encountered on BKV, were excluded from counting. Where different Bacteroides species were identified on the same plate, all were counted together as Bacteroides. The colony counts were accounted for dilution, volume correction factor, log-transformed, and analyzed using linear mixed-effects models with time-point as a fixed effect and subject ID as a random intercept. Analyses were performed in R (version 4.4.2) using lme4 (version 1.1-36), with pairwise comparisons conducted via estimated marginal means and false discovery rate (FDR) correction using emmeans (version 1.10.7). Data visualisation was carried out with ggplot2 (version 4.0.0).

### 16S rRNA gene sequencing method and analysis

#### Sample processing, DNA extraction, and sequencing

The DNA isolation was performed using the Norgen stool DNA isolation kit (Thorold, Ontario, Canada). The method employs bead beating and isolation of the DNA based on spin-column chromatography. The manufacturer’s instructions were strictly followed. The procedure is summarised as follows: 200 mg faecal sample was weighed and transferred to the bead tube, and after adding the lysis buffer the sample was vortexed for homogenisation of the sample in the bead tube. The homogenised sample was treated with bead beating with Vortex Genie-2 at 2700 RPM and 60 Hz for 5 min. Purification of the DNA was performed by spin-column isolation following the manufacturer’s instruction. The purified DNA was measured with Nanodrop for quantity and purity, contamination of RNA fragments or proteins. Good quality DNA material from each sample was sent for sequencing to the vendor.

### Library construction and sequencing

For the 16S rRNA, a qualified total genomic DNA was used to prepare the sequencing library, and sequencing was done at the BMK (Biomarker Technologies GmbH, Munster, Germany). The hypervariable region V3-V4 of the bacterial 16S rRNA gene were amplified with primer pairs 338 F: 5’- ACTCCTACGGGAGGCAGCA-3’ and 806 R: 5’- GGACTACHVGGGTWTCTAAT-3’. PCR products were checked on agarose gel and purified through the Omega DNA purification kit (Omega Inc., Norcross, GA, USA). The purified PCR products were collected, and the paired-ends sequencing (2 × 250 bp) were performed on the Illumina Novaseq 6000 platform.

### Statistics and bioinformatic analyses

The Illumina paired-end reads were processed using the QIIME 2 pipeline (version 2024.10). Initially, they were merged using the ‘merge-pairs’ plugin, which employs the VSEARCH algorithm to join forward, and reverse reads based on overlapping regions. Quality filtering was then performed by ‘quality-filter’ plugin, removing low-quality reads based on Phred quality scores Q30 threshold. Denoising of the merged sequences was conducted using Deblur algorithm with a trim length of 400 base pairs, which resolve ASVs by removing sequencing errors and chimeras. Finally, a total of 6,939,567 reads were retained across all samples, with a mean of 111,927reads per sample (range:74,470–173069). The meta-data and detailed counts of filtered and merged reads for each sample are provided in Supplementary Data 8 and 9.

To construct a phylogenetic tree, ASVs were aligned using the QIIME ‘phylogeny’ plugin. This command performs multiple sequence alignment with MAFFT, followed by phylogenetic tree construction using FastTree. Core diversity metrics, including alpha diversity and beta diversity were calculated using QIIME core-metrics-phylogenetic plugin. This analysis incorporated phylogenetic relationships and was based on a rarefied feature table, with sampling depth 74,470 to retain all 62 samples.

Alpha diversity was assessed using Faith’s phylogenetic diversity (PD) and Pielou’s evenness to separately capture richness and evenness components of within-sample diversity. Faith’s PD was selected to quantify phylogenetic richness by incorporating evolutionary relatedness among taxa, increasing sensitivity to gains or losses of distinct lineages. Pielou’s evenness was used to assess how uniformly taxa were distributed within samples, independent of richness. Alpha diversity measures were analysed using linear mixed-effects models similar to those applied to the quantitative culture data mentioned above.

Beta-diversity was assessed using weighted UniFrac distance, which incorporates both relative abundance and phylogenetic relatedness among ASVs. This metric was selected a priori because it is well suited to detecting coordinated community shifts across related lineages, rather than purely compositional turnover driven by changes in individual taxa. Differences between time-points were evaluated using pairwise permutational multivariate analysis of variance (PERMANOVA) implemented with the pairwise.adonis2 function from the pairwiseAdonispackage (0.4), which uses adonis2 from vegan (version 2.6-10). To account for the longitudinal study design, permutations (*n* = 9999) were constrained within participants using participant ID as a strata variable. Resulting *p*-values were adjusted for multiple testing using the FDR method.

A custom taxonomic classifier was trained using dereplicated sequences and taxonomic annotations from the Silva 138 database, specific to the V3-V4 hypervariable region of the 16S rRNA gene. Details of the training process are available in the repository (GitHub: anw-sh/silva-138_classifiers, last access: February 2025). The classifier’s performance was evaluated with QIIME rescript evaluate-fit-classifier command, which assesses the accuracy and fit of the trained model. Taxonomic classification of ASVs was then performed using QIIME feature-classifier, leveraging a scikit-learn-based naïve Bayes classifier optimised for microbiome data.

Differential abundance analysis was conducted using LinDA library (version 0.2.0) and ANCOM-BC2 (version 3.20) in R. Analyses were first performed at the ASV level and then repeated after aggregation to higher taxonomic ranks. Prior to modelling, taxa were filtered by prevalence, retaining features present in at least 30% of baseline samples or at least 30% of on-treatment samples, allowing inclusion of taxa that emerged or disappeared following treatment while excluding sparsely detected features. For both methods, time-point was specified as a fixed effect and participant ID as a random intercept to account for within-participant correlation in the longitudinal design. LinDA was applied to unrarefied count data and accounted for sequencing depth and compositional effects through its centred log-ratio-based bias-correction framework. No additional prevalence or library-size filtering was performed within LinDA, and values were winsorized at the 97th percentile to reduce the influence of extreme counts. ANCOM-BC2 was applied to the same prevalence-filtered, unrarefied abundance tables using the same fixed- and random-effects structure. No additional prevalence filtering was performed within ANCOM-BC2, and no samples were excluded because of library size. Variance regularisation was applied using the 10th percentile of the standard-error distribution, and pseudo-count sensitivity analysis was enabled to assess the robustness of significant findings to alternative zero-replacement values. Structural-zero detection was performed across time-points, with the negative lower-bound criterion enabled. For both methods, the *p*-values were adjusted for multiple testing using the FDR procedure. To facilitate direct comparison between methods, all effect estimates were converted where necessary and reported as log2 fold changes. Taxa were considered differentially abundant when the absolute log2 fold change exceeded 1 and the adjusted *p*-value was below 0.05. For ANCOM-BC2, associations were additionally required to pass the pseudo-count sensitivity criterion.

Moreover, the filtered ASVs generated in QIIME2 were aligned using MAFFT to produce a multiple sequence alignment, which was then used to construct a phylogenetic tree with FastTree using approximately maximum-likelihood estimation (Supplementary Data 1). The resulting tree was visualised and annotated in iTOL. To formally assess whether ASV-level responses were concentrated within specific regions of the phylogeny, we performed a permutation-based phylogenetic dispersion analysis in R using ape (v5.8-1). All ASVs retained in the ASV-level LinDA analysis were used as the background set, and all FDR-significant ASVs from the 1-week and 2-weeks comparisons were combined as the foreground set. We calculated the observed mean pairwise phylogenetic distance (MPD) among foreground ASVs using branch-length distances from the ASV tree (cophenetic.phylo). This observed MPD was compared with a null distribution generated by randomly sampling the same number of ASVs from the background set 9999 times and recalculating MPD for each random set. Lower-than-expected MPD indicated phylogenetic clustering, whereas higher-than-expected MPD indicated phylogenetic dispersion. Standardised effect size was calculated as *(observed MPD - mean null MPD) / SD null MPD*, and two-sided permutation *p*-values were calculated for clustering and dispersion.

For visualisation of microbial abundance patterns, CLR transformation was applied to ASV or aggregated taxon counts. CLR normalisation accounts for the compositional nature of sequencing data by expressing each feature relative to the geometric mean of all features in a sample, thereby enabling comparison of relative changes across taxa and time-points. Positive CLR values indicate taxa enriched relative to the sample median, whereas negative values indicate taxa depleted relative to the sample median. CLR-transformed abundances were used to illustrate longitudinal trajectories and patterns of taxonomic shifts across time-points.

As a sensitivity analysis, the Deblur-derived ASV table was corrected for predicted 16S rRNA gene copy number using RasperGade16S^74^. The corrected table was re-analysed using the same downstream diversity and differential-abundance workflow as the primary uncorrected analysis. The analysis results are listed in Supplementary Data 10−13 and presented in Supplementary information, section 1.

### Reporting summary

Further information on research design is available in the Nature Portfolio Reporting Summary linked to this article.

## Supplementary information

Supplementary information

Description of additional supplementary files

Supplementary Data 1

Supplementary Data 2–14

Reporting Summary

Transparent Peer Review file

## Data Availability

Source data file and additional supplementary information is provided in the Supplementary Data (Source_data_glasmacinal).xlsx, and supplementary information.pdf. The 16S rRNA gene sequencing data generated in this study have been deposited in the NCBI Sequence Read Archive under BioProject accession code PRJNA1377574: https://www.ncbi.nlm.nih.gov/bioproject/PRJNA1377574.

## Code availability

The code used for microbiome processing, statistical analysis and figure generation in this study is available in the q2-pipeline GitHub repository: https://github.com/Karolinska-AMR/q2-pipeline. The version used in this study has been permanently archived in Zenodo.^75^
