# Supplementary material for: Impact of the anti-inflammatory macrolide glasmacinal on the gut microbiota of healthy adults: an open-label trial
Source: Nat Commun. 2026 Aug 20;17:8728. doi: 10.1038/s41467-026-76867-9 (PMC13493899; doi:10.1038/s41467-026-76867-9)
Supplement: Supplementary file 1 [file 41467_2026_76867_MOESM1_ESM.pdf]

Supplementary information 1

This supplementary document presents two complementary analyses evaluating the robustness and sensitivity of the microbiome findings. Section 1 examines whether correction for predicted 16S rRNA gene copy-number altered the principal diversity and differential-abundance results. Section 2 assesses the ability of the study design and analytical framework to detect large, coordinated antibiotic-like microbiome perturbations.

***

Table of contents

Section 1: Effect of 16S rRNA Gene Copy-Number (GCN) Correction on Study Conclusions 2

1. BACKGROUND 2
2. METHODS 2
   - 2.1. Copy-Number Correction and Feature Table Preparation 2
   - 2.2. Rarefaction and Alpha Diversity Analyses 2
   - 2.3. Beta Diversity Ordination and Distance Analysis 2
   - 2.4. Differential Abundance Analysis 3
   - 2.5. Data Integration and Visualization 3
3. RESULTS 3
   - 3.1. Feature Table 3
   - 3.2. Alpha Diversity 4
   - 3.3. Beta Diversity 6
   - 3.4. Differential Abundance 8

Section 2: Analytical Power to Detect Antibiotic-like Dysbiosis in the Glasmacinal Microbiome Study 11

1. BACKGROUND 11
2. METHODS 11
3. RESULTS AND DISCUSSION 13
   - 3.1. Internal sensitivity of the glasmacinal alpha-diversity analysis 13
   - 3.2. Antibiotic-Associated Alpha-Diversity Dysbiosis 14
   - 3.3. Beta-diversity: internal sensitivity and community-level benchmark 16
   - 3.4. Differential-abundance sensitivity analysis 18

References 20

Section 1: Effect of 16S rRNA Gene Copy-Number (GCN) Correction on Study Conclusions

1. Background

16S rRNA amplicon sequencing estimates microbial abundance from marker-gene counts rather than genome counts. Because bacterial taxa vary in the number of 16S rRNA gene copies per genome, taxa with higher copy numbers may be overrepresented in uncorrected relative-abundance profiles. To assess whether this potential bias influenced the conclusions of the primary analysis, we performed a sensitivity analysis using RasperGade16S[1], which adjusts ASV-level abundance estimates by dividing counts by taxonomically predicted 16S rRNA gene copy numbers derived from databases such as rrnDB. The corrected feature table was then analysed using the same downstream workflow as the original uncorrected dataset. Results from the two pipelines were compared across four analytical domains: feature-table composition, alpha diversity, beta diversity, and differential abundance.

2. Methods

2.1. Copy-Number Correction and Feature Table Preparation

The original Deblur-derived amplicon sequence variant (ASV) feature table, comprising 62 samples and 7,644 ASVs from 16S rRNA gene V3-V4 sequencing, was used as the input for copy-number correction. Copy-number correction was performed using RasperGade16S (https://github.com/wu-lab-uva/RasperGade16S), following the package workflow. RasperGade16S estimates 16S rRNA gene copy number and adjusts ASV-level abundance estimates by dividing observed read counts by the predicted copy number for each ASV.

The resulting corrected feature table was exported in BIOM v2.1.0 format and re-imported into QIIME 2 v2024.5 using the feature-table import plugin. The corrected table was then subjected to the same downstream analytical workflow as the original uncorrected table, enabling direct comparison between the two analysis branches.

2.2. Rarefaction and Alpha Diversity Analyses

Copy-number correction systematically reduces raw read counts by dividing each count by the predicted GCN (typically 1.5–5 for dominant gut taxa). The corrected feature table was therefore rarefied to 18,000 reads per sample. This depth was selected a priori to maximize sample retention while accounting for the reduced library sizes after copy-number correction. Rarefaction and alpha-diversity estimation were performed in QIIME 2 using the diversity core-metrics-phylogenetic pipeline.

Per-sample alpha-diversity values were exported as QIIME 2 SampleData[AlphaDiversity] artifacts and imported into R using the qiime2R package v0.99.6. Alpha-diversity metrics were analysed using the same statistical framework as described for the primary analysis in the main manuscript. Concordance between the original and corrected analyses was assessed using Pearson and Spearman correlation coefficients and visualized with ggplot2 v3.4.3.

2.3. Beta Diversity Ordination and Distance Analysis

Weighted UniFrac distance matrices and PCoA ordinations were generated for both branches in QIIME 2. Concordance was assessed using two complementary approaches. Procrustes analysis (protest,

vegan package) evaluates whether the low-dimensional ordination structures, the sample configurations that underpin the study's biological interpretation, are preserved after correction. Mantel tests (mantel, vegan) evaluate correlation of raw pairwise distance values across the full NxN matrix. Pairwise PERMANOVA was performed using the same approach as the primary analysis, with 9,999 permutations.

2.4. Differential Abundance Analysis

Differential-abundance analysis was performed using LinDA in R, following the same modelling strategy as described in the main manuscript. Analyses were conducted separately for the original and copy-number-corrected feature tables.

For each branch, differential abundance was tested across six taxonomic levels: ASV, genus, family, order, class, and phylum. The following visit contrasts were evaluated: Baseline 1 (BL1) versus Baseline 2 (BL2), BL1 versus 1-week glasmacinal (1W), BL1 versus 2-weeks glasmacinal (2W), BL2 versus 1W, BL2 versus 2W, and 1W versus 2W. Taxa with an adjusted p-value < 0.05 and an absolute log fold change > 1.0 were considered significant.

To evaluate concordance between pipelines, log fold-change estimates from the original and corrected analyses were compared at each taxonomic level using Pearson and Spearman correlation coefficients. The number of significant taxa was summarized by taxonomic level and visit contrast for each branch. Taxa identified as significant in only one branch were catalogued to identify copy-number-sensitive findings.

2.5. Data Integration and Visualization

All QIIME 2 artifacts were parsed into R using the qiime2R package (version 0.99.6). Downstream analyses, figure generation, and result tabulation were conducted in R (version 4.3.2) using the following packages: tidyverse (v2.0.0) for data manipulation, ggplot2 (v3.4.3) for publication-quality graphics, patchwork (v1.1.2) for faceted plot composition, vegan (v2.6.4) for multivariate statistics, lme4 (v1.1.35) and pbkrtest (v0.5.2) for mixed-effects modelling, emmeans (v1.8.5) for post-hoc comparisons.

3. Results

3.1. Feature Table

16S rRNA gene-copy-number correction substantially reduced the effective feature-table abundance, as expected, because ASV counts were divided by their predicted 16S rRNA gene copy number (Table S1 and Figure S1). Total abundance decreased from 6,939,567 to 1,709,049 copy-number-adjusted counts, corresponding to a 75.4% reduction. The number of samples was unchanged (62 samples), and almost all ASVs were retained after correction (7,644 vs 7,626 ASVs; 99.8% retained). Per-sample library sizes were reduced consistently across the dataset, with the median depth decreasing from 111,632 to 27,052 corrected counts (75.8% reduction). Despite the marked reduction in total abundance, the ASV composition was essentially unchanged: 7,626 of 7,644 ASVs were retained after correction (99.8%), indicating that 16S gene-copy-number correction primarily rescaled feature abundances rather than altering the underlying ASV repertoire.

Table S1. Feature-table summary before and after 16S gene-copy-number correction.

Parameter	Original	GCN-corrected	Change
**Total abundance**	6,939,567	1,709,049	-75.40%
**Total ASVs**	7,644	7,626	-0.20%
**Samples**	62	62	0%
**Min. depth (reads)**	74,470	18,818	-74.80%
**Median depth (reads)**	111,632	27,052	-75.80%
**Max. depth (reads)**	173,069	46,162	-73.30%

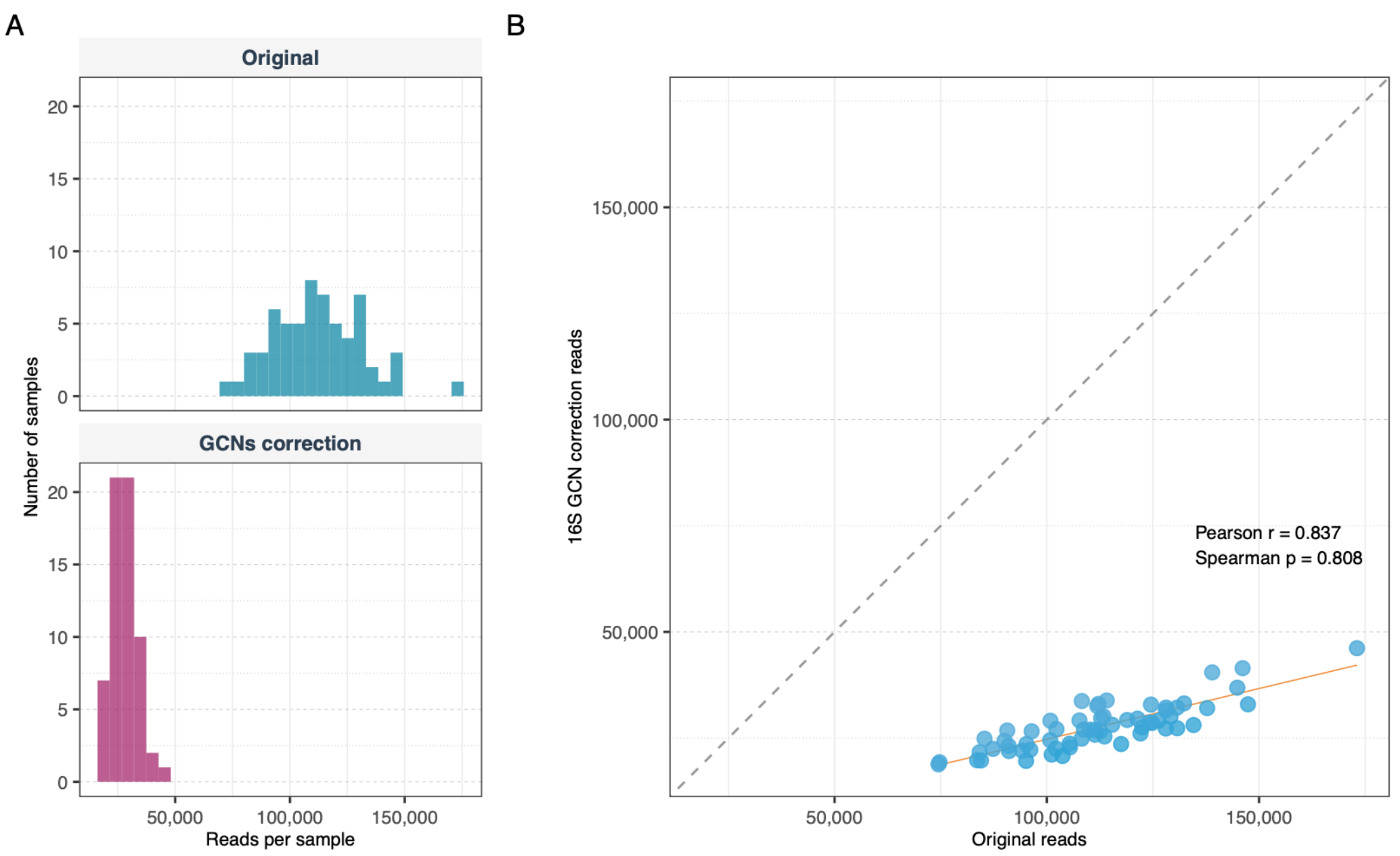

Figure S1. Effect of 16S rRNA gene-copy-number correction on feature-table depth. (**A**) Distribution of per-sample feature-table depth before and after RasperGade16S correction. The median depth decreased from 111,632 to 27,052 after correction. (**B**) Original and corrected sample depths remained strongly correlated, indicating that copy-number correction reduced absolute abundance values while broadly preserving the relative ordering of sample library sizes.

3.2. Alpha Diversity

Alpha diversity values were strongly correlated between branches for both metrics (Table S2, Figure S2). The Spearman correlation for Pielou's evenness (ρ = 0.81) was somewhat lower than for Faith's PD (ρ = 0.99), reflecting greater sensitivity of the evenness metric to the subtle compositional reweighting introduced by GCN correction. However, the mean absolute difference in evenness scores was negligible (0.01 units on a [0,1] scale), confirming that the reweighting did not shift individual sample estimates in a biologically meaningful way.

The primary statistical conclusions from the linear mixed-effects models were unchanged after correction (Table S3). The timepoint effect for Faith’s PD remained significant in both branches, with similar F-statistics and p-values. Pielou’s evenness remained non-significant in both analyses. Across the pairwise timepoint contrasts, no contrast changed significance status after copy-number

correction (Table S4). Notably, the treatment-associated increases in Faith's PD at 1W and 2W relative to both baselines were reproduced with comparable or slightly stronger effect estimates in the GCN-corrected branch, confirming that the richness signal is not an artefact of copy-number bias.

Table S2. Per-sample alpha diversity correlation between original and GCN-corrected branches.

Metric	Pearson r	Spearman ρ	Mean difference
**Faith's PD**	0.98	0.99	0.60 units
**Pielou's evenness**	0.81	0.81	0.01 units

Table S3. Linear mixed-effects model results for the overall effect of visit on alpha diversity before and after GCN correction. Models included visit as a fixed effect and participant as a random intercept. The table reports omnibus F-statistics and corresponding upper-tail, one-sided p-values for Faith's phylogenetic diversity and Pielou's evenness in the original and gene-copy-number-corrected analyses.

Metric	F (original)	p (original)	F (GCN)	p (GCN)
**Faith's PD**	31.36	8.0E-11	29.84	1.6E-10
**Pielou's evenness**	1.11	0.355	0.67	0.57

Table S4. Pairwise contrast comparisons of alpha diversity metrics between timepoints (Baseline 1 (BL1), n = 17; Baseline 2 (BL2), n = 15; week 1 (1W), n = 15; and week 2 (2W), n = 15). Differences between visits were assessed using two-sided linear mixed-effects models with participant as a random intercept, followed by two-sided estimated-marginal-means comparisons with Benjamini–Hochberg false-discovery-rate adjustment.

Metric	Contrast	Estimate (original)	P-value (original)	Estimate (GCN)	P-value (GCN)	Significance changed
Faith's PD	**BL1 – BL2**	-0.189	0.695	0.008	0.685	No
	**BL1 – 1W**	3.444	2.27E-08	3.694	8.80E-08	No
	**BL1 – 2W**	3.051	1.71E-07	3.402	1.41E-07	No
	**BL2 – 1W**	3.633	2.27E-08	3.685	8.80E-08	No
	**BL2 – 2W**	3.24	1.54E-07	3.394	8.80E-08	No
	**1W – 2W**	-0.393	0.522	-0.292	0.828	No
Pielou's evenness	**BL1 – BL2**	0.016	0.499	0.016	0.7	No
	**BL1 – 1W**	0.003	0.865	0.005	0.94	No
	**BL1 – 2W**	0.005	0.865	0.003	0.995	No
	**BL2 – 1W**	-0.013	0.499	-0.012	0.713	No
	**BL2 – 2W**	-0.011	0.499	-0.014	0.7	No
	**1W – 2W**	0.002	0.865	-0.002	0.94	No

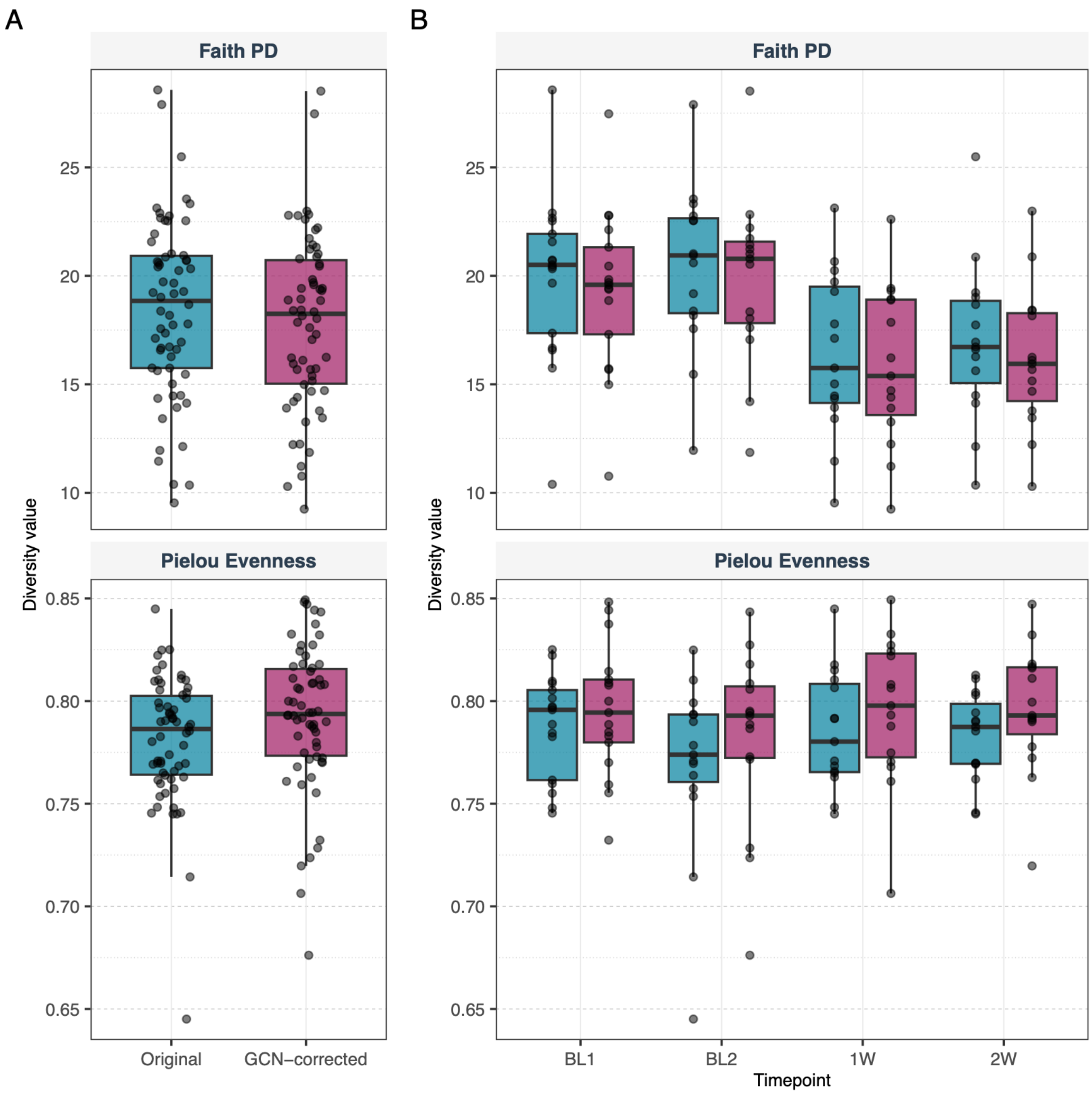

Figure S2. Concordance of alpha diversity metrics between original and GCN-corrected pipelines. (**A**) Side-by-side boxplots of Faith's PD (richness) and Pielou's evenness index for all 62 samples in both branches. (**B**) Metrics stratified by timepoint, showing that the biological patterns across the study schedule were preserved identically between branches. All boxplots show medians as centre lines, the 25th–75th percentiles as boxes, and the most extreme values within 1.5 times the interquartile range as whiskers.

3.3. Beta Diversity

Beta diversity was compared between the original and 16S GCN-corrected branches using weighted UniFrac distances, the beta-diversity metric most likely to be affected by this correction because it incorporates both phylogenetic relatedness and relative abundance. Overall, the corrected analysis preserved the same biological interpretation as the original analysis.

Visual inspection of the weighted UniFrac PCoA ordinations showed highly similar sample configurations before and after GCN correction (Figure S3A and S3B). In both branches, baseline samples clustered closer to each other, whereas treatment-period samples showed a modest displacement from baseline. This indicates that copy-number correction did not fundamentally alter the perceived community structure or the within-participant temporal trajectory.

Pairwise PERMANOVA results were largely concordant between branches (Table S5 and Figure S3C). The strongest treatment-associated separation was observed at 1 week, with BL1–1W and BL2–1W significant in both analyses. The corresponding R^2 values were only modestly reduced after GCN

correction, from 0.1044 to 0.0988 for BL1–1W and from 0.0670 to 0.0627 for BL2–1W. The BL1–2W comparison was also significant in both branches, with similar effect sizes before and after correction (R^2 = 0.0588 and 0.0526, respectively). By contrast, the 1W–2W comparison remained non-significant in both branches and had low R^2 values (0.0216 original; 0.0184 GCN), indicating that the on-treatment community configuration did not continue to diverge strongly between week 1 and week 2.

The only discordant pairwise result was the BL2–2W comparison. In the original branch this contrast was close to the significance threshold but did not pass FDR correction (R^2 = 0.0407, adjusted p = 0.0642), whereas after GCN correction it became significant with a very similar effect size (R^2 = 0.0478, adjusted p = 0.0121). This discrepancy should therefore be interpreted as a borderline, threshold-dependent result rather than a qualitative disagreement between pipelines. The small difference in R^2 indicates that GCN correction slightly strengthened the estimated BL2–2W separation but did not create a new large beta-diversity effect.

Procrustes analysis further supported strong concordance between the original and GCN-corrected ordination spaces (Figure S3D). After optimal rotation and scaling, the two ordinations remained closely aligned (Procrustes M^2 = 0.1626, r = 0.9151, Protest p = 0.0001), confirming that the overall spatial arrangement of samples was preserved after copy-number correction. Taken together, the beta-diversity sensitivity analysis shows that 16S GCN correction does not change the study conclusion. Glasmacinal exposure was associated with modest but detectable community restructuring, strongest at 1-week and still evident at 2-week in baseline comparisons, without evidence that GCN correction revealed a qualitatively different or broader dysbiosis pattern.

Table S5. Pairwise non-directional PERMANOVA results based on weighted UniFrac distances before and after 16S rRNA gene copy-number correction. Sample sizes at each time point are: Baseline 1 (BL1), n = 17; Baseline 2 (BL2), n = 15; 1-week (1W), n = 15; and 2-week (2W), n = 15.

Comparison	R^2 (original)	P.Adj (original)	R^2 (GCN)	P.Adj (GCN)	Sig. Changed
**BL1 – BL2**	0.0099	0.7977	0.006	0.9182	No
**BL1 – 1W**	0.1044	0.0018	0.0988	0.0006	No
**BL1 – 2W**	0.0588	0.028	0.0526	0.0056	No
**BL2 – 1W**	0.067	0.0016	0.0627	0.0012	No
**BL2 – 2W**	0.0407	0.0963	0.0478	0.0121	Yes
**1W – 2W**	0.0216	0.2725	0.0184	0.2836	No

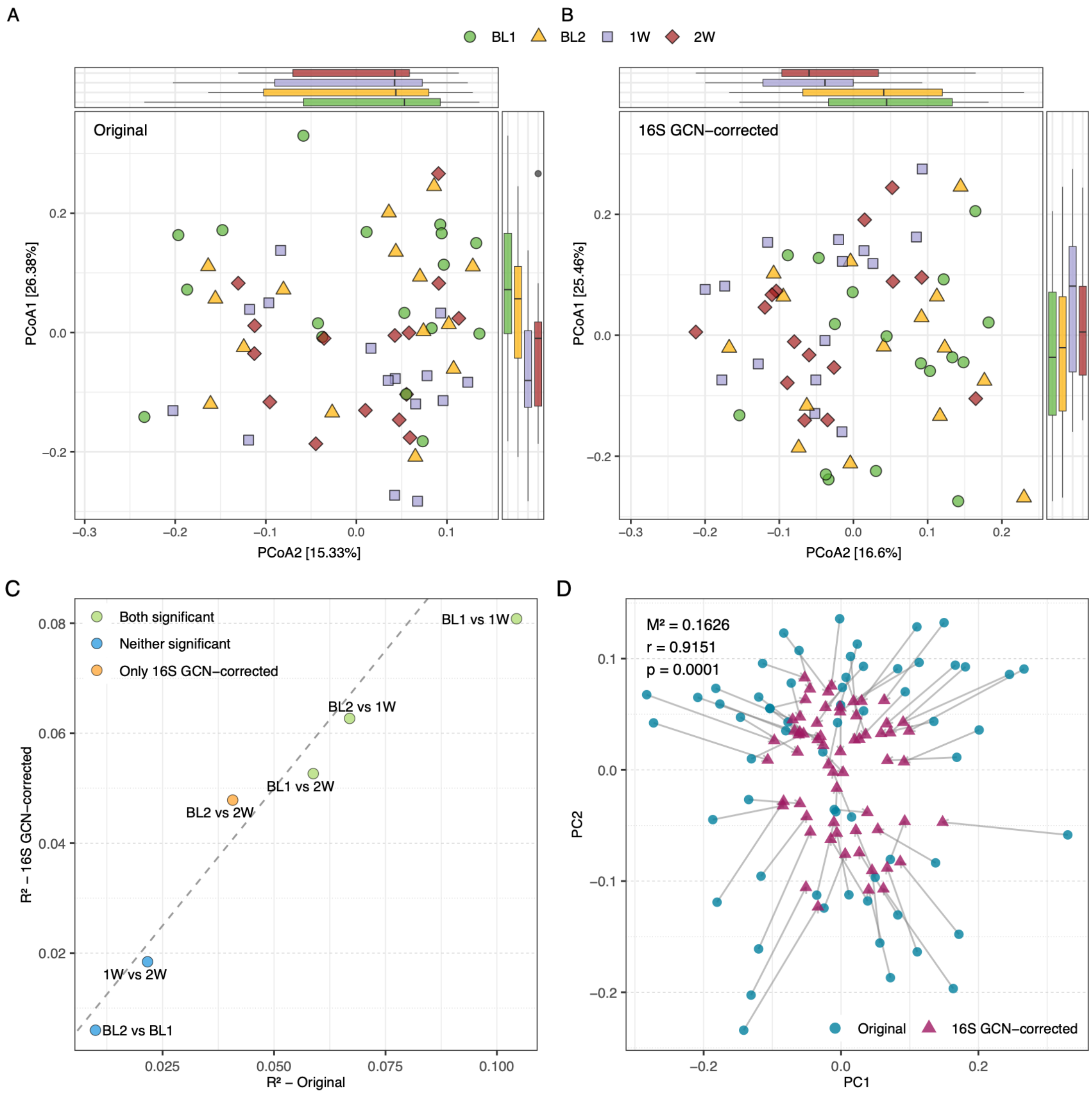

Figure S3. Beta diversity ordinations and PERMANOVA consistency between original and RasperGade16S-corrected pipelines. Weighted UniFrac PCoA ordinations are shown for the original (**A**) and 16S GCN-corrected (**B**) feature tables. marginal box plots summarizing their distributions along each axis. Box centres indicate medians, boxes the 25th–75th percentiles, and whiskers the most extreme values within 1.5 times the interquartile range. (**C**) Pairwise PERMANOVA R^2 values comparing original and corrected branches. Most contrasts showed concordant significance status and similar effect sizes; the BL2–2W comparison crossed the significance threshold only after correction, consistent with a borderline contrast rather than a major change in beta-diversity structure. (**D**) Procrustes analysis comparing the two ordination spaces showed strong concordance after optimal rotation and scaling ($M^2 = 0.1626$, $r = 0.9151$, one-sided Protest $p = 0.0001$), indicating that copy-number correction preserved the overall beta-diversity structure.

3.4. Differential Abundance

Differential abundance results were broadly concordant between the original and RasperGade16S-corrected branches. Across taxonomic levels, log fold-change estimates remained strongly correlated between analyses, indicating that 16S gene copy-number correction did not substantially alter the direction or relative ranking of treatment-associated abundance changes (Table S6). Thus, the correction did not generate a conflicting biological interpretation, but mainly reweighted the strength of evidence for individual taxa.

Table S6. Concordance of log fold-change estimates between the original and 16S gene copy-number corrected analyses. Pearson and Spearman correlations were calculated across taxa detected as significant in either branch at each taxonomic level.

Level	N taxa	Pearson r	Spearman ρ
**Phylum**	7	0.9969	0.9429
**Class**	29	0.9869	0.9585
**Order**	87	0.9858	0.9719
**Family**	49	0.9763	0.9119
**Genus**	14	0.994	0.9879
**ASV**	211	0.9798	0.922

The number of significant taxa showed a level-dependent pattern (Table S7). At the ASV level, the corrected branch identified more significant features than the original analysis, particularly at 2W, where the number of significant ASVs increased from 72 to 108. This is expected because GCN correction is applied directly at the ASV level. By dividing each ASV by its predicted 16S rRNA gene copy number, the correction can reduce inflation of high-copy ASVs while increasing the relative contribution of lower-copy ASVs. As a result, some fine-resolution ASV-level changes that were previously close to the detection threshold may become significant after correction.

In contrast, this increase in significant ASVs did not translate into more significant taxa at higher ranks. At genus, family, order, class, and phylum levels, the number of significant taxa was generally similar or lower after correction, especially at 2W (Table S7). This apparent discrepancy is not contradictory. Higher-level analyses aggregate multiple ASVs within the same genus or family, and those ASVs may differ in predicted copy number, direction of change, and effect size. After correction, ASV-level signals can therefore partially cancel when collapsed to genus or family level. In addition, if a higher-rank taxon was originally driven by a subset of high-copy-number ASVs, copy-number correction can attenuate the aggregate signal and move the taxon below the FDR threshold.

Importantly, taxa that changed significance status after correction generally retained the same direction of effect, suggesting attenuation or redistribution of statistical support rather than reversal of the observed abundance pattern. Genera that were significant in the original analysis but fell below the corrected threshold should therefore not be interpreted as false or biologically irrelevant; rather, they represent copy-number-sensitive or near-threshold associations that require more cautious interpretation. This is particularly relevant for genera such as *Bacteroides*, *Parabacteroides*, and *Fusicatenibacter*, where directionally consistent changes were observed but statistical support was reduced after correction. For *Bacteroides*, this attenuation is also consistent with the phenotypic culture data, where *Bacteroides* counts did not differ significantly across treatment time points in the main analysis.

Overall, The RasperGade16S analysis confirmed that the main conclusions were robust to 16S rRNA gene copy-number correction. Correction increased the resolution of ASV-level signals but did not reveal a stronger broad taxonomic disruption. The fact that fewer higher-rank taxa remained significant after correction strengthens the interpretation that glasmacinal exposure was associated with limited ecological restructuring rather than broad antibiotic-like dysbiosis.

Table S7. Number of significantly differentially abundant taxa in the original and 16S gene-copy-number-corrected analyses. defined as |LFC| > 1 with adjusted $p < 0.05$. Δ indicates the difference between corrected and original branchesanalyses (GCN-corrected − original).

Level	BL1–1W (original)	BL1–1W (GCN)	Δ1W	BL1–2W (original)	BL1–2W (GCN)	Δ2W
**PHYLUM**	3	2	-1	4	4	0
**CLASS**	5	6	1	8	5	-3
**ORDER**	14	13	-1	13	12	-1
**FAMILY**	20	19	-1	22	16	-6
**GENUS**	42	39	-3	39	35	-4
**ASV**	79	88	9	72	108	36

In conclusion, across all analytical domains, the original and 16S GCN-corrected pipelines produced broadly concordant results. Alpha-diversity conclusions were unchanged after correction: Faith's phylogenetic diversity showed the same significant reductions at treatment time points, while Pielou's evenness remained non-significant in all pairwise contrasts. Thus, 16S gene-copy-number correction did not alter the interpretation of within-sample diversity.

Beta-diversity structure was also strongly preserved. Weighted UniFrac ordinations remained highly concordant between branches, as confirmed by Procrustes analysis ($M^2 = 0.1626$, $r = 0.9151$, Protest $p = 0.0001$). Most pairwise PERMANOVA results were unchanged, with significant baseline-to-treatment separation retained for the main treatment-associated contrasts and no evidence of increased divergence between 1W and 2W. The only discordant comparison was BL2–2W, which was close to the significance threshold in the original branch and became significant after correction despite a very similar R^2. This is best interpreted as a borderline, method-sensitive contrast rather than a qualitative change in beta-diversity structure.

Differential abundance analyses likewise supported the same overall biological interpretation. Log fold-change estimates were strongly correlated between branches across taxonomic levels, indicating that copy-number correction largely preserved the direction and relative ranking of treatment-associated abundance changes. The corrected analysis identified more significant ASVs, especially at 2W, but this did not translate into more significant higher-rank taxa. Instead, genus-, family-, order-, and class-level signals were generally similar or reduced after correction. This pattern is expected because GCN correction is applied at the ASV level and can redistribute signal among ASVs within the same higher-rank taxon, leading to partial cancellation after aggregation.

Overall, 16S gene-copy-number correction did not reveal a stronger or qualitatively different dysbiosis signal. Rather, it confirmed that the main findings are robust: glasmacinal exposure was associated with modest ecological restructuring, detectable in diversity and differential-abundance analyses, but without evidence of broad antibiotic-like disruption. The original uncorrected pipeline is therefore retained as the primary analysis, while the 16S GCN-corrected branch is presented as a sensitivity analysis supporting the robustness and conservative interpretation of the study conclusions.

Section 2: Analytical Power to Detect Antibiotic-like Dysbiosis in the Glasmacinal Microbiome Study

1. Background

Scope. This document addresses a specific methodological concern: whether the glasmacinal microbiome analysis was sufficiently sensitive to detect a large, consistent antibiotic-like dysbiosis signal. It is not intended as a head-to-head biological comparison of glasmacinal with antibiotics. The external antibiotic datasets are used only as benchmarks for the magnitude and structure of dysbiosis signals that an analysis should be able to detect.

The evidence is evaluated in two complementary steps. First, the glasmacinal dataset itself is used as an internal sensitivity check. If the study design or analytical framework were generally insensitive, it would not be expected to detect clear within-subject microbiome changes. Second, external antibiotic datasets are used as benchmarks for the magnitude and structure of antibiotic-associated dysbiosis signals. These datasets are not used for direct drug-to-drug comparison, but to show that antibiotic-associated dysbiosis, when evident, typically appears as a coordinated multi-metric signal across alpha-diversity and beta-diversity measures rather than as a subtle fluctuation at the edge of detectability.

2. Methods

Five antibiotic intervention datasets from healthy adult cohorts with available data were used as external benchmarks (Table S8). Raw sequencing data from Choo et al. (2023)[2] were processed using Trimmomatic v0.39, rarefied to 15 million reads per sample, and taxonomically profiled using MetaPhlAn 4 v4.0.6. For the remaining studies, abundance tables were obtained from published supplementary materials. Within each study, count tables were rarefied to the minimum sample depth prior to alpha-diversity analysis. All downstream analyses were performed in R v4.3.1 using the packages vegan v2.6-4, lme4 v1.1-35, lmerTest v3.1-3, and emmeans v1.10.0.

Because phylogeny-based diversity metrics, including Faith's phylogenetic diversity and weighted UniFrac, could not be calculated consistently across comparator datasets owing to differences in sequencing platforms and the absence of a unified reference phylogeny, the glasmacinal dataset was re-analyzed using the same non-phylogenetic metrics as the comparator studies. This enabled a harmonized quantitative assessment across datasets.

Alpha diversity was calculated from rarefied count tables using non-phylogenetic metrics: observed richness, Shannon entropy, Simpson's index, and Pielou's evenness. Differences between timepoints were evaluated using linear mixed-effects models with subject included as a random effect to account for the paired longitudinal design. Standardized paired effect sizes were reported as $Cohen's\ d_z$, derived from the corresponding model-based contrast statistics.

Beta diversity was assessed using Bray-Curtis dissimilarity calculated from relative abundance tables. Specifically, raw count matrices were transformed to total-sum relative abundances using *decostand* function before Bray-Curtis distances were calculated. Differences in community composition between baseline and other timepoints were tested using permutational analysis of variance (adonis2) with 9,999 permutations and subject-block-constrained permutation schemes to account for repeated

measures. The PERMANOVA R^2 statistic was used to quantify the proportion of beta-diversity variance explained by timepoint.

Across studies, microbiome perturbation magnitude was summarized using the maximum absolute Cohen's d_z for alpha diversity, maximum PERMANOVA R^2 for beta diversity, and maximum median within-subject Bray–Curtis distance from baseline. Pairwise contrasts were adjusted for multiple testing using the Benjamini–Hochberg false discovery rate procedure.

For the genus-level sensitivity analysis, we implemented a simulation-based power assessment in R using the same paired LinDA differential-abundance framework applied to the glasmacinal Baseline 1 (BL1) versus 1-week (1W) analysis. All genera included in the BL1 versus 1W LinDA analysis were evaluated, and the number of simulated taxa was set to match the number of genera retained after filtering in the real dataset, thereby preserving the approximate multiple-testing/FDR burden. For each target genus, its observed BL1 relative abundance was used as the baseline abundance, and paired baseline and treatment count data were simulated for 15 subjects at a fixed sequencing depth of 90,000 reads per sample. In each simulation, the target genus was assigned a specified true fold-change between baseline and treatment, while all remaining genera were treated as unchanged background taxa. Background genera were generated by sampling from the observed glasmacinal BL1 genus-abundance distribution, so that the simulated feature space reflected the abundance structure of the real dataset rather than an artificial uniform distribution. Each simulated dataset was analysed with LinDA using the paired mixed-effects model: $\sim timepoint\ +\ (1\ |\ subject\_id)$, and the target genus was considered detected if its FDR-adjusted p-value was <0.05. Simulations were repeated 200 times per genus and fold-change value, and power was estimated as the proportion of simulations in which the target genus was detected. Data handling and iteration were performed using standard R packages including dplyr, purrr/furrr, and tibble, with parallelisation where applicable.

Table S8. Included studies and data sources

Study	Exposure	Baseline	Compared group	Sample size (n)	Material/source
Current study	Glasmacinal 375 mg/day, 14 days	Baseline 1	Baseline 2 1-week	15-17	16S rRNA V3-V4
Sewunet et al. 2024 [3]	TBPM-PI-HBr* 600 mg q8h, 10 days	Day 1	Day 7	15	16S rRNA V3-V4; supplementary count table
Sewunet et al. 2024 [3]	amoxicillin/clavulanic acid 500/125 mg q8h, 10 days	Day 1	Day 7	14-15	16S rRNA V3-V4; supplementary count table
Palleja et al. 2018 [4]	Meropenem + gentamicin + vancomycin for 4 days	Day 1 (Dag0)	Day 8 (Dag8)	12	Shotgun metagenomics; supplementary mOTU relative abundance file.
Choo et al. 2023 [2]	Azithromycin 125 mg twice daily for 4 weeks	Baseline	Week 4 (ABx)	10	Shotgun metagenomics; raw data downloaded and processed
Choo et al. 2023 [2]	Erythromycin 400 mg twice daily for 4 weeks	Baseline	Week 4 (ABx)	10	Shotgun metagenomics; raw data downloaded and processed

* Tebipenem pivoxil hydrobromide

3. Results and discussion

3.1. Internal sensitivity of the glasmacinal alpha-diversity analysis

The glasmacinal paired analysis showed a clear separation between background within-subject variation and on-treatment change, which Cohen's d_z helps to interpret. In a paired design, d_z describes the size of the average within-person change relative to the variability of those within-person changes. In simple terms, a small d_z indicates that the observed change is similar to normal within-subject fluctuation, whereas a large d_z indicates a consistent change that is clearly larger than background variation.

As shown in Table S9, the untreated Baseline 2 vs Baseline 1 comparison showed little evidence of alpha-diversity drift, with d_z values across metrics ranging from −0.531 to 0.095. By contrast, the on-treatment comparisons showed a clear phylogenetic-richness signal. Faith's PD decreased strongly at both 1-week and 2-weeks, with $d_z = -1.745$ and $d_z = -1.545$, respectively, and FDR-adjusted P-values of $3.21 \times 10^{-8}$and 1.47×10^{-7}. This indicates that the richness reduction was large relative to within-subject variability and was robustly detected by the paired model. Therefore, the results argue against the interpretation that the analysis was globally insensitive because of sample size or methodological limitations.

The magnitude of the Faith's PD effect supports this conclusion quantitatively. In a paired comparison, $d_z$is directly related to the test statistic $\left(t = d_z \times \sqrt{n}\right)$. The Faith's PD reduction after 1 week of glasmacinal treatment ($d_z = -1.745$) therefore generated a strong statistical signal, with a t-ratio of −7.195.

The same effect size can also be used to estimate how many paired samples would be required to detect an effect of this magnitude. For a paired design, the approximate sample size was calculated using equation (1).

$$n \approx \left(\frac{t_{1-\alpha/2,df} + t_{1-\beta,df}}{|d_z|}\right)^2 \qquad (1)$$

Assuming a two-sided test with $\alpha = 0.05$, 80% power ($\beta = 0.20$), and $df = n - 1 = 14$, the relevant t-values are approximately: $t_{0.975,14} = 2.145$ and $t_{0.80,14} = 0.868$. Therefore, the number of samples can be calculated as equeation (2).

$$n \approx \left(\frac{2.145 + 0.868}{|d_z|}\right)^2 = \left(\frac{3.013}{|d_z|}\right)^2 \qquad (2)$$

Based on the observed 1-week Faith's PD effect ($d_z \approx -1.745$), only approximately 4 paired samples, meaning 4 participants with both baseline and on-treatment samples, would be required to achieve 80% power at $\alpha = 0.05$.

By contrast, the observed Pielou's evenness effect was extremely small ($d_z \approx -0.097$). Under the same paired-test framework, detecting an effect of this magnitude with 80% power at $\alpha = 0.05$ would require approximately 965 paired participants. This large sample-size estimate is not intended as a practical recruitment target, but rather illustrates that the estimated evenness change was very close to zero. In other words, the non-significant evenness result does not appear to represent a missed large perturbation; the observed effect itself was minimal. Together with the robust detection of richness and Faith's PD reductions, this supports the conclusion that the analysis had appropriate sensitivity for large within-subject alpha-diversity changes, while providing no evidence for a substantial evenness collapse.

Table S9. Internal sensitivity assessment of paired alpha-diversity analyses in the glasmacinal cohort. Each diversity metric was analysed using a linear mixed-effects model with visit as a categorical fixed effect and participant ID as a random intercept to account for repeated measurements. Estimated marginal means were used to calculate two-sided pairwise contrasts comparing Baseline 2 (BL2), week 1 (1W), and week 2 (2W) with Baseline 1 (BL1). The table reports the estimated mean difference, standard error (SE), t-ratio, paired standardized effect size (Cohen's d_z), and Benjamini–Hochberg-adjusted p-value. Negative estimates indicate lower diversity relative to BL1. Significance codes: *** adjusted p < 0.001; ns, not significant after false-discovery-rate adjustment.

Metric	Comparison	Estimate	SE	t-ratio	Cohen's d_z	Adj. P-value
Faith's PD	BL2 – BL1	0.188	0.478	0.394	0.095	0.6947 (ns)
	1W – BL1	-3.443	0.478	-7.195	-1.745	3.21×10^{-8} ***
	2W – BL1	-3.050	0.478	-6.373	-1.545	1.47×10^{-7} ***
Pielou's evenness	BL2 – BL1	-0.01636	0.00936	-1.749	-0.424	0.690 (ns)
	1W – BL1	-0.00375	0.00936	-0.401	-0.097	0.690 (ns)
	2W – BL1	-0.00482	0.00936	-0.515	-0.125	0.690 (ns)
Observed richness	BL2 – BL1	4.66	34.91	0.134	0.032	0.894 (ns)
	1W – BL1	-200.96	34.91	-5.756	-1.396	2.53×10^{-6} ***
	2W – BL1	-194.31	34.91	-5.566	-1.350	2.53×10^{-6} ***
Shannon	BL2 – BL1	-0.105	0.077	-1.364	-0.331	0.179 (ns)
	1W – BL1	-0.191	0.077	-2.492	-0.604	0.051 (ns)
	2W – BL1	-0.180	0.077	-2.353	-0.571	0.051 (ns)
Simpson	BL2 – BL1	-0.00374	0.00171	-2.191	-0.531	0.299 (ns)
	1W – BL1	-0.00164	0.00171	-0.960	-0.233	0.395 (ns)
	2W – BL1	-0.00146	0.00171	-0.857	-0.208	0.395 (ns)

3.2. Antibiotic-Associated Alpha-Diversity Dysbiosis

The comparator antibiotic datasets were used as a sensitivity benchmark for the paired alpha-diversity framework. The relevant question was whether this analytical approach could detect antibiotic-associated microbiome shifts of the type and magnitude reported in independent datasets. Across the comparator antibiotics, paired effect sizes were generally negative across multiple alpha-diversity metrics, although their magnitude varied by drug and timepoint (Table S10 and Figure S4). Several exposures, including tebipenem, Mer-Gen-Van, azithromycin, and erythromycin, showed broad reductions in observed richness, Shannon diversity, Simpson diversity, and Pielou's evenness, with multiple d_z values exceeding $|1|$ and in some cases approaching or exceeding $|2|$. Amoxicillin-clavulanate showed a more moderate profile, but its effects remained directionally negative across the alpha-diversity metrics.

This benchmark should be interpreted together with the internal glasmacinal sensitivity signal. The glasmacinal analysis itself detected large within-subject reductions in richness, with $d_z = -1.74$. Many antibiotic-associated effects in the comparator datasets were of similar or greater magnitude across multiple metrics. Therefore, had glasmacinal produced a large, coordinated antibiotic-like dysbiosis signal at the sampled treatment timepoints, the paired analytical framework would be expected to detect it. The absence of similarly large Shannon, Simpson, or Pielou evenness effects is

therefore better interpreted as evidence against a broad alpha-diversity collapse, rather than as a general failure of analytical sensitivity.

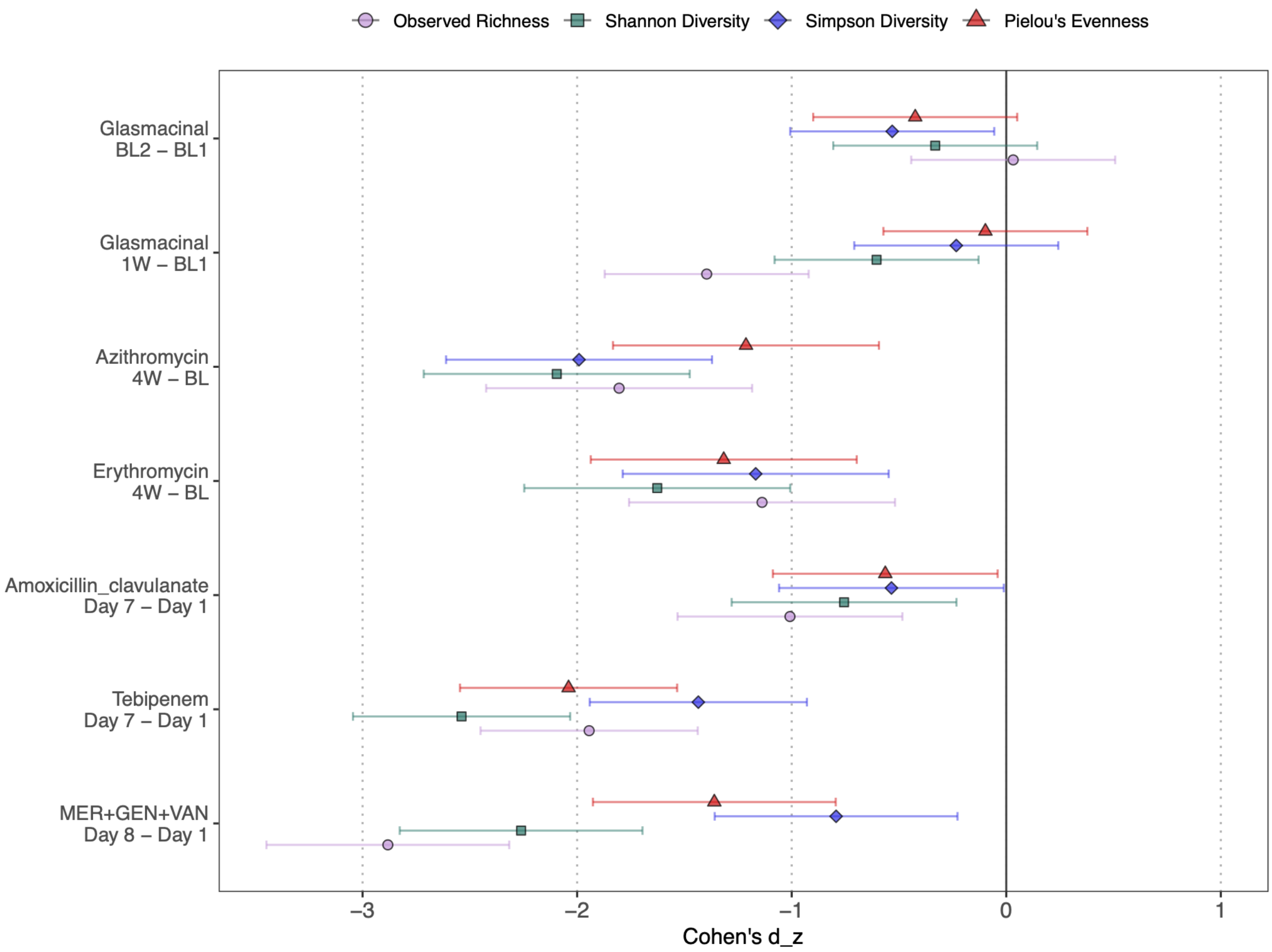

Figure S4. Paired alpha-diversity effect sizes across glasmacinal and comparator antibiotic studies. Cohen's d_z values are shown for observed richness, Shannon diversity, Simpson diversity, and Pielou's evenness for each post-baseline contrast. Points indicate the paired standardized effect size, and horizontal error bars show an approximate uncertainty range calculated as $d_z \pm (d_z/t - ratio)$. Negative values indicate reductions relative to baseline. The figure illustrates the glasmacinal profile as a large richness effect with smaller Shannon, Simpson, and evenness changes, while comparator antibiotic datasets show variable but often broader multi-metric alpha-diversity reductions.

The divergent alpha-diversity profile of glasmacinal, marked richness reduction alongside preserved evenness, Shannon, and Simpson indices, warrants specific mechanistic comment, as it differs structurally from all comparator antibiotic profiles in Table S10. In antibiotic-induced dysbiosis, evenness typically collapses because a small number of opportunistic or resistant taxa (e.g., Enterobacteriaceae, Candida, Clostridium) expand rapidly while broad commensal populations contract, producing simultaneous reductions in richness and evenness. The preservation of evenness in the glasmacinal dataset indicates that no such dominance shift occurred: the relative proportions of abundant taxa remained stable, and no opportunistic expansion was detected in the differential abundance analysis. The observed richness reduction most likely reflects a modest, transient contraction of rare low-abundance taxa rather than a restructuring of dominant community members, a pattern qualitatively distinct from antibiotic dysbiosis and more consistent with a mild, non-selective perturbation of the peripheral community. This interpretation is further supported by the modest

Bray-Curtis community structure and the absence of macrolide-characteristic taxa-level shifts in the LinDA analysis.

Table S10. Paired alpha-diversity effect sizes for glasmacinal and antibiotic benchmark datasets.

Drug	Contrast	n	***Cohen's d_z***			
			**Observed richness**	**Shannon**	**Simpson**	**Pielou's evenness**
**Glasmacinal**	1W – BL1	15 , 17	-1.396	-0.604	-0.233	-0.097
**Azithromycin**	4W – Baseline	10	-1.80	-2.09	-1.99	-1.21
**Erythromycin**	4W – Baseline	10	-1.14	-1.63	-1.17	-1.32
**Amoxicillin-clavulanate**	Day 7 – Day 1	14	-1.01	-0.76	-0.54	-0.56
**Tebipenem**	Day 7 – Day 1	15	-1.94	-2.54	-1.44	-2.04
**Meropenem + gentamicin + vancomycin**	Day 8 – Day 1	12	-2.88	-2.26	-0.79	-1.36

3.3. Beta-diversity: internal sensitivity and community-level benchmark

The Bray-Curtis PERMANOVA analysis provides another sensitivity check (Table S11, Figure S5 and Figure S6). The glasmacinal baseline-to-baseline (BL2 – BL1) comparison showed little background community drift, with $R^2 = 0.0117$ and no significant separation. In contrast, the 1-week on-treatment glasmacinal comparison (1W – BL1) was significant despite a modest effect size, with $R^2 = 0.0365$ and adjusted $p = 0.0006$. This indicates that the beta-diversity framework was able to detect a small but consistent community-level shift in the dataset.

The antibiotic benchmark datasets showed larger Bray-Curtis displacements, with R^2 values ranging from 0.0726 for amoxicillin-clavulanate to 0.2699 for the Mer-Gen-Van broad-spectrum intervention (Figure S5). These values are approximately 2- to 7-fold higher than the glasmacinal 1-week R^2, and around 6- to 23-fold higher than baseline-to-baseline drift. Thus, an analysis capable of detecting the small glasmacinal-associated community shift observed here would also be expected to detect a substantially larger antibiotic-associated community shift, had such a perturbation occurred at the sampled glasmacinal treatment timepoint.

Table S11. Pairwise PERMANOVA results for Bray–Curtis beta diversity in the glasmacinal and antibiotic benchmark datasets. For each study, the specified baseline and follow-up samples were compared using pairwise permutational multivariate analysis of variance based on Bray–Curtis dissimilarities. The table reports the pseudo-(F) statistic, (R^2), and multiple-testing-adjusted p-value for each non-directional comparison. (R^2) denotes the proportion of variation in community composition attributable to the corresponding time-point contrast. P-values were obtained from 9,999 permutations constrained within participant and adjusted using the Benjamini–Hochberg procedure. Significance codes: ** adjusted p < 0.001; * adjusted p < 0.01; ns, not significant.

Study / exposure	Contrast	F-model	R^2	Adj. P-value	Significance
**Glasmacinal**	BL2 – BL1	0.354	0.0117	1.000	ns
**Glasmacinal**	1W – BL1	1.135	0.0365	0.0006	**
**Amoxicillin-clavulanate**	Day 7 – Day 1	2.035	0.0726	0.0007	**
**Erythromycin**	4W – BL	1.603	0.0818	0.0039	*
**Tebipenem**	Day 7 – Day 1	4.137	0.1287	0.0002	**
**Azithromycin**	4W – BL	3.167	0.1496	0.0020	*
**Meropenem + gentamicin + vancomycin**	Day 8 – Day 1	8.133	0.2699	0.0049	*

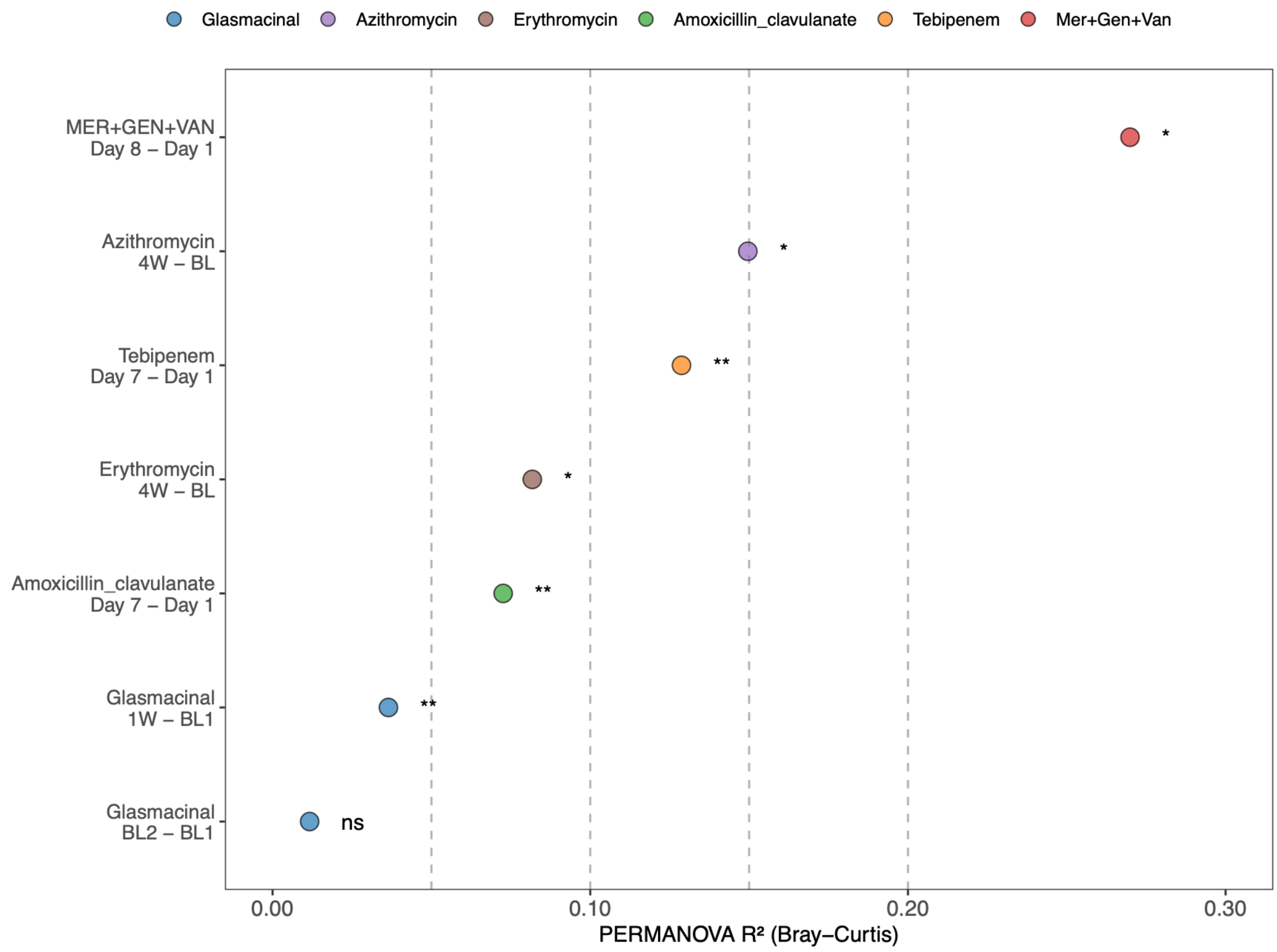

Figure S5. Bray-Curtis beta-diversity community shifts across glasmacinal and antibiotic benchmark datasets. Points show pairwise PERMANOVA R^2 values for each baseline-versus-follow-up contrast using Bray-Curtis dissimilarity. R^2 represents the proportion of community compositional variation explained by the contrast and is used here as a measure of community shift magnitude. Significance labels indicate FDR-adjusted PERMANOVA results.

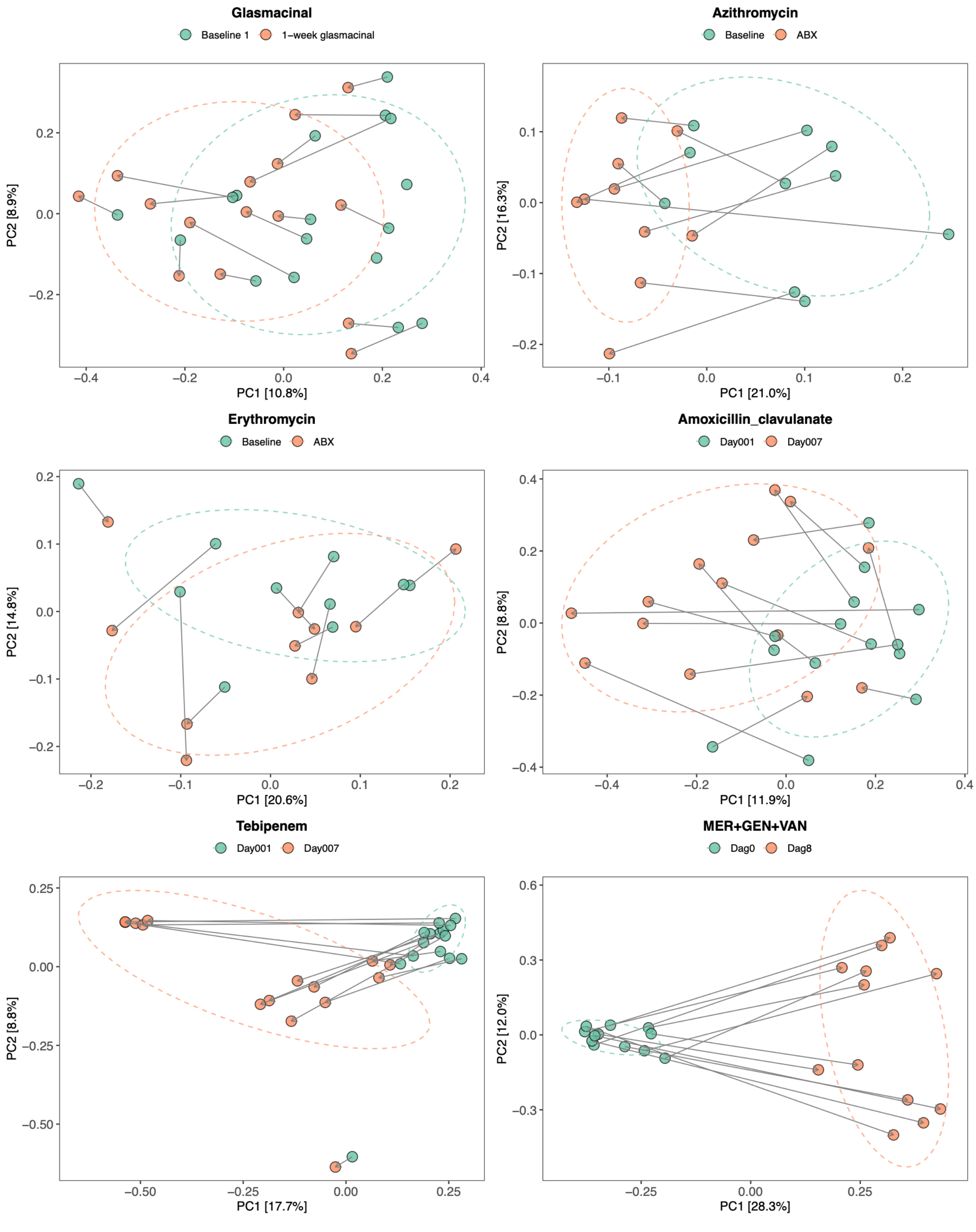

Figure S6. Bray-Curtis PCoA ordination of paired baseline and follow-up timpoint across glasmacinal and antibiotic benchmark datasets. Points represent individual samples, coloured by timepoint, and grey lines connect paired samples from the same participant between baseline and follow-up. The plot illustrates the magnitude and direction of within-subject community displacement, with glasmacinal showing a modest paired shift compared with the larger and more clearly separated community shifts observed in several antibiotic benchmark datasets.

3.4. Differential-abundance sensitivity analysis

To complement the diversity-based analyses, we performed a genus-level sensitivity analysis using the glasmacinal one-week LinDA results (90,000 sequencing depth, 200 Monte Carlo iterations). The

purpose was to address a practical question: was the differential abundance framework capable of detecting genus-level changes of biologically relevant magnitude, or could a large antibiotic-like taxonomic shift have been missed due to insufficient analytical sensitivity? This was not intended as a formal proof of absence for each non-significant genus, but as a simulation-based assessment of detectability under the glasmacinal repeated-measures design.

As shown in Figure S7, of 105 tested genera, 84 (80%) achieved ≥80% post-hoc power for their observed fold-changes. All 38 significant genera (p.adj<0.05 and |LFC|>1) were well-powered (post-hoc power = 1.00), their lack of significance reflects true biological stability. Importantly, 46 of 67 non-significant genera also fell above the 80% power threshold, indicating that their lack of significance reflects true biological stability, consistent with subject-to-subject heterogeneity, inconsistent direction of change, or low prevalence, rather than insufficient analytical sensitivity. The remaining 21 genera were underpowered, but inspection of their observed fold-changes revealed near-null effects (median |LFC| < 0.2), indicating that low power in these genera reflects an absence of biological signal rather than a limitation of the study design.

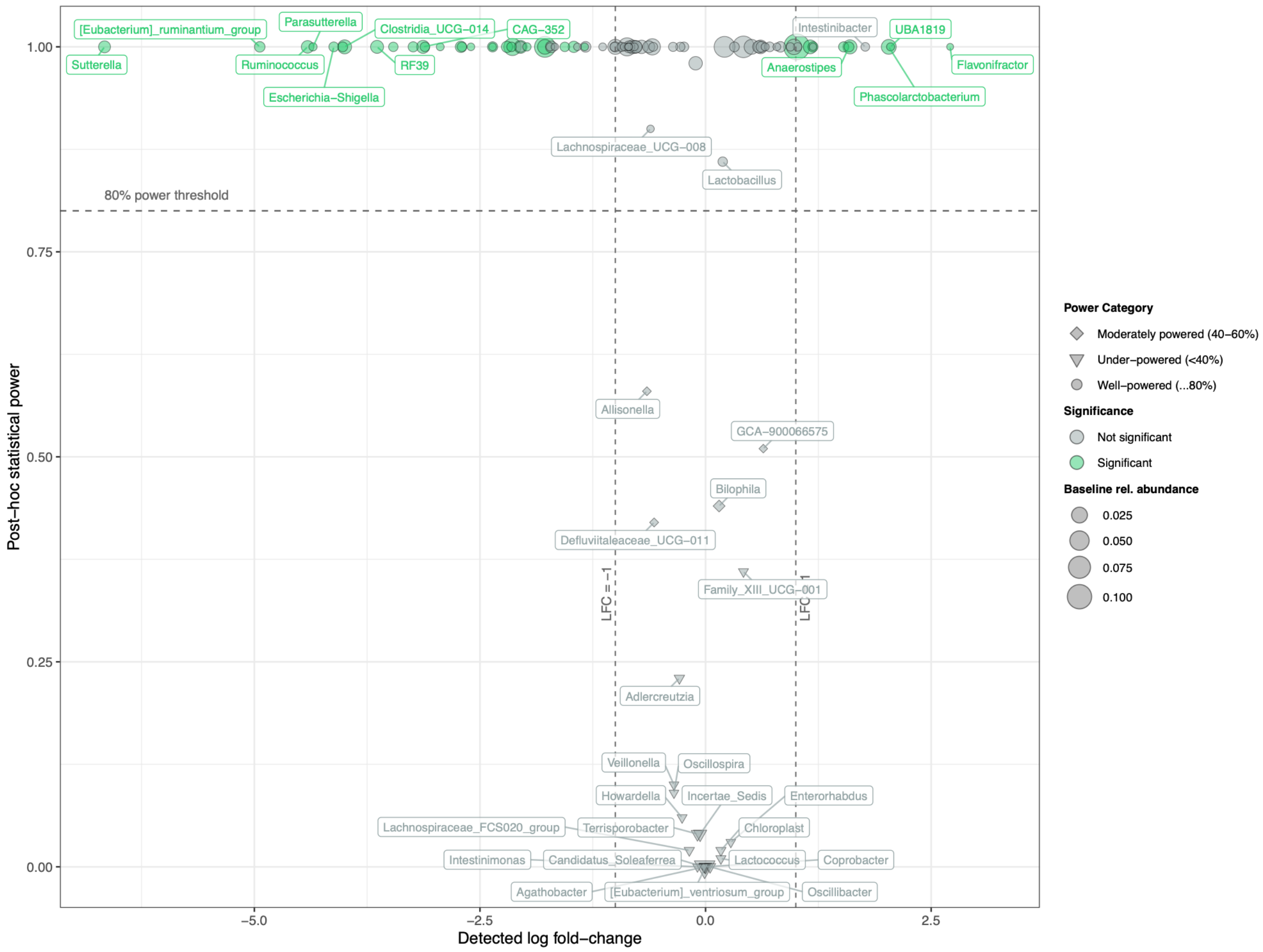

Figure S7. Genus-level LinDA sensitivity analysis for the glasmacinal 1-week comparison. The x-axis shows the observed log fold-change and the y-axis shows simulated detection probability under the glasmacinal-like paired design. Point colour indicates whether the genus was significant after FDR correction in the observed LinDA analysis, point size indicates baseline relative abundance, and point shape indicates simulated power category. The figure shows that most tested genera had high simulated detectability, supporting sensitivity of the LinDA workflow to consistent genus-level changes.

The minimum detectable fold-change at 80% power ($MDFC_{80}$) ranged from 1.1 to 2.0-fold across all genera. The majority of genera, particularly those of higher abundance, showed sensitivity to detect changes as small as 10–20% (MDFC 1.1x: n = 37 genera; MDFC 1.2x: n = 29 genera). Sensitivity was moderately reduced for rarer genera (MDFC 1.3x: n = 10; MDFC 1.5x: n = 18; MDFC 2.0x: n = 11), reflecting the expected relationship between baseline abundance and statistical power in compositional microbiome data. Notably, the genera with MDFC 2.0x were predominantly very low-abundance taxa (baseline mean relative abundance < 0.03%), for which detecting smaller fold-changes would require substantially larger sample sizes regardless of analytical framework.

The $MDFC_{80}$ estimates are particularly informative for interpreting key dysbiosis markers. *Bifidobacterium*, a canonical marker of antibiotic-induced dysbiosis, had an $MDFC_{80}$ of 1.1-fold, indicating high simulated sensitivity for this genus. In the glasmacinal data, *Bifidobacterium* was detected as significantly enriched at one week (3.03-fold increase, FDR-adjusted p = 0.0059, post-hoc power = 1.00). By contrast, macrolide and broad-spectrum antibiotic benchmark datasets consistently show large *Bifidobacterium* depletion (2–3-fold decreases)[2]. Had a comparable depletion occurred under glasmacinal at the one-week timepoint, the LinDA workflow would be expected to detect it with near-certainty. The observation of statistically significant enrichment in the opposite direction further supports the interpretation that glasmacinal does not exert macrolide-like selective pressure on this genus.

Overall, the genus-level simulation confirms that the differential abundance framework was sufficiently sensitive to detect antibiotic-like dysbiosis had it occurred. $MDFC_{80}$ values of 1.1–1.2x across the majority of genera indicate that clinically meaningful, consistent shifts of the type produced by short-course antibiotics would not have been missed. The analysis does not exclude subtle, rare, transient, or highly heterogeneous taxon-level effects, particularly among low-abundance genera with $MDFC_{80} \geq 1.5$x, but provides strong evidence against the concern that a large, consistent antibiotic-like genus-level shift would have escaped detection by the differential abundance framework.

References

1. Gao, Y. & Wu, M. Accounting for 16S rRNA copy number prediction uncertainty and its implications in bacterial diversity analyses. *ISME Communications* **3**, 59 (2023).
2. Choo, J. M. *et al.* The Impact of Long-Term Macrolide Exposure on the Gut Microbiome and Its Implications for Metabolic Control. *Microbiol Spectr* **11**, e00831-23 (2023).
3. Sewunet, T. *et al.* Effect of tebipenem pivoxil hydrobromide on the normal gut microbiota of a healthy adult population in Sweden: a randomised controlled trial. *The Lancet Microbe* **5**, e355–e365 (2024).
4. Palleja, A. *et al.* Recovery of gut microbiota of healthy adults following antibiotic exposure. *Nat Microbiol* **3**, 1255–1265 (2018).
