# Supplementary material for: Impact of the anti-inflammatory macrolide glasmacinal on the gut microbiota of healthy adults: an open-label trial
Source: Nat Commun. 2026 Aug 20;17:8728. doi: 10.1038/s41467-026-76867-9 (PMC13493899; doi:10.1038/s41467-026-76867-9)
Supplement: Supplementary file 2 [file 41467_2026_76867_MOESM2_ESM.pdf]

DESCRIPTION OF ADDITIONAL SUPPLEMENTARY FILES

File Name: Supplementary Data 1
Description: Contains the phylogenetic tree of the analysed amplicon sequence variants in Newick format. ASV tip identifiers correspond to those in the feature tables. The tree was used to calculate Faith's phylogenetic diversity and weighted UniFrac distances and for phylogenetic dispersion analysis and tree visualisation.

File Name: Supplementary Data (Source _Data_Glasmacinal.xlsx)
Description: Supplementary Data 2–14:

Supplementary Data 2: Per-sample Faith's phylogenetic diversity and Pielou's evenness values, with sample identifiers, participant identifiers, and study time points.

Supplementary Data 3: Pairwise weighted UniFrac distance matrix. Rows and columns represent samples; the matrix is symmetric with a zero diagonal.

Supplementary Data 4: ASV-level read counts, with sample identifiers, ASV identifiers, taxonomic assignments, and classifier confidence scores.

Supplementary Data 5: ANCOM-BC2 differential-abundance results at the ASV, genus, family, order, class, and phylum levels, including log2 fold changes, standard errors, adjusted P values, significance indicators, and taxonomic classifications.

Supplementary Data 6: LinDA differential-abundance results at the ASV, genus, family, order, class, and phylum levels, including mean abundance, log2 fold change, standard error, test statistic, unadjusted and adjusted P values, degrees of freedom, and taxonomic classification.

Supplementary Data 7: Comparison of LinDA and ANCOM-BC2 results, including effect estimates, adjusted P values, ANCOM-BC2 pseudo-count sensitivity status, method-specific detection, and agreement in the direction of effect.

Supplementary Data 8: Sample metadata, including sample identifiers, participant identifiers, and study time points.

Supplementary Data 9: Per-sample Deblur sequence-processing statistics, including raw, dereplicated, denoised, chimeric, artifact-filtered, and reference-filtered reads.

Supplementary Data 10: Per-sample Faith's phylogenetic diversity and Pielou's evenness values after predicted 16S rRNA gene copy-number correction, with sample identifiers, participant identifiers, and study time points.

Supplementary Data 11: Pairwise weighted UniFrac distance matrix calculated after 16S rRNA gene copy-number correction. Rows and columns represent samples; the matrix is symmetric with a zero diagonal.

Supplementary Data 12: ASV-level abundance values after predicted 16S rRNA gene copy-number correction, with sample identifiers, ASV identifiers, taxonomic assignments, and classifier confidence scores.

Supplementary Data 13: LinDA differential-abundance results after 16S rRNA gene copy-number correction at the ASV, genus, family, order, class, and phylum levels, including mean abundance, log2 fold change, standard error, test statistic, unadjusted and adjusted P values, degrees of freedom, and taxonomic classification.

Supplementary Data 14: Participant-level quantitative culture data across study time points, target taxa, and culture media, including anonymised participant identifiers and log10-transformed colony-forming-unit measurements.
