# Supplementary material for: Impact of the anti-inflammatory macrolide glasmacinal on the gut microbiota of healthy adults: an open-label trial
Source: Nat Commun. 2026 Aug 20;17:8728. doi: 10.1038/s41467-026-76867-9 (PMC13493899; doi:10.1038/s41467-026-76867-9)
Supplement: Supplementary file 4 [file 41467_2026_76867_MOESM6_ESM.pdf]

nature portfolio

Peer Review File

Impact of the anti-inflammatory macrolide glasmacinal on the gut microbiota of healthy adults: an open-label trial

Corresponding Author: Professor Christian Giske

This file contains all reviewer reports in order by version, followed by all author rebuttals in order by version.

Version 0:

Reviewer comments:

Reviewer #1

(Remarks to the Author)
Sewunet and colleagues present microbiota data obtained from a clinical trial investigating potential drug–drug interactions between glasmacinal (EP395), a macrolide used for COPD, and midazolam and digoxin. The study includes 19 healthy adults (14 completing the trial) with longitudinal fecal sampling at baseline, one week after a single dose of midazolam/digoxin, and one and two weeks after EP395 treatment. Notably, the study lacks a placebo or untreated control group to assess normal temporal variation of the gut microbiota.

A notable strength of the study is the integration of in silico microbiome analyses with ex vivo experimental assays, The authors performed 16S rRNA gene sequencing and analysed microbial richness, community structure, and relative abundance across trial stages. In addition, the authors assessed ex vivo growth inhibition of selected bacterial taxa (by quantifying colony-forming units) and explored the selection of azithromycin resistance across time points. The authors also extended resistance profiling for selected Enterobacteriales and Bacteroides isolates.

Overall, this is an interesting and potentially valuable dataset, as drug–microbiome interaction studies remain limited despite their clear clinical relevance. However, the current manuscript has several important limitations, some of which are acknowledged by the authors. These include the limited depth of microbiome analyses, insufficient justification and description of the experimental design, the small sample size, and the absence of a control or placebo group. Furthermore, the use of 16S rRNA gene sequencing restricts interpretation; shotgun metagenomics would have enabled functional and resistome analyses and strengthened the link between observational microbiome data and the ex vivo experiments.

Acknowledging that these limitations are difficult to overcome in a rebuttal, I have the following suggestions to improve the current manuscript:

Main suggestions and major comments

1. Diversity metrics and beta-diversity analyses section

The authors limit their analyses to Faith’s phylogenetic diversity for alpha diversity and weighted UniFrac for beta diversity. Have the authors considered other commonly used metrics, such as Shannon diversity for alpha diversity and Bray–Curtis or Aitchison distance for beta diversity?

The authors should clarify how differences between visits were tested in the PERMANOVA analysis. Was “visit” modeled as a single multi-level factor, or were pairwise comparisons performed between time points?

While rarefaction was applied for richness analyses to account for sequencing depth, it is unclear whether the rarefied feature table was also used for beta-diversity and differential abundance analyses. If not, the authors should clarify how sequencing depth and multiple testing were accounted for in these analyses.

2. Microbial community composition: differential abundance analysis section

Although changes in several ASVs are reported, the manuscript does not clearly describe which taxa are differentially abundant. A more detailed description of the results is needed. In addition, the authors mention that differential abundance analyses were performed at different taxonomic levels, but these results are not discussed.

3. Quantitative culture and resistance selection experiments section

The rationale for selecting specific taxa of interest in the ex vivo experiments (Enterococcus, Bacteroides, Lactobacillus, Clostridioides difficile, and Candida) is insufficiently explained. While these are described as “clinically important taxa,” the justification beyond C. difficile is unclear, as is the overall objective of these experiments within the context of the study.

The relevance of these experiments would have been clearer if the taxa of interest were selected based on findings from the differential abundance analyses rather than a priori selection.
Regarding resistance analyses, the goal of exploratory testing in Enterobacteriales and Bacteroides is not clearly articulated. A broader screening across additional species or strains would have strengthened the conclusions.

Finally, it is unclear whether any technical replicates were included in the experiments. This should be clarified.

4. Discussion section

The first three paragraphs of the Discussion primarily summarize the results and would be more appropriate in the Results section. The authors could expand their discussion with a more thorough comparison with existing studies on other macrolides and their effects on the gut microbiota.

In line 165, the authors report that Bifidobacterium longum decreases after azithromycin intake but increases following EP395 treatment. Speculation on possible mechanisms underlying this differential response would strengthen the discussion.

Finally, the authors conclude that EP395 treatment does not induce gut dysbiosis. This claim is not sufficiently substantiated. First, “dysbiosis” is not defined, and second, dysbiosis is not explicitly assessed in the analyses.

5, General comments on sequencing data processing

The authors employ a well-established pipeline for 16S rRNA gene sequencing analysis. However, important steps such as contaminant removal and 16S gene copy-number correction do not appear to have been included. The authors should consider tools such as SCRuB for decontamination and RasperGade16S for copy-number correction.

6.General comments on statistical analysis

Critical information regarding data filtering (e.g., removal of low-abundance or low-prevalence taxa) and data transformation is missing from both the Methods and Results sections. Supplementary Table 6 suggests that no filtering was applied, as 4,645 taxa were tested. Given the sparsity of microbiome data, this likely results in a high false-positive rate. In fact, q-values, which I interpret as multiple-testing–corrected p-values, appear mostly to be 0 or 1, and some differentially abundant features (e.g., g__Izemoplasmatales; s__uncultured_organism) are present in only 10 out of 63 samples. If this is the case, I would recommend applying a prevalence filter (e.g., removing taxa present in fewer than 30–40% of samples).

Given the longitudinal nature of the study, statistical methods should account for the non-independence of samples from the same participant. The methods described in the manuscript and GitHub repository suggest this may not have been done. The authors should clarify this point. If not accounted for, approaches such as paired tests, linear mixed-effects models, or block permutations for beta-diversity analyses should be considered.

Could the authors clarify whether ANCOM-BC or ANCOM-BC2 was used, and whether relevant covariates (e.g., age, sex, intestinal transit time) were considered in the models?

Minor comments

- The term “genotypic” is used where “in silico” would be more appropriate.
- Line 142: The statement “loss of phylogenetic diversity without a significant shift in relative abundances of the remaining taxa” requires clarification. What is meant by “remaining taxa,” and how can diversity decrease without affecting relative abundances?
- The claim that treatment “did not impact the essential anaerobic components” is unclear. How was this determined?
- The authors state that “normalised counts were exported from the ANCOM-BC output for downstream visualisation.” Please clarify how counts were normalized.
- The terms “16S rDNA” and “metagenomic” are used inconsistently throughout the manuscript and should be replaced with “16S rRNA gene sequencing.”
- In the “Summary of trial population and sample collection” section, additional cohort details would be helpful. A schematic figure summarizing the study design and sampling scheme is strongly recommended.
- The statement “The current literature on gut health focuses predominately on composition rather than diversity alone”

should be reconsidered or supported with references, as numerous studies have examined microbiome diversity in relation to health and disease.
• Regarding the conclusion that “EP395 did not impose selection pressure over the 2 weeks of treatment,” while two weeks may be insufficient to detect community-level changes, strain- or subspecies-level selection cannot be excluded based on the current analyses.
• The overview of QIIME 2 preprocessing is currently presented in the Results section and could be moved to the Methods section.

Reviewer #2

(Remarks to the Author)
Overall, the study is interesting and hypothesis generating, but not confirmatory or broad reaching. It is a very small study for making solid microbiome composition and diversity conclusions, and it represents a small subset of the population/treatment relevant to the hypothesis. There are some methodological questions that potentially impact the interpretation and conclusions. Some of the conclusions are over- or misstated and should be clarified.

First, why did the authors choose to conduct 16S sequencing? This seems rather inadequate in this day and age. An average of 135K reads per sample is a rather low sequencing depth and probably insufficient for meaningful differential features analyses. There is no discussion of how read depths are normalized across samples, despite there being up to 100% differences in read depths from the samples with the fewest to the highest reads. There's also no discussion of how rare ASVs vs zeros are handled. This is important, especially when conducting differential features analyses. The set of samples could have been analyzed by shallow or deep shotgun sequencing with minimal additonal cost. Overall, the microbiome methods are very average, somewhat outdated, and not described well (eg extraction methods, library prep, rarefaction...).

The size of the study is simply too small for the hypothesis tests and differential features analyses being conducted with the microbiome data. Too many studies have made bold conclusions on small sample sizes, with some conclusions flawed in such analysis and leading to significant medical conclusions that may not be well founded. In this case, the premise is that this study is sufficient to suggest that the novel macrolide can be useful for chronic treatment of COPD because no "dysbiosis" is caused, because the "core anaerobic taxa" remain intact, and because no resistance is generated. These are pretty bold statements that carry medical weight, but the study is not appropriately powered to make such bold statements with their implications.

With respect to diversity analyses, there was a difference in richness from before to after macrolide treatment. Baseline 1 and 1 week were significantly different compositions based on beta diversity analyses. Therefore, there are definitely differences as a result of macrolide administration, so the statement that "gut dysbiosis" was not induced becomes subjective as to what defines dysbiosis, and this is a central problem with the paper. Similarly, taken at face value despite the methodological questions, Figure 3 appears to show distinct differences from pre- to post-macrolide treatments, with several taxa often attributed to healthy functions (eg Clostridia, Prevotella, Akkermansia…), and there appears to be a distinct "overgrowth" of Bifidobacterium. These could all be characterized as "dysbiosis" from one subjective point of view.

The paper also states that "core anaerobic taxa were preserved" which is subjective ("core") and not supported. The selective plating are elegant but do not include a general anaerobic agar (eg CDC blood agar…) or any agar for Clostridia other then C. difficile. Perhaps egg yolk agar after ethanol treatment or the like to assess whether spore-form Firmicutes are altered, which are a significant portion of the "core anaerobic taxa." The sequencing data do not assess overall Clostridia, and the discussion only mentioned Bacterodetes as core anaerobic taxa, which in fact many Clostidia genera are found in most healthy cohorts and associated with many "core, healthy" functions.

The selective plating with antibiotics does suggest against emergence of phenotypic resistance to the antibiotics tested. However, it is only assessing the impact of a single treatment with the macrolide. It is possible that some phenotypic resistance could emerge after chronic use of the macrolide, which is a hypothesis set forth in the abstract. It is also noted that the selective plating method could miss the enrichment of antimicrobial resistance genes that could manifest as phenotypic resistance after more exposure to the macrolide. This is all acknowledged in the limitations section of the discussion, but I think it is a substantive limitation that precludes the scope of conclusions made in the article.

Overall, I suggest this is not acceptable for publication. If the sample size were more appropriate for the research questions, and if the methods were better described and applied, then a revision could address the challenges of imprecision with terminology ("dysbiosis", "core anaerobic taxa"). However, I do not think those correct the potential for conclusions and implications that outstrip the ability of the analyses.

Version 1:

Reviewer comments:

Reviewer #1

(Remarks to the Author)
I would like to thank the authors for their efforts in revising the manuscript and for providing additional analyses. While the revisions have substantially improved the work, several concerns remain. In some instances, the authors provided purely textual responses to methodological queries—such as the decision to omit decontamination or copy-number correction—without data demonstrating that these steps would not have affected the results.

Furthermore, core limitations in the study design and the authors' subsequent justifications still constrain the ability to draw any robust conclusions, which ultimately impacts the relevance of the work. Specifically, the following points require further attention:

1. The claim that the sample size is "sufficient for a preliminary assessment" lacks support. The authors should provide a power analysis, perhaps using expected effect sizes from known antibiotic-induced dysbiosis, to justify the cohort size.

2. The discrepancy between ANCOM-BC and LinDA results raises concerns. Rather than selecting one method for the main text, the authors should transparently present both, highlighting areas of agreement and disagreement (e.g., directionality). If ANCOM-BC2 is indeed more conservative, this may actually be a methodological strength given the small sample size.

3. Several interpretations of differential abundance appear to rely on visual inspection rather than formal testing. For instance, the claim that drug effects are distributed across the taxonomic tree should be supported by an enrichment analysis. Additionally, taxa trajectories should include individual data points rather than just group-level visualizations.

4. In the experimental section, the rationale for the selected bacterial taxa remains weak. The claim that they are "associated with human health" is overly broad and applies to many omitted taxa (e.g., F. prausnitzii or Bifidobacterium). Furthermore, citing "technical issues in culturing" as a selection criterion is only valid if it refers to specific taxa identified in the in silico analysis. It remains unclear what the specific added value of this targeted screening is in its current form.

Reviewer #2

(Remarks to the Author)
Overall, I appreciate the changes made to the manuscript, which are primarily textual rather than analytical. I still feel the cohort is small and hypothesis generating, rather than conclusive, yet the text still appears aimed at conclusions. A few detailed points.

- I still view the statement that glasmacinal induced changes "…without clinically meaningful dysbiosis" is an overstated of conclusions. There are changes, they appear relatively modest, but there is no assessment of clinical meaningfulness for those changes.
- Lines 142-145: authors state findings that the "treatment-associated microbiome shift was not driven by a single dominant taxon but instead reflected coordinated increases and decreases among taxa with distinct ecological roles" but they do not discuss or address ecological roles of the taxa that changed via either functional genomics herein or literature assessment of the changing taxa. Therefore, I still find this conclusion to be somewhat overstated.
- While not comprehensive, the phenotypic culturing is probably a strength of this manuscript. The data rather clearly indicate that several taxa do not change (Lactobacillus, Bacteroides…whereas Enterobacterales clearly is decreased by glasmacinal. The notable methodological weakness in this area is the lack of definitive characterization of isolated and quantified colonies, even by gram stain and microscopic morphology. This is a rather basic technique that would provide strength to the conclusions by providing greater confidence that the measured colonies represent the taxa they are claimed to. This is not a concern with the chromagar plates due to their demonstrated selectivity and confirmatory chromagenic observation. However, BKV and Rogosa agar are well known to have breakthrough of non-Bacteroidetes and non-Lactobacilli, respectively.
- My opinion is that the conclusions are still overstated compared to the strength of the analysis. For example "glasmacinal induced limited ecological perturbations without the defining hallmarks of antibiotic-like or clinically concerning dysbiosis." These appear aimed at making strong clinically relevant conclusions that just aren't supported the analyses.
- Line 200 discussion of Akkermansia reduction being due to immunomodulatory properties should be elaborated. Are there references? This seems to be based on the conclusion that glasmacinal has no antimicrobial properties, but the dramatic decreases in phenotypic recovery of Enterobacterales suggests otherwise. It may be that glasmacinal has MIC > 64 ug/mL in vitro but that the concentrations exceed this in the colon such that antimicrobial activity can be observed. There are very clear precedents for this, such as oral vancomycin inhibition of Bacterodia in C. difficile patients despite in vitro MICs being very high. All of this is to suggest that a claim of an immunomodulatory effect on Akkermansia should be better supported.
- Similarly, I think the statement "…anti-inflammatory and immunomodulatory effects of glasmacinal might have promoted stability of anaerobic taxa…" is quite overstated. It would be better to just state the facts--glasmacinal did not appear to dramatically disrupt or reduce anaerobic taxa in this limited cohort. If there are proposed anti-inflammatory or immunomodulatory contributions to this, clearly state that these are speculations based on specific literature citations.
- Line 241, the statement "…the 2-week treatment period in this trial was likely sufficient for the detection of resistance" is a vast speculation. The invocation of M. abscessis resistance development does not teach about Enterobacterales resistance potential. These are rather distance cousins whose propensity to develop resistance may be quite divergent and highly

dependent on how critical is the pathway wherein resistance develops. Perhaps in vitro assessment of the potential to evolve resistance vs number of passages would be supportive of such a statement, but there is no data to support that here.

Version 2:

Reviewer comments:

Reviewer #1

(Remarks to the Author)
I would like to thank the authors for their thorough revisions. They have successfully addressed the majority of my concerns, provided helpful supplementary analyses, and clarified several methodological choices. I still believe it is important to present and discuss the results using ANCOM-BC2. As previously noted—and acknowledged by the authors—a comparison with ANCOM-BC (v1) is not informative here, given that the method does not properly handle repeated measurements.

Reviewer #2

(Remarks to the Author)
I appreciate the additional analyses, explanations, and qualifications that the authors added to the manuscript. While this does enhance the precision and accuracy of the study, the overall limitations of the study remain--the cohort is of limited size and scope to make significant conclusions of new import that are aligned with the scope and level of Nature Communications. I do not have specific modifications to recommend to the authors, and I defer to the editors as to whether the limited-strength conclusions are aligned with the impact of the journal.

Open Access This Peer Review File is licensed under a Creative Commons Attribution 4.0 International License, which permits use, sharing, adaptation, distribution and reproduction in any medium or format, as long as you give appropriate credit to the original author(s) and the source, provide a link to the Creative Commons license, and indicate if changes were made.
In cases where reviewers are anonymous, credit should be given to 'Anonymous Referee' and the source.
The images or other third party material in this Peer Review File are included in the article's Creative Commons license, unless indicated otherwise in a credit line to the material. If material is not included in the article's Creative Commons license and your intended use is not permitted by statutory regulation or exceeds the permitted use, you will need to obtain permission directly from the copyright holder.
To view a copy of this license, visit https://creativecommons.org/licenses/by/4.0/

Response to reviewers' comments

Reviewer #1 (Remarks to the Author):

Sewunet and colleagues present microbiota data obtained from a clinical trial investigating potential drug–drug interactions between glasmacinal (EP395), a macrolide used for COPD, and midazolam and digoxin. The study includes 19 healthy adults (14 completing the trial) with longitudinal fecal sampling at baseline, one week after a single dose of midazolam/digoxin, and one and two weeks after EP395 treatment. Notably, the study lacks a placebo or untreated control group to assess normal temporal variation of the gut microbiota.

A notable strength of the study is the integration of in silico microbiome analyses with ex vivo experimental assays, The authors performed 16S rRNA gene sequencing and analysed microbial richness, community structure, and relative abundance across trial stages. In addition, the authors assessed ex vivo growth inhibition of selected bacterial taxa (by quantifying colony-forming units) and explored the selection of azithromycin resistance across time points. The authors also extended resistance profiling for selected Enterobacteriales and Bacteroides isolates.

Overall, this is an interesting and potentially valuable dataset, as drug–microbiome interaction studies remain limited despite their clear clinical relevance. However, the current manuscript has several important limitations, some of which are acknowledged by the authors. These include the limited depth of microbiome analyses, insufficient justification and description of the experimental design, the small sample size, and the absence of a control or placebo group. Furthermore, the use of 16S rRNA gene sequencing restricts interpretation; shotgun metagenomics would have enabled functional and resistome analyses and strengthened the link between observational microbiome data and the ex vivo experiments.

Response: *We thank the reviewer for the careful evaluation of our manuscript and for the constructive and thoughtful feedback provided. We appreciate the reviewer's recognition of the potential value of this dataset and their interest in the integration of longitudinal microbiome profiling with ex vivo experimental assays to investigate drug-microbiome interactions. The reviewer raises several important points regarding experimental justification, interpretation of the microbiome data, and opportunities to strengthen the clarity and contextualization of the findings. Below, we address each of the reviewer's comments and suggestions in detail.*

Lack of Placebo Control

We agree with the reviewer that inclusion of a placebo arm is often helpful to assess the temporal variability. However, the primary aim was not to detect subtle microbiome perturbations but to identify major disruptions akin to those seen with traditional antibiotics (e.g., broad reductions in diversity or selective enrichment of resistant taxa). To address this question efficiently within the constraints of an early-phase clinical study, a single-arm longitudinal design focusing on short-term, within-subject changes was employed.

To partially mitigate the lack of a placebo arm and account for temporal variability, we incorporated two pre-treatment baseline measurements (baseline-1 and baseline-2). This design enabled assessment of intra-individual microbiome stability prior to drug exposure and allowed each participant to serve as their own control. For instance, no significant differences were observed between the two baselines across alpha diversity (Faith's PD richness and evenness), beta diversity (weighted UniFrac), and taxa abundances, indicating minimal natural fluctuation in our cohort over the pre-treatment period. This intra-subject control strengthens confidence that post-administration changes, such as the significant reduction in richness at 1-week ($p < 0.05$ vs. baseline-1) and shifts in beta diversity, are attributable to glasmacinal rather than time alone.

We have acknowledged the limitation of lack of placebo control in the Discussion section of the revised manuscript in lines263-266.

Sample Size

While larger cohorts are desirable for detecting subtle microbiome shifts, we believe the current design and conservative interpretation align well with the exploratory nature of this assessment: to evaluate whether glasmacinal induces overt "antibiotic-like" dysbiosis. As an exploratory objective within a drug–drug interaction (DDI) study, the sample size aligns with regulatory expectations for such trials, which typically enrol 12–20 participants[1,2] Accordingly, 19 subjects were enrolled, 17 were dosed with glasmacinal, and 15 provided complete longitudinal faecal sampling for microbiome analysis. Within this framework, the longitudinal within-subject design, incorporating dual pre-treatment baselines and paired statistical testing (linDA and pairwise Adonis), provides adequate sensitivity to detect large-effect, clinically meaningful disruptions in community structure despite the modest cohort size. This design and conservative interpretation are consistent with early-phase exploratory microbiome studies, where the purpose is to identify signals rather than to precisely quantify small or taxa-specific fluctuations.

Our conclusions are intentionally conservative, and data-driven. We report transient reductions in phylogenetic richness and beta-diversity shifts after glasmacinal treatment, along with taxa-specific changes, but note that these effects are modest and lack key features of antibiotic-like dysbiosis, including expansion of resistant taxa, sustained loss of anaerobes, or evidence of cross-resistance to azithromycin. The absence of such pathological signatures, rather than the presence of subtle and reversible shifts, forms the primary biological and clinical take home message.

Moreover, even with a much larger cohort, microbiome responses remain highly individual and context-dependent (diet, geography, baseline community structure), and results would still require cautious generalization. Here, the lack of severe or persistent dysbiosis in this cohort provides reassuring preliminary evidence supporting the non-antimicrobial profile of glasmacinal, sufficient to inform progression to larger patient studies while appropriately caveated.

In the Methods (Trial design) section of the revised manuscript, we have added further justification of the sample size in the context of the microbiology objective, as follows:

"The sample size was considered sufficient for a preliminary assessment of glasmacinal to induce antibiotic-like dysbiosis, including expansion of resistant taxa, sustained loss of anaerobes, or evidence of cross-resistance to azithromycin, but was not sufficient to detect subtle microbiome shifts."

Restrictions of 16S rRNA Approach

We fully agree that in many microbiome studies, particularly those exploring mechanisms or subtle functional shifts, shotgun metagenomics is the preferred approach due to its richer taxonomic resolution and access to gene-level information. However, in the specific context of this study, we believe the choice of 16S rRNA gene sequencing (V3-V4 region) was appropriate for the objective: to assess whether glasmacinal induces antibiotic-like perturbations of the gut microbiome, such as broad reductions in microbial diversity, major shifts in community structure, or expansion of taxa typically associated with antimicrobial selection. Detecting such global ecological effects does not require species- or strain-level resolution, and identifying changes at finer taxonomic levels (for example, within the genus Sutterella) would not substantially change the biological interpretation or conclusions of the study. Importantly, questions related to antimicrobial resistance were addressed more directly through targeted quantitative culture-based assays (CFU quantification and azithromycin selection experiments). These approaches are better suited to detecting functional resistance emergence in the absence of strong selection pressure and provide a more direct link to clinically relevant outcomes than gene-centric inference alone. When assessing the resistome based on shotgun sequencing, there will always be a predominance of anaerobic resistance genes of unknown clinical significance, and we argue that the approach we have used herein will be far more informative regarding emergence of resistance in bacterial species acting as human pathogens.

The Experimental Design

We have substantially revised and clarified multiple sections of the manuscript to more clearly articulate the study rationale, design choices, and analytical strategy. Specifically, we generated a new figure about the trial design and faecal sample collection time points. This new figure has been added to the revised manuscript (Figure 1).

We would also like to point out that whilst glasmacinal is a macrolide (i.e. contains a large macrocyclic lactone ring), it is also the first-in-class drug of a new class of non-antibacterial macrolide agents with anti-inflammatory activity[3]. We have provided further detail on the structure of glasmacinal and how it relates to azithromycin in the Introduction (lines 56-61).

Acknowledging that these limitations are difficult to overcome in a rebuttal, I have the following suggestions to improve the current manuscript:

Main suggestions and major comments

1. **Diversity metrics and beta-diversity analyses section**

1.1. The authors limit their analyses to Faith's phylogenetic diversity for alpha diversity and weighted UniFrac for beta diversity. Have the authors considered other commonly used metrics, such as Shannon diversity for alpha diversity and Bray–Curtis or Aitchison distance for beta diversity?

Response: *We computed standard alpha- and beta-diversity metrics implemented in QIIME2, including Faith's phylogenetic diversity (PD), Shannon index, weighted UniFrac, and Bray-Curtis distances, all part of default core-metrics-phylogenetic QIIME 2 plugins.*

For alpha diversity, we prioritized Faith's PD as the primary richness metric and reported Pielou's evenness separately. Faith's PD quantifies the total phylogenetic breadth of a community and is particularly sensitive to the loss of evolutionarily distinct lineages, which is a hallmark of antibiotic-induced dysbiosis. In contrast, the Shannon index conflates richness and evenness, which can obscure whether observed changes are driven by loss of taxa versus redistribution of abundances. This separation allowed clearer interpretation of our results, namely a transient reduction in phylogenetic richness without a concomitant loss of evenness, consistent with a mild and non-antibiotic-like perturbation.

For beta-diversity, weighted UniFrac was selected because it incorporates both relative abundance and phylogenetic relatedness, enabling detection of community shifts that are structured across related lineages. Bray-Curtis dissimilarity, while widely used, does not incorporate phylogenetic information and may therefore be less sensitive to coordinated changes among related taxa. Given that our aim was to identify biologically meaningful community restructuring rather than purely compositional turnover, weighted UniFrac was considered the most appropriate primary metric.

Regarding Aitchison distance, we acknowledge its increasing use for compositional microbiome data due to its foundation in log-ratio transformations. However, Aitchison distance does not incorporate phylogenetic relationships. We think weighted UniFrac can capture the key aspects of community change relevant to our biological question, and we therefore did not prioritize Aitchison-based analyses.

In the Methods section (Statistics and bioinformatics analyses), we have added our justification for the use of Faith's PD and Pielou's evenness as the alpha diversity metrics, and weighted UniFrac for beta-diversity (Revised manuscript lines 478-491).

1.2. The authors should clarify how differences between visits were tested in the PERMANOVA analysis. Was "visit" modeled as a single multi-level factor, or were pairwise comparisons performed between time points?

Response: *Differences in beta-diversity between visits were evaluated using a pairwise PERMANOVA approach implemented in R (pairwise.adonis2). For each pair of visits, the script subsets the distance matrix (weighted UniFrac or Bray-Curtis) to only include samples from the two visits, then runs adonis2 with subject ID as a stratification factor to account for repeated measures. The resulting F-statistic, R^2 (effect size), and p-value are recorded for each pair, and p-values are adjusted for multiple testing using FDR correction.*

We have added the above information to the Methods section (Statistics and bioinformatics analyses from line 484-491).

1.3. While rarefaction was applied for richness analyses to account for sequencing depth, it is unclear whether the rarefied feature table was also used for beta-diversity and differential abundance analyses. If not, the authors should clarify how sequencing depth and multiple testing were accounted for in these analyses.

Response: *For alpha- and beta-diversity analyses, rarefaction was applied to account for differences in sequencing depth, as required for metrics such as Faith's phylogenetic diversity. The sampling depth was set to 74,470 reads (i.e., --p-sampling-depth 74470), which allowed retention of all 62 samples while ensuring equal sampling effort across time points.*

In contrast, differential abundance analyses were performed on unrarefied count data. In the current revision of the manuscript, we used LinDA, which explicitly models and corrects for biases arising from unequal sequencing depth and compositionality through log-linear regression with bias correction[4]. This approach avoids the loss of information associated with rarefaction and is specifically designed for identifying differentially abundant taxa while appropriately controlling for multiple testing.

We have clarified these points in the Methods section by explicitly stating which analyses were conducted on rarefied versus unrarefied data and how sequencing depth and multiple testing were addressed in each case (Revised manuscript line 499-508).

1.4. Microbial community composition: differential abundance analysis section

Although changes in several ASVs are reported, the manuscript does not clearly describe which taxa are differentially abundant. A more detailed description of the results is needed. In addition, the authors mention that differential abundance analyses were performed at different taxonomic levels, but these results are not discussed.

Response: *The text has been updated in the revised manuscript lines 118-144.*

2. Quantitative culture and resistance selection experiments section

2.1. The rationale for selecting specific taxa of interest in the ex vivo experiments (Enterococcus, Bacteroides, Lactobacillus, Clostridioides difficile, and Candida) is insufficiently explained. While these are described as "clinically important taxa," the justification beyond C. difficile is unclear, as is the overall objective of these experiments within the context of the study.

Response: *The rationale for selection of these taxa for quantitative culture is based on different perspectives. 1) these taxa are mainly associated with human health and disease, according to a large body of literature in microbiome, 2) technical issues such as the ability to culture bacteria, along with the inherent issues of DNA extraction and sequencing - where relatively less abundant taxa may go undetected - the phenotypic pattern data provide an additional perspective on the taxa. 3) previous experience showed us these methods are complementary. Candida spp. and C. difficile are typical phenotypic indicators of antibiotic-induced dysbiosis. The loss of anaerobes is an undesirable condition for health, although not associated with a specific clinical condition. In the case of Enterococcus spp., this taxon may be less represented in microbiome sequencing platforms because of its inherent challenges of DNA extraction, i.e. that it can be resistant to lysis during DNA extraction and potentially less represented even with optimized bead beating methods[5].*

2.2. The relevance of these experiments would have been clearer if the taxa of interest were selected based on findings from the differential abundance analyses rather than a priori selection.

Response: *We agree that reviewing the differential abundant taxa data first to select taxa for the quantitative culture is a valid approach; however, we preferred to conduct the phenotypic quantitative culture before or in parallel with the 16S analysis for three reasons: 1) low abundant taxa in the sample can be missed by a primer and not selected for sequencing at the depth required, 2) Gram positive taxa including Enterococcus and Lactobacillus can be influenced by DNA extraction and representation in the reads can be affected, even if we use the bead beating method, 3) in previous experience (10.1016/S2666-5247(23)00360-9) - antimicrobial intervention study; we have experienced that these phenotypic methods are complementary to the 16S rRNA sequencing besides revealing the phenotypic resistance selection. Hence, independent of 16S sequencing, following the trajectory of viable bacterial count of the specific taxa, where possible, can complement the 16S-rRNA sequencing output*[6].

2.3. Regarding resistance analyses, the goal of exploratory testing in Enterobacteriales and Bacteroides is not clearly articulated. A broader screening across additional species or strains would have strengthened the conclusions.

Response: *The aim was to study potentially increased selection or emergence in the two-week treatment regimen. Macrolides, including azithromycin, have high resistance rates against both Enterobacterales and Bacteroides. As glasmacinal is a macrolide, these two taxa were considered good markers of increased selection or emergence. Furthermore, in an in vitro study, glasmacinal was shown to have negligible antimicrobial activity against 117 isolates from 17 bacterial species (these data are referenced in the introduction*[7]*). On the other hand, a two-week exposure to azithromycin was enough to induce emergence of azithromycin resistance in M. abscessus, which is a relatively slow growing microorganism compared with Enterobacterales and Bacteroides, which we used as indicators of aerobic and anaerobic taxa, respectively.*

We have included in the revised manuscript that Enterobacterales and Bacteroides were used as indicators of aerobic and anaerobic taxa, respectively (Methods Section).

2.4. Finally, it is unclear whether any technical replicates were included in the experiments. This should be clarified.

Response: *We agree with the reviewer that technical replicates can have value. The amount of sample was not sufficient for multiple replicates. Nevertheless, we analysed all the sample timepoints of a given study participant at the same time to minimize introducing measurement bias from media batch, interobserver, or dilutions, and other related factors.*

3. *Discussion section*

3.1. The first three paragraphs of the Discussion primarily summarize the results and would be more appropriate in the Results section. The authors could expand their

discussion with a more thorough comparison with existing studies on other macrolides and their effects on the gut microbiota.

Response: *We agree with the reviewer's comment regarding repetition of the results in the Discussion. We have limited the summary of the results to the first paragraph, and in subsequent paragraphs we have expanded our interpretation of the results. We have postulated reasons for the changes observed in particular taxa and compared with published data on antibiotic macrolides.*

3.2. In line 165, the authors report that Bifidobacterium longum decreases after azithromycin intake but increases following EP395 treatment. Speculation on possible mechanisms underlying this differential response would strengthen the discussion.

Response: *We propose that the increase in Bifidobacterium seen in this study might be due to targeted competitive exclusion [8,9]. The increase is potentially the result of more pronuced anti-inflammatory effects of glasmacinal (while not having antimicrobial effects), which might have not antagonized the anaerobes, and the Bifidobacterium taxa have benefited to outcompete (particularly the Clostridia sensu stricto-I which is the only taxa significantly depleted in the differentially abundant taxa). We have added our thoughts on the potential mechanism to the Discussion (lines 208-211).*

3.3. Finally, the authors conclude that EP395 treatment does not induce gut dysbiosis. This claim is not sufficiently substantiated. First, "dysbiosis" is not defined, and second, dysbiosis is not explicitly assessed in the analyses.

Response: *We agree that the term 'dysbiosis' has multiple interpretations[10] and requires defining in the context of our study. We have added the definition of dysbiosis used in our study to the introduction, including the distinction between therapeutic microbiome modulation and clinically meaningful dysbiosis (lines 67-78). Throughout the manuscript we have made it clearer that the aim of our study was to assess clinically meaningful dysbiosis (such as induced by antibiotics), rather than subtle ecological perturbations. The following text has been added to the Introduction:*

"The study aimed to evaluate loss of beneficial anaerobic taxa (Firmicutes and Bacteroidetes), expansion of pathobionts (such as Proteobacteria) and taxa typically associated with antimicrobial selection, and significant loss of microbial diversity, as a clinically meaningful dysbiosis following administration of glasmacinal".

We have updated the Conclusion section to ensure that our use of the term 'dysbiosis' is clear. The final sentence has been updated as follows:

"Overall, the trial showed that glasmacinal induced limited restructuring of the gut microbiome, without the defining hallmarks of antibiotic-like or clinically meaningful dysbiosis".

4. *General comments on sequencing data processing*

4.1. The authors employ a well-established pipeline for 16S rRNA gene sequencing analysis. However, important steps such as contaminant removal and 16S gene

copy-number correction do not appear to have been included. The authors should consider tools such as SCRuB for decontamination and RasperGade16S for copy-number correction.

***Response**: Our analytical pipeline follows established QIIME 2 best practices for Deblur-based 16S rRNA gene analysis of high-biomass human faecal samples. This included stringent sequence quality filtering (Phred q-score ≥30) and denoising using deblur denoise-16S, which incorporates positive filtering against a curated 16S reference database to remove non-target and low-quality sequences.*

Explicit post hoc contaminant decontamination methods (e.g., SCRuB) were not applied. Faecal samples typically yield strong biological signals with high microbial biomass, which substantially reduces susceptibility to reagent-derived contaminants compared to low-biomass sample types. Consistent with this, we did not observe features indicative of systematic contamination in quality control summaries or feature prevalence patterns. Tools such as SCRuB are particularly advantageous in low-biomass studies with dedicated negative controls, which was not the focus of the present work. We have clarified these considerations in the Methods (lines 464-477).

With respect to 16S rRNA gene copy-number correction, we considered this approach but did not apply it. Copy-number correction relies on reference genome availability and phylogenetic inference, which remain incomplete and uneven across taxa and are particularly unstable at amplicon sequence variant (ASV) resolution[11]. Moreover, empirical evaluations have shown that copy-number correction has limited impact on phylogenetically informed diversity metrics (such as Faith's PD and weighted UniFrac) and on differential abundance methods that explicitly model sampling depth and compositionality[12]. Given the within-subject longitudinal design and the focus on relative community structure rather than absolute abundance estimation, we therefore adhered to standard uncorrected relative abundance analyses, consistent with common practice in microbiome dysbiosis screening studies.

5. General comments on statistical analysis

5.1. Critical information regarding data filtering (e.g., removal of low-abundance or low-prevalence taxa) and data transformation is missing from both the Methods and Results sections. Supplementary Table 6 suggests that no filtering was applied, as 4,645 taxa were tested. Given the sparsity of microbiome data, this likely results in a high false-positive rate. In fact, q-values, which I interpret as multiple-testing–corrected p-values, appear mostly to be 0 or 1, and some differentially abundant features (e.g., g__Izemoplasmatales; s__uncultured_organism) are present in only 10 out of 63 samples. If this is the case, I would recommend applying a prevalence filter (e.g., removing taxa present in fewer than 30–40% of samples).

***Response**: We agree with the reviewer and have now applied prevalence filtering to the taxa abundance, retaining only taxa detected in at least 30% of samples at baseline and at least 30% of samples during treatment. Additionally, supplementary table 4 now includes only the differentially abundant taxa, providing a shorter and more readable list. This is now included in the methods section (lines 501-506)*

5.2. Given the longitudinal nature of the study, statistical methods should account for the non-independence of samples from the same participant. The methods described in the manuscript and GitHub repository suggest this may not have been done. The authors should clarify this point. If not accounted for, approaches such as paired tests, linear mixed-effects models, or block permutations for beta-diversity analyses should be considered.

Response*: We thank the reviewer for raising this important point. We agree with the reviewer that accounting for the non-independence of the samples would align better with the longitudinal design of the study. We have now updated the analyses of diversity metrics and differentially abundant taxa, as follows:*

We reanalyzed alpha diversity metrics (Faith's PD and Pielou's evenness) using linear mixed-effects models with timepoints (visits) as a fixed effect and subject ID as a random effect, thereby accounting for within-subject non-independence. Overall effects were evaluated using ANOVA on the mixed model, and pairwise visit comparisons were performed using estimated marginal means with FDR correction.

For beta-diversity, pairwise PERMANOVA analyses explicitly accounted for the longitudinal design by restricting permutations within subjects via the strata argument in adonis2. This approach preserves the paired structure of the study, ensuring that repeated measurements from the same participant do not inflate type I error, and allows robust assessment of temporal changes in microbial community composition.

For differential abundance analyses, we employed a mixed-effects compositional framework (LinDA) that explicitly models the longitudinal structure of the data while maintaining sensitivity in the context of a modest sample size[4]. These analyses were intended to contextualize and support our primary conclusion, that glasmacinal induces only mild and transient microbiome perturbations. In this setting, ANCOM-BC2, which is known to be highly conservative in small longitudinal cohorts, yielded uniformly null results across taxa. We interpret this outcome as reflecting limited statistical power rather than definitive evidence of no biological change, particularly given the concordant trends observed in diversity analyses and targeted culture-based assays. We have, therefore, focused on LinDA results and interpreted all differential abundance findings cautiously. The results of these analyses are now reported in the revised manuscript and provided as supplementary tables (Supplementary Table 1, 2, 3, and 4).

5.3. Could the authors clarify whether ANCOM-BC or ANCOM-BC2 was used, and whether relevant covariates (e.g., age, sex, intestinal transit time) were considered in the models?

Response: *We have updated the analysis, and the revised primary differential abundance analysis was performed using LinDA, a linear model-based approach with bias correction suitable for compositional microbiome data and repeated-measures designs (via subject random effects).*

Regarding covariates: The available clinical trial metadata for the microbiome sub-study shared with the authors of this study did not include age, sex, intestinal transit time, diet, BMI, or other potential confounders. Consequently, we were unable to adjust for these variables in the LinDA models. To reduce the risk of confounding, the parent trial enrolled a relatively homogeneous cohort of healthy adult volunteers. We acknowledge, however, that unmeasured or residual confounding by these or other factors

cannot be completely excluded. Still, the limited number of individuals in the trial would have complicated correction for the above-mentioned factors.

Minor comments

- The term "genotypic" is used where "in silico" would be more appropriate.

Response: *We have replaced 'genotypic' with '16S rRNA sequencing' throughout.*

- Line 142: The statement "loss of phylogenetic diversity without a significant shift in relative abundances of the remaining taxa" requires clarification. What is meant by "remaining taxa," and how can diversity decrease without affecting relative abundances?

Response: *The observed pattern indicates a selective loss of phylogenetically distinct lineages, as evidenced by the significant reduction in Faith's PD at both 1-week and 2-weeks post-treatment compared with Baseline-1 ($p < 0.05$), while community evenness (Pielou's evenness) remained unchanged across all visits. Faith's PD measures the total branch length of the phylogenetic tree spanned by the observed taxa, so a decrease reflects the loss or strong reduction of certain taxa that occupy distant or unique branches on the tree. The lack of significant change in evenness means that the relative abundance distribution among the taxa that persisted in the community did not undergo major reordering, no strong dominance by a few taxa emerged, and no large-scale blooms of opportunists occurred. In essence, some phylogenetically unique or distantly related lineages were selectively depleted, reducing overall phylogenetic diversity, while the proportional structure of the remaining core community members stayed relatively balanced.*

We have revised the results section where we present alpha and beta diversity metrics to address this point between lines 101-116 in the revised mnuscript.

- The claim that treatment "did not impact the essential anaerobic components" is unclear. How was this determined?

Response: *We observed significant changes limited to a few clades of lower taxonomic level within the anaerobic taxa, but the taxa belonging to the essentially anaerobic phyla (Firmicutes and Bacteroidetes), which comprise about 90% of gut microbes[13], are responsible for maintaining gut ecological balance and are related to health and disease[14,15], did not show a major community shift at higher taxonomic level (Figure 2 updated version). In contrast, significant and consistent reduction of Enterobacterales was observed at 1-week and 2-week timepoints. Although there were changes in Firmicutes, i.e. Clostridium sensu stricto-I, other anaerobic taxa were not significantly reduced. The essential anaerobic taxa here is referring to the large proportion of Firmicutes and Bacteroidetes that did not undergo significant changes. Moreover, the quantitative culture show that the viable count did not show change in Bacteroides and Lactobacillus, whereas Enterobacterales showed a similar trajectory as the 16S rRNA sequencing.*

- The authors state that "normalised counts were exported from the ANCOM-BC output for downstream visualisation." Please clarify how counts were normalized.

Response: *For downstream visualization only, normalized abundances were obtained by applying a centred log-ratio (CLR) transformation to the raw count table, after adding a small pseudo-count to handle zeros. CLR normalization was used to account for compositionality and differences in sequencing depth, and to enable meaningful comparison of relative changes across samples in an unconstrained Euclidean space.*

Importantly, CLR-transformed values were used exclusively for visualization and exploratory analyses (e.g., longitudinal abundance plots). All statistical inference for differential abundance was performed using LinDA on raw counts, which internally models library size and compositional effects and does not rely on CLR-transformed input. We have revised the Methods section to explicitly describe the CLR normalization procedure and to clarify its use for visualization only (Revised manuscript between lines 509-519).

- The terms "16S rDNA" and "metagenomic" are used inconsistently throughout the manuscript and should be replaced with "16S rRNA gene sequencing."

Response: *We have updated the manuscript throughout to use only the term '16S rRNA gene sequencing'.*

- In the "Summary of trial population and sample collection" section, additional cohort details would be helpful. A schematic figure summarizing the study design and sampling scheme is strongly recommended.

Response*: Thank you for this suggestion. We have added a new figure (Figure 1) to the manuscript, which is a schematic of the trial design and sample collection time-points.*

- The statement "The current literature on gut health focuses predominately on composition rather than diversity alone" should be reconsidered or supported with references, as numerous studies have examined microbiome diversity in relation to health and disease.

Response: *This text has been deleted. In response to the reviewer's suggestion to expand the discussion, we have implemented extensive changes in the Discussion section, and the above sentence was no longer needed.*

- Regarding the conclusion that "EP395 did not impose selection pressure over the 2 weeks of treatment," while two weeks may be insufficient to detect community-level changes, strain- or subspecies-level selection cannot be excluded based on the current analyses.

Response: *We agree that sub-species level changes were not studied and it cannot be excluded that changes might have occurred. However, we targeted Enterobacterales and Bacteroides as representative taxa for which azithromycin has antimicrobial activity, and to evaluate if the drug may have induced selection or cross resistance to macrolides (azithromycin used as class representative of macrolides).*

We believe the two-week duration can be sufficient to show substantial changes even if it cannot capture every species and strains level resistance selection; a previous study showed that Mycobacterium abscessus developed inducible resistance to azithromycin within two-weeks[16]. Enterobacterales and Bacteroides grow

faster than M. abscessus and potentially can develop selection resistance/inducible emergence of resistance in two weeks.

- The overview of QIIME 2 preprocessing is currently presented in the Results section and could be moved to the Methods section.

Response: *The text on QIIME 2 preprocessing has been moved to the Methods section*.

Reviewer #2 (Remarks to the Author):

Overall, the study is interesting and hypothesis generating, but not confirmatory or broad reaching. It is a very small study for making solid microbiome composition and diversity conclusions, and it represents a small subset of the population/treatment relevant to the hypothesis. There are some methodological questions that potentially impact the interpretation and conclusions. Some of the conclusions are over- or misstated and should be clarified.

Response: *We appreciate the reviewer's remarks. We have revised the manuscript in response to the comments, and consider it is significantly improved.*

1. First, why did the authors choose to conduct 16S sequencing? This seems rather inadequate in this day and age. An average of 135K reads per sample is a rather low sequencing depth and probably insufficient for meaningful differential features analyses. There is no discussion of how read depths are normalized across samples, despite there being up to 100% differences in read depths from the samples with the fewest to the highest reads. There's also no discussion of how rare ASVs vs zeros are handled. This is important, especially when conducting differential features analyses. The set of samples could have been analyzed by shallow or deep shotgun sequencing with minimal additonal cost. Overall, the microbiome methods are very average, somewhat outdated, and not described well (eg extraction methods, library prep, rarefaction...).

Response: *We have revised the Methods and Discussion accordingly and responded to the specific points below.*

Choice of 16S rRNA gene sequencing

We agree that shotgun metagenomic sequencing is often preferred in microbiome studies, particularly when the objective is to resolve strain-level variation, infer functional pathways, or interrogate resistomes. However, the aim of this study was more limited and explicitly ecological: to determine whether glasmacinal induces overt, antibiotic-like dysbiosis, characterized by broad reductions in diversity, major shifts in community structure, or expansion of taxa typically associated with antimicrobial selection.

For this objective, 16S rRNA gene sequencing (V3–V4 region) provides adequate taxonomic resolution to detect global community-level perturbations. Detecting antibiotic-like effects does not require strain-level resolution, and finer taxonomic discrimination would not materially alter the biological interpretation of the observed results. Importantly, functional consequences related to antimicrobial resistance were addressed independently using targeted culture-based assays (CFU quantification and azithromycin selection), which directly assess phenotypic resistance emergence and are more appropriate for evaluating functional selection in the absence of strong antibiotic pressure.

Sequencing depth

We acknowledge the reviewer's concern regarding sequencing depth and its implications for differential abundance testing. However, benchmark studies in the 16S rRNA gene amplicon literature indicate that broad trends in alpha and beta diversity and overall community structure can be reliably captured with moderate sequencing depths. For example, ecological patterns among samples were recapitulated with as few as ~2,000 reads per sample across diverse environments, and sequencing depths in the tens of thousands of reads generally capture stable diversity estimates and compositional differences[17,18]. Our average depth (~135,000 reads per sample) is substantially greater than these levels, supporting the robustness of the observed diversity and compositional changes for the questions addressed.

Benchmarking studies have consistently shown for 16S rRNA gene data that differential abundance performance is driven primarily by biological effect size, inter-individual variability, and statistical modelling choices, rather than sequencing depth, once moderate coverage is achieved[18]. The modest number of differentially abundant taxa identified reflects the subtle and heterogeneous nature of the microbiome response, rather than insufficient sequencing depth. Increasing sequencing depth alone would not be expected to overcome these biological and design-related constraints, a conclusion that is consistent with prior benchmarking studies. Accordingly, our interpretation focuses on the absence of overt antibiotic-like dysbiosis and emphasizes conservative inference rather than exhaustive detection of rare features.

Normalization, rarefaction, and handling of zeros

We agree that explicit reporting of normalization and zero handling is essential and have expanded the methods section accordingly: Diversity metrics were calculated following rarefaction implemented in QIIME2, with subsampling to 74,470 reads per sample. This depth retained all samples while minimizing library size bias for diversity estimation. Differential abundance testing was performed using LinDA, a mixed-effects, compositional framework that explicitly accounts for variable library sizes, repeated measures, and sparsity without requiring rarefaction. LinDA distinguishes between sampling zeros and potential structural (biological) zeros, which may reflect true absence under specific conditions rather than technical under sampling. To reduce noise from extremely sparse features, ASVs present in fewer than 30% of samples were excluded prior to modelling. This filtering step was chosen to balance sensitivity and interpretability and is now explicitly described in the 'Statistics and bioinformatic analyses' section of the Methods.

2. The size of the study is simply too small for the hypothesis tests and differential features analyses being conducted with the microbiome data. Too many studies have made bold conclusions on small sample sizes, with some conclusions flawed in such analysis and leading to significant medical conclusions that may not be well founded. In this case, the premise is that this study is sufficient to suggest that the novel macrolide can be useful for chronic treatment of COPD because no "dysbiosis" is caused, because the "core anaerobic taxa" remain intact, and because no resistance is generated. These are pretty bold statements that carry medical weight, but the study is not appropriately powered to make such bold statements with their implications.

Response: *We agree that small microbiome studies can be prone to overinterpretation, particularly when findings are extrapolated toward clinical use. For this reason, the scope of our conclusions has been*

carefully limited and further clarified in the revised manuscript. While we agree with the reviewer's comment for increased sample size, this study was designed as an exploratory objective within a DDI study to assess whether glasmacinal induces major changes in microbial compositional taxonomy. DDI studies typically enroll 12-20 study participants, this study enrolled 19 participants, included 17 in the microbiome study and 15 were able to attend all the visits to give faecal samples. The microbiome analysis was an exploratory objective within the trial and is intended as a preliminary assessment to inform microbial testing in longer-term trials of glasmacinal (as stated in the Discussion), not to inform medical decisions. The objective was to determine whether glasmacinal induces overt, antibiotic-like dysbiosis, defined as marked loss of diversity, major restructuring of community composition, collapse of core anaerobic taxa, or clear selection for resistance, effects that are typically detectable even in modestly sized, paired longitudinal cohorts following conventional macrolide exposure. The definition of dysbiosis used in the trial and the objectives of the research have been made clearer in the manuscript. Furthermore, because each study participant served as their own control, the within-subject correlation is sufficient to estimate changes in diversity metrics and to assess within-subject variability before and after introduction of glasmacinal. In the Discussion section, we have acknowledged the limitations of the study, and stated that the microbiome of COPD patients may differ substantially from the healthy subjects in the study, and consequently, the impact of glasmacinal may also differ. However, this trial represents the first clinical assessment of the potential impact of glasmacinal on the gut microbiota.

While larger cohorts are needed to detect subtle or heterogeneous microbiome changes, the current longitudinal design, incorporating dual pre-treatment baselines and paired statistical testing, is appropriate for identifying pronounced ecological perturbations. The absence of such signals therefore represents meaningful negative evidence with respect to antibiotic-like disruption, while explicitly not excluding smaller, individual-specific, or longer-term effects. We recognize that the inherent variability of the microbiome across individuals generally necessitates larger sample sizes with higher statistical power. However, longitudinal study designs inherently account for much of the inter-individual variability and noise, thereby improving the ability to detect true differences related to the intervention. In addition, determining a statistically powered sample size requires prior knowledge of the expected effect size, which typically comes from previous studies or pilot data. Because glasmacinal is a novel agent, no prior studies were available to inform effect size estimation. This study serves as a preliminary assessment to inform future, adequately powered studies, including those evaluating the impact of glasmacinal in COPD patients, as recommended in the manuscript.

3. With respect to diversity analyses, there was a difference in richness from before to after macrolide treatment. Baseline 1 and 1 week were significantly different compositions based on beta diversity analyses. Therefore, there are definitely differences as a result of macrolide administration, so the statement that "gut dysbiosis" was not induced becomes subjective as to what defines dysbiosis, and this is a central problem with the paper. Similarly, taken at face value despite the methodological questions, Figure 3 appears to show distinct differences from pre- to post-macrolide treatments, with several taxa often attributed to healthy functions (eg Clostridia, Prevotella, Akkermansia…), and there appears to be a distinct "overgrowth" of Bifidobacterium. These could all be characterized as "dysbiosis" from one subjective point of view.

Response: *We have tried to clarify better some microbiome changes that are more important and can therefore be considered dysbiosis (line 67-78). It is considered a sign of dysbiosis if Bifidobacterium decreases (reference 33), but in this case we are observing a brief transient increase. Thus, we do not see that the criteria for calling this a dysbiotic change are fulfilled and we think the overall previous conclusion still holds true.*

4. The paper also states that "core anaerobic taxa were preserved" which is subjective ("core") and not supported. The selective plating are elegant but do not include a general anaerobic agar (eg CDC blood agar…) or any agar for Clostridia other then C. difficile. Perhaps egg yolk agar after ethanol treatment or the like to assess whether spore-form Firmicutes are altered, which are a significant portion of the "core anaerobic taxa." The sequencing data do not assess overall Clostridia, and the discussion only mentioned Bacterodetes as core anaerobic taxa, which in fact many Clostidia genera are found in most healthy cohorts and associated with many "core, healthy" functions.

Response: *We agree with the reviewer's comment and suggestion in principle. In this study, we defined "core taxa" as the dominant anaerobic members of the gut microbiota (Firmicutes, Bacteroides), including Clostridia. In the log-normalized differential abundance analysis (Figure 4, the previous version, and Figure 2 of the updated version) remains somewhat subjective and lacks universal consensus. Following review of a large body of the literature in the field, we considered the dominant anaerobic genera such as Clostridia, Bacteroides, Bifidobacterium, and Lactobacillus to constitute the core microbiome. In this context, we did not observe major reductions in these other taxa, aside from* Clostridium sensu stricto-I *at the genus level, which may not represent the full diversity of clostridial species as shown in Figure 2, which showed the phylogenetic distance and relative abundance of ASVs. Nevertheless, this finding indicates a reduction following glasmacinal administration, and we cannot exclude the possibility of subtle changes in the microbiome can occur due to physiological and other factors unrelated to interventional drugs. Importantly, the objective of the study was to determine if glasmacinal exposure resulted in antimicrobial like changes in microbiome characterized by significant loss of the anaerobic taxa, increased abundance of pathobionts and increased selection/emergence of resistance to macrolides.*

We also acknowledge the reviewer's comment regarding the use of selective plating for Clostridia, *which could add value to the dataset. However, selective cultivation of clostridia presents several technical challenges when applied alongside other anaerobes (1) accurate enumeration often necessitates ethanol treatment to enrich for spores, which precludes the use of the same sample preparation for other anaerobes; and (2) such assays require dedicated clostridial quality control infrastructure. Together, these factors may introduce additional technical bias. Nonetheless, we agree that clostridial cultivation is valuable when conducted within an appropriate infrastructure and study design targeting specifically clostridia and spore forming subspecies. Although we did not perform colony counts for total anaerobes, we used Brucella blood agar as a quality control measure to confirm the integrity of the sample for anaerobic bacteria, ensuring that storage and processing conditions did not adversely affect anaerobe viability.*

5. The selective plating with antibiotics does suggest against emergence of phenotypic resistance to the antibiotics tested. However, it is only assessing the impact of a single treatment with the macrolide. It is possible that some phenotypic resistance could emerge after chronic use of the macrolide, which is a hypothesis set forth in the abstract. It is also noted that the selective plating method could miss the enrichment of antimicrobial resistance genes that could manifest as phenotypic resistance after more exposure to the macrolide. This is all acknowledged in the limitations section of the discussion, but I think it is a substantive limitation that precludes the scope of conclusions made in the article.

Response*: We agree with the reviewer that the study has limitations, as acknowledged in the Discussion. However, we consider the two-week duration can be sufficient to show substantial changes even if it cannot capture every species and strains level resistance selection; a previous study showed that Mycobacterium abscessus developed inducible resistance to azithromycin within two-weeks[16]. Enterobacterales and Bacteroides grow faster than M. abscessus and potentially can develop selection resistance/inducible emergence of resistance in two weeks. We agree that this study is a preliminary assessment and will be used to inform longer-term trials of glasmacinal with an optimized trial design. The conclusions have been carefully reconsidered to avoid over-interpretation.*

6. Overall, I suggest this is not acceptable for publication. If the sample size were more appropriate for the research questions, and if the methods were better described and applied, then a revision could address the challenges of imprecision with terminology ("dysbiosis", "core anaerobic taxa"). However, I do not think those correct the potential for conclusions and implications that outstrip the ability of the analyses.

Response: *We have significantly improved the manuscript in line with both reviewer's comments, including improvements in the statistical analysis, careful consideration of the terminology, and clear presentation of the study limitations and conclusions (to avoid overinterpretation). We believe that the revisions have substantially improved the manuscript, and that the findings will be of interest to the scientific community and the readership of Nature Communications. We thank the editor in advance for giving this manuscript consideration for publication.*

References:

1. *Committee for Medicinal Products for Human Use. ICH M12 Guideline on drug interaction studies. https://www.ema.europa.eu/en/ich-m12-drug-interaction-studies-scientific-guideline (2024).*
2. *U.S. Department of Health and Human Services. M12 Drug Interaction Studies Questions and Answers Guidance for Industry. https://www.fda.gov/media/180488/download/attachment (2024).*
3. *Kricker, J. A. et al. Nonantimicrobial actions of macrolides: Overview and perspectives for future development. Pharmacol. Rev.* ***73****, 233–262 (2021).*
4. *Zhou, H., He, K., Chen, J. & Zhang, X. LinDA: linear models for differential abundance analysis of microbiome compositional data. Genome Biol.* ***23****, (2022).*
5. *Hong, B. Y., Driscoll, M., Gratalo, D., Jarvie, T. & Weinstock, G. M. Improved DNA Extraction and Amplification Strategy for 16S rRNA Gene Amplicon-Based Microbiome Studies. Int. J. Mol. Sci.* ***25****, (2024).*
6. *Bellali, S. et al. Running after ghosts: are dead bacteria the dark matter of the human gut microbiota? Gut Microbes* ***13****, 1–12 (2021).*
7. *Gudjonsson, T. et al. A novel macrolide, EP395, with reduced antibacterial activity and an enhancing effect on respiratory epithelial barrier. Pulm. Pharmacol. Ther.* ***90****, (2025).*
8. *Soldavini Pelichotti, P. C. et al. Bifidobacteria antagonize the life cycle of Clostridioides difficile. Microb. Pathog.* ***199****, (2025).*
9. *Nakajima, A. et al. In vitro competition with Bifidobacterium strains impairs potentially pathogenic growth of Clostridium perfringens on 2'-fucosyllactose. Gut Microbes* ***17****, (2025).*
10. *Wei, S., Bahl, M. I., Baunwall, S. M. D., Hvas, C. L. & Licht, T. R. Determining Gut Microbial Dysbiosis: a Review of Applied Indexes for Assessment of Intestinal Microbiota Imbalances. Appl. Environ. Microbiol.* ***87****, 1–13 (2021).*
11. *Louca, S., Doebeli, M. & Parfrey, L. W. Correcting for 16S rRNA gene copy numbers in microbiome surveys remains an unsolved problem. Microbiome* ***6****, (2018).*
12. *Starke, R., Pylro, V. S. & Morais, D. K. 16S rRNA Gene Copy Number Normalization Does Not Provide More Reliable Conclusions in Metataxonomic Surveys. Microb. Ecol.* ***81****, 535–539 (2021).*
13. *Rinninella, E. et al. What is the healthy gut microbiota composition? A changing ecosystem across age, environment, diet, and diseases. Microorganisms* ***7****, (2019).*
14. *Magne, F. et al. The firmicutes/bacteroidetes ratio: A relevant marker of gut dysbiosis in obese patients? Nutrients vol. 12 Preprint at https://doi.org/10.3390/nu12051474 (2020).*

15. *Sommer, F., Anderson, J. M., Bharti, R., Raes, J. & Rosenstiel, P. The resilience of the intestinal microbiota influences health and disease. Nature Reviews Microbiology vol. 15 630–638 Preprint at https://doi.org/10.1038/nrmicro.2017.58 (2017).*

16. *Christianson, S. et al. Time-to-detection of inducible macrolide resistance in mycobacterium abscessus subspecies and its association with the erm(41) sequevar. PLoS One* ***11****, (2016).*

17. *Caporaso, J. G. et al. Global patterns of 16S rRNA diversity at a depth of millions of sequences per sample. Proc. Natl. Acad. Sci. U. S. A.* ***108****, 4516–4522 (2011).*

18. *Weiss, S. et al. Normalization and microbial differential abundance strategies depend upon data characteristics. Microbiome* ***5****, (2017).*

REVIEWER COMMENTS

Reviewer #1 (Remarks to the Author):

I would like to thank the authors for their efforts in revising the manuscript and for providing additional analyses. While the revisions have substantially improved the work, several concerns remain. In some instances, the authors provided purely textual responses to methodological queries—such as the decision to omit decontamination or copy-number correction—without data demonstrating that these steps would not have affected the results.

We appreciate the reviewers' careful reassessment of the revised manuscript and their recognition the improvements made. In response, we have further expanded the revision with additional data-driven analyses addressing the remaining concerns, with the full results provided in the supplementary material.

Response: *Deblur-based denoising and positive filtering were used as the contaminant-filtering step in the sequencing workflow. Deblur removes likely sequencing errors, chimeric sequences, and reads inconsistent with expected 16S rRNA amplicons through reference-guided filtering. Additional control-based decontamination methods such as SCRuB, microDecon, or SourceTracker require co-sequenced negative extraction or process controls, which were not part of the original study design and therefore cannot be applied retrospectively in a valid manner. In addition, given that the study analysed high-biomass faecal samples, low-level reagent contamination is unlikely to account for the observed community-level patterns.*

To address potential 16S rRNA gene copy-number bias, we have now performed a RasperGade16S sensitivity analysis and included the full results as a supplementary analysis (Supplementary Document 1). The corrected and original pipelines gave highly concordant results for alpha diversity, beta diversity, and differential abundance effect estimates. Importantly, copy-number correction did not reveal a broader microbiota perturbation; higher-rank taxonomic signals were generally similar or reduced. These results support the conclusion that glasmacinal did not induce broad antibiotic-like dysbiosis. A summary of this response has been added to the result section from line 145-156.

Furthermore, core limitations in the study design and the authors' subsequent justifications still constrain the ability to draw any robust conclusions, which ultimately impacts the relevance of the work. Specifically, the following points require further attention:

1. The claim that the sample size is "sufficient for a preliminary assessment" lacks support. The authors should provide a power analysis, perhaps using expected effect sizes from known antibiotic-induced dysbiosis, to justify the cohort size.

Response: *To further support the statement regarding sample size and analytical sensitivity, we performed an additional sensitivity analysis focused on the type of microbiome signal most relevant to this study: a large, consistent antibiotic-like perturbation of the gut microbiota. This analysis was added to clarify the magnitude of effects that the longitudinal paired glasmacinal*

design could detect, and to distinguish this from subtle, rare, transient, or highly heterogeneous microbiome changes that remain outside the scope of the study. The clinical trial was not designed as a microbiome efficacy study; rather, the microbiome analysis was included as an exploratory endpoint. For this question, the longitudinal paired design is important: each participant contributed repeated samples over time and served as their own baseline control, improving sensitivity by reducing the influence of between-participant variability compared with a cross-sectional comparison of unrelated individuals.

To address the sample-size concern quantitatively, the supplementary report (Supplementary Document 2) evaluates the empirical sensitivity of the glasmacinal microbiome analysis using three complementary approaches. First, it compares baseline-to-baseline variation with on-treatment changes in alpha diversity. The paired analysis detected clear within-subject reductions in Faith's phylogenetic diversity and observed richness during treatment, with $Cohen's\ d_z$ *values of approximately -1.4 to -1.7, whereas baseline-to-baseline drift was negligible. This demonstrates that the analysis was not globally insensitive: it could distinguish a consistent treatment-period signal from background within-subject variation. Second, these effect sizes are placed in the context of external antibiotic benchmark datasets, including azithromycin, erythromycin, amoxicillin-clavulanate, tebipenem, and a meropenem/gentamicin/vancomycin combination. These datasets are not used as direct drug-to-drug comparisons, but as benchmarks for the magnitude and pattern of antibiotic-associated dysbiosis signals detectable in paired microbiome analyses. Third, the report evaluates beta-diversity and genus-level sensitivity. The Bray-Curtis/PERMANOVA analysis detected a small but significant community shift after glasmacinal, and the LinDA-based genus-level sensitivity analysis showed that sizeable and consistent genus-level changes, particularly in abundant genera, could be detected.*

As we mentioned in the main text, the study may not be powered to detect subtle, rare, transient, or highly heterogeneous changes. However, the paired longitudinal framework showed adequate empirical sensitivity to detect large, consistent within-subject microbiome perturbations relevant to the main question: whether glasmacinal induced a broad antibiotic-like dysbiosis signal at the sampled treatment timepoints. The manuscript has been updated to include this information in both the Results section (lines 156-168) and the trial design section (lines 419-431).

2. The discrepancy between ANCOM-BC and LinDA results raises concerns. Rather than selecting one method for the main text, the authors should transparently present both, highlighting areas of agreement and disagreement (e.g., directionality). If ANCOM-BC2 is indeed more conservative, this may actually be a methodological strength given the small sample size.

Response: *The initial analysis used ANCOM-BC (v1) as implemented in QIIME 2, which does not support random-effects terms and cannot account for within-subject correlation across repeated visits. In our design, each participant contributed samples across four timepoints and gut microbiome profiles are strongly subject-specific. An analysis that omits a subject-level random effect tests cross-sectional differences between visit groups rather than within-individual treatment-associated change, a fundamental misspecification for this longitudinal design. To*

assess whether this methodological distinction materially affects biological conclusions, we conducted a post hoc comparison of ANCOM-BC and LinDA across all 13,932 ASV × visit combinations (Table RS1 below). The two methods showed high concordance in fold-change magnitude (Pearson r = 0.965, Spearman ρ = 0.945) and perfect directional agreement (0 of 172 significant ASVs showed opposite directions of effect). Disagreement was confined to significance thresholds, with LinDA detecting 77 ASVs that ANCOM-BC missed and ANCOM-BC flagging 37 that LinDA did not, a pattern consistent with ANCOM-BC's reduced power under the mis specified cross-sectional framework. We do not consider this a biological discrepancy; it is the expected statistical consequence of replacing a model that cannot accommodate the repeated-measures design with one that can. Given that the study objective is to characterize within-individual microbiome responses to glasmacinal exposure, a fundamentally longitudinal question, we do not consider cross-sectional comparisons between visit groups as methodologically appropriate or biologically informative. We therefore retain LinDA as the sole primary differential abundance framework and do not present ANCOM-BC results in the manuscript. The high fold-change concordance (r = 0.965) and perfect directionality agreement (0 discordances) provide strong evidence that the biological conclusions are robust across analytical approaches, while the choice of LinDA ensures the statistical model is correctly aligned with the study design.

Moreover, we agree that conservative methods can be valuable in small studies because they reduce false-positive findings. ANCOM-BC2 (v2), while supporting random-effects structures, applies a level of conservatism that rendered it uninformative at this sample size, producing no detectable signal despite clear and concordant evidence of biological activity in alpha diversity, beta diversity, and culture-based endpoints. A method that cannot detect signal under conditions where independent analytical domains consistently indicate biological activity does not serve as a meaningful analytical framework for this dataset.

Ultimately, LinDA was selected because it directly models the repeated-measures structure through a mixed-effects framework, has been validated for FDR control in compositional microbiome data with repeated measures, and produces results consistent with the independent lines of evidence across this study. We do not consider the choice of LinDA as selective reporting, it is the method most aligned with the study design and the one whose output can be meaningfully interpreted in the context of the broader dataset.

***Table RS1**: Comparison of LinDA vs ANCOM-BC Differential Abundance Analysis. Significance is defined as p.adj < 0.05 and |LFC|>1*

Metric	***Value***	***Interpretation***
Total ASV × timepoint comparisons	*13,932**	*Comprehensive assessment across all features*
Significance Agreement		
Both significant (LinDA & ANCOM-BC)	*58*	*concordant hits*
LinDA only (ANCOM-BC misses)	*77*	*Lower power in ANCOM-BC (lacks repeated-measures modelling)*
ANCOM-BC only (LinDA misses)	*37*	*False positives from independent-observations assumption*
Neither significant	*13,760*	*~99% of all comparisons (expected)*
Fold-Change Correlation		
Pearson r	*0.965*	*Near-perfect agreement on magnitude*
Spearman ρ	*0.945*	*Near-perfect agreement on ranking*
Directional Agreement (among 172 significant taxa)		
Concordant (same direction)	*172 / 172*	*100% — every significant ASV agrees in direction*
Discordant (opposite direction)	*0 / 172*	*Zero sign reversals across both methods*

* *Total unfiltered ASVs detected (n = 4,644). Each ASV was assessed separately in three pairwise comparisons (BL2, 1W, and 2W vs BL1) by both ANCOM-BC and LinDA, resulting in repeated statistical assessments of the same ASV across timepoints.*

3. Several interpretations of differential abundance appear to rely on visual inspection rather than formal testing. For instance, the claim that drug effects are distributed across the taxonomic tree should be supported by an enrichment analysis. Additionally, taxa trajectories should include individual data points rather than just group-level visualizations.

Response: *To address this point, we added a permutation-based phylogenetic dispersion analysis. All the ASVs present as tips in the phylogenetic tree were used as the background set, and FDR-significant ASVs matching tree tips were used as the foreground set. This analysis tested whether significant ASVs had a lower mean pairwise phylogenetic distance than expected by random sampling from the tree-tip background. The analysis showed no significant phylogenetic clustering of significant ASVs after 9,999 permutations (97 significant ASVs among 1,180 tree tips; observed MPD = 0.540, null mean MPD = 0.598; standardized effect size (SES) = −1.66; one-sided permutation p-value for clustering = 0.053). We have therefore revised the text to state that significant ASVs were not significantly clustered within the ASV phylogeny, rather than relying on visual inspection alone (Lines 126-128).*

Moreover, Figure 4 has been revised to display individual subject trajectories (coloured lines) alongside the population-level median (black line), to provide a more transparent view of both individual variation and group-level trends.

4. In the experimental section, the rationale for the selected bacterial taxa remains weak. The claim that they are "associated with human health" is overly broad and applies to many omitted taxa (e.g., F. prausnitzii or Bifidobacterium). Furthermore, citing "technical issues in culturing" as a selection criterion is only valid if it refers to specific taxa identified in the in-silico analysis. It remains unclear what the specific added value of this targeted screening is in its current form.

Response: *The method section is now updated with the following text as justification of the quantitative culture: "In quantitative culture, besides evaluation of phenotypic resistance emergence as complementary method to the 16S rDNA approach, Bacteroides, Enterobacterales, Enterococcus, and Lactobacillus were selected as strategic sentinel groups because they collectively capture the key viable ecological and clinical dimensions of macrolide-induced perturbation in the gut microbiome. Bacteroides represent core community stability and metabolic capacity[1,2]; Enterobacterales as sensitive indicators of dysbiosis and opportunistic expansion[3]; Enterococcus as a robust reservoir for antimicrobial resistance[4] and most likely to be missed by 16S rDNA due to inherent limitations of the DNA extractions[5] was found important to complement with selective quantitative culture; and Lactobacillus reflects barrier integrity and beneficial microbial function[6,7] besides the fact that it is one of difficult to lyse bacteria that we need to complement with phenotypic approach[8].*

Focusing on these groups during macrolide intervention is therefore strategic, as they constitute the primary targets of antibiotic pressure, the main reservoirs of transferable resistance, and the most informative markers of both disruption and recovery. Quantitative culture is specifically employed because it provides absolute counts of viable organisms and enables direct phenotypic assessment, including antibiotic susceptibility that sequencing-based approaches alone cannot fully cover. While taxa such as Faecalibacterium and Bifidobacterium are important indicators of gut health, they were not prioritized here due to methodological and interpretive limitations in a culture-based approach. Faecalibacterium is an extremely oxygen-sensitive anaerobe that is difficult to reliably recover and quantify using standard culture techniques[9], making results less reproducible and less suitable for phenotypic assays. Bifidobacterium, although more amenable to culture, is highly strain-diverse and less directly implicated in macrolide resistance changes or horizontal gene transfer compared to the selected taxa[10,11]. Moreover, Faecalibacterium and Bifidobacterium are better captured through sequencing-based approaches for relative abundance and diversity profiling. The targeted taxa provide a stronger link between cultivability, resistance surveillance, and clinically implementable outcomes in the context of antibiotic intervention. The objective of adding the quantitively culture was not aimed as confirmatory to the in silico, rather as strategic approach to complement or supplement the in-silico by selecting a likely representative and predominant common taxa relevant in antibiotic related changes from both Gram-positive and Gram-negative[8,12]." The summary of this information has been added to the methods section of the manuscript from lines 450-464.

Reviewer #2 (Remarks to the Author):

1. Overall, I appreciate the changes made to the manuscript, which are primarily textual rather than analytical. I still feel the cohort is small and hypothesis generating, rather than conclusive, yet the text still appears aimed at conclusions. A few detailed points.

   Response: *While the cohort is of moderate size, the strength of the study lies in the temporal monitoring over time with many sampling time points, and moreover the post hoc sample size analysis supports that the size is adequate to capture typical changes observed with antibiotics including antibiotic macrolides (Supplementary Document 2).*

2. I still view the statement that glasmacinal induced changes "…without clinically meaningful dysbiosis" is an overstated of conclusions. There are changes, they appear relatively modest, but there is no assessment of clinical meaningfulness for those changes.

   Response: *We have updated the text mentioned above to 'without the hallmarks of antibiotic-like dysbiosis' and removed the text 'without clinically meaningful dysbiosis'.*

3. Lines 142-145: authors state findings that the "treatment-associated microbiome shift was not driven by a single dominant taxon but instead reflected coordinated increases and decreases among taxa with distinct ecological roles" but they do not discuss or address ecological roles of the taxa that changed via either functional genomics herein or literature assessment of the changing taxa. Therefore, I still find this conclusion to be somewhat overstated.

   Response*: This text is now removed and replaced with a new paragraph, which also addresses Reviewer 1's comments and suggestions. Updated in the main text from lines 145-168.*

4. While not comprehensive, the phenotypic culturing is probably a strength of this manuscript. The data rather clearly indicate that several taxa do not change (Lactobacillus, Bacteroides…whereas Enterobacterales clearly is decreased by glasmacinal. The notable methodological weakness in this area is the lack of definitive characterization of isolated and quantified colonies, even by gram stain and microscopic morphology. This is a rather basic technique that would provide strength to the conclusions by providing greater confidence that the measured colonies represent the taxa they are claimed to. This is not a concern with the chromagar plates due to their demonstrated selectivity and confirmatory chromagenic observation. However, BKV and Rogosa agar are well known to have breakthrough of non-Bacteroidetes and non-Lactobacilli, respectively.

   Response: *We agree with this view – it is possible that non-Bacteroides may grow on BKV, and non-Lactobacillus species may grow on the Rogosa agar. We employed a conservative approach when we counted the colony by focusing on the typical colony morphologies and where necessary by using MALDI-TOF to differentiate the colonies. Moreover, we have made the Rogosa agar strictly at pH 5.5 which significantly reduces the load of other non-Lactobacillus species. We did not choose to use Gram-staining as this would generally be difficult to account for every colony possible on the plate. In fact, we identified few non-lactobacilli small colonies found to be enterococci and pediococci; these colonies were small, checked with MALDI-TOF, and not counted.*

Moreover, they were presented in just a few colonies per plate. Similarly, in very few cases, Prevotella species were encountered on BKV and disregarded from counting. But in an attempt to differentiate the different colony morphologies on BKV, we identified different species of Bacteroides from same plate, and all were counted together as Bacteroides. The text has been updated in the method section of the manuscript from lines 479-486.

5. My opinion is that the conclusions are still overstated compared to the strength of the analysis. For example "glasmacinal induced limited ecological perturbations without the defining hallmarks of antibiotic-like or clinically concerning dysbiosis." These appear aimed at making strong clinically relevant conclusions that just aren't supported the analyses.

   Response: *This conclusion implies only to this cohort, which is a healthy adult population. We clearly note that a study is needed to determine whether this conclusion is valid for COPD patients. We recommended a more robust study which includes COPD patients, as the microbiome of a healthy population may not represent the microbiome of COPD patients and that there is a clear age difference between the current study subjects compared with COPD patients (average age approximately 65 years). Thus, we feel that we have qualified our statements and acknowledged the limitations.*

6. Line 200 discussion of Akkermansia reduction being due to immunomodulatory properties should be elaborated. Are there references? This seems to be based on the conclusion that glasmacinal has no antimicrobial properties, but the dramatic decreases in phenotypic recovery of Enterobacterales suggests otherwise. It may be that glasmacinal has MIC > 64 ug/mL in vitro but that the concentrations exceed this in the colon such that antimicrobial activity can be observed. There are very clear precedents for this, such as oral vancomycin inhibition of Bacterodia in C. difficile patients despite in vitro MICs being very high. All of this is to suggest that a claim of an immunomodulatory effect on Akkermansia should be better supported.

   Response: *Macrolides, including glasmacinal, have been shown to reduce mucus. Unlike most anaerobes, mucin is the main carbon source for Akkermansia in the gut, so a reduction in mucus could potentially lead to a decrease in the abundance of this genus, but not other anaerobes. We have added this information to the manuscript (225-228) with applicable references. Vancomycin when given orally has negligible oral systemic absorption and is concentrated in the gut. For systemic infections it must be given intravenously. Glasmacinal is rapidly absorbed when given by oral administration (e.g. Singh et al 2026, primary publication for this work). Thus, the pharmacokinetics of glasmacinal do not suggest it will concentrate in the gut.*

7. Similarly, I think the statement "…anti-inflammatory and immunomodulatory effects of glasmacinal might have promoted stability of anaerobic taxa…" is quite overstated. It would be better to just state the facts--glasmacinal did not appear to dramatically disrupt or reduce anaerobic taxa in this limited cohort. If there are proposed anti-inflammatory or immunomodulatory contributions to this, clearly state that these are speculations based on specific literature citations

   Response: *This section has been updated with the following text, "Even though the anti-inflammatory and immunomodulatory effects of glasmacinal did not significantly disrupt anaerobic taxa, ……"*

8. Line 241, the statement "…the 2-week treatment period in this trial was likely sufficient for the detection of resistance" is a vast speculation. The invocation of M. abscessis resistance development does not teach about Enterobacterales resistance potential. These are rather distance cousins whose propensity to develop resistance may be quite divergent and highly dependent on how critical is the pathway wherein resistance develops. Perhaps in vitro assessment of the potential to evolve resistance vs number of passages would be supportive of such a statement, but there is no data to support that here.

***Response**: We agree that this statement is somewhat speculative and that detection of resistance can even vary between strains of the same species. We have added a non-mycobacterial example to the relevant section of the discussion and softened the comparison and conclusions. This section of the discussion from line 266-275 is now updated as follows: "In one previous study, treatment of Mycobacterium abscessus infections for 2 weeks with azithromycin induced azithromycin resistance. Another study conducted to evaluate the lag time in pharyngeal carriage of macrolide resistance after azithromycin and clarithromycin administration showed that the peak resistance was seen at day 8 in streptococci.[14] Considering that Enterobacterales and* Bacteroides *exhibit faster growth rates (24–48 hours) than mycobacterial species and probably comparable growth rates to streptococci, the 2-week treatment period in this trial was likely sufficient for the detection of at least the earliest resistance phenotype. However, it should be noted that the mechanisms of resistance emergence in Enterobacterales are most likely different from* M. abscessus *and streptococci. As glasmacinal is intended for long-term treatment, microbial testing for emergence resistance in longer-term trials will be important."*

References:

1. Wexler, H. M. Bacteroides: The good, the bad, and the nitty-gritty. *Clinical Microbiology Reviews* vol. 20 593–621 Preprint at https://doi.org/10.1128/CMR.00008-07 (2007).
2. Zafar, H. & Saier, M. H. Gut Bacteroides species in health and disease. *Gut Microbes* vol. 13 1–20 Preprint at https://doi.org/10.1080/19490976.2020.1848158 (2021).
3. Moreira de Gouveia, M. I., Bernalier-Donadille, A. & Jubelin, G. Enterobacteriaceae in the Human Gut: Dynamics and Ecological Roles in Health and Disease. *Biology* vol. 13 Preprint at https://doi.org/10.3390/biology13030142 (2024).
4. Dubin, K. & Pamer, E. G. Enterococci and Their Interactions with the Intestinal Microbiome. *Microbiol. Spectr.* **5**, (2017).
5. Toto, F., Scanu, M., Gramegna, M., Putignani, L. & Del Chierico, F. Impact of DNA Extraction and 16S rRNA Gene Amplification Strategy on Microbiota Profiling of Faecal Samples. *Int. J. Mol. Sci.* **26**, (2025).
6. Rashid, M. U. *et al.* Determining the long-term effect of antibiotic administration on the human normal intestinal microbiota using culture and pyrosequencing methods. *Clinical Infectious Diseases* **60**, S77–S84 (2015).

7. Yu, Q., Yuan, L., Deng, J. & Yang, Q. Lactobacillus protects the integrity of intestinal epithelial barrier damaged by pathogenic bacteria. *Front. Cell. Infect. Microbiol.* **5**, (2015).

8. Zhang, B. *et al.* Impact of Bead-Beating Intensity on the Genus- and Species-Level Characterization of the Gut Microbiome Using Amplicon and Complete 16S rRNA Gene Sequencing. *Front. Cell. Infect. Microbiol.* **11**, (2021).

9. Khan, M. T. *et al.* Synergy and oxygen adaptation for development of next-generation probiotics. *Nature* **620**, 381–385 (2023).

10. Terlizzi, M., Speranza, B., Sinigaglia, M., Corbo, M. R. & Bevilacqua, A. Antibiotic Resistance in Bifidobacterium animalis subsp. lactis and Bifidobacterium longum: Definition of Sensitivity/Resistance Profiles at the Species Level. *Microorganisms* **13**, (2025).

11. Moubareck, C., Gavini, F., Vaugien, L., Butel, M. J. & Doucet-Populaire, F. Antimicrobial susceptibility of bifidobacteria. *Journal of Antimicrobial Chemotherapy* **55**, 38–44 (2005).

12. Rinninella, E. *et al.* What is the healthy gut microbiota composition? A changing ecosystem across age, environment, diet, and diseases. *Microorganisms* **7**, (2019).

13. Kricker, J. A. *et al.* Nonantimicrobial actions of macrolides: Overview and perspectives for future development. *Pharmacol. Rev.* **73**, 233–262 (2021).

14. Malhotra-Kumar, S., Lammens, C., Coenen, S., Herck, K. Van & Goossens, H. *Articles Eff Ect of Azithromycin and Clarithromycin Therapy on Pharyngeal Carriage of Macrolide-Resistant Streptococci in Healthy Volunteers: A Randomised, Double-Blind, Placebo-Controlled Study*. *www.thelancet.com* vol. 369 www.thelancet.com (2007).

Pointy by point response

Reviewer #1 (Remarks to the Author):

I would like to thank the authors for their thorough revisions. They have successfully addressed the majority of my concerns, provided helpful supplementary analyses, and clarified several methodological choices. I still believe it is important to present and discuss the results using ANCOM-BC2. As previously noted—and acknowledged by the authors—a comparison with ANCOM-BC (v1) is not informative here, given that the method does not properly handle repeated measurements.

> **Response:** *ANCOM-BC2 had already been applied during the analysis, but it identified very few differentially abundant features, particularly at ASV level, and therefore did not provide sufficient resolution to serve as the principal framework for describing the taxonomic response. As requested, we have now included the complete ANCOM-BC2 results in Supplementary Table 4 and a direct comparison with LinDA in Supplementary Table 6.*
>
> *The inclusion of ANCOM-BC2 did not reveal a separate or contradictory biological pattern. Rather, ANCOM-BC2 identified a much smaller subset of the associations detected by LinDA, and all findings shared between the two methods showed the same direction of effect. The difference was therefore primarily one of detection breadth. In this dataset, ASV-level sparsity, the multiple-testing burden, variance regularization, and the pseudo-count sensitivity requirement substantially limited the number of associations retained by ANCOM-BC2. LinDA was therefore used to characterize the broader distributed response, whereas ANCOM-BC2 identified a more restricted cross-method subset. Had ANCOM-BC2 been used as the sole differential-abundance framework, its highly restricted output would have made it difficult to distinguish a genuinely limited biological response from limited statistical sensitivity in this dataset. Presenting both methods allows the broader taxonomic response to be described while also identifying the associations that are robust across analytical frameworks.*
>
> *More generally, our analytical choices were intended to avoid underestimating treatment-associated microbiome changes. For alpha diversity, we emphasized Faith's phylogenetic diversity because it captures phylogenetic richness and showed a clearer treatment-associated reduction than Shannon diversity. For beta diversity, weighted UniFrac was selected because it incorporates both abundance and phylogenetic relatedness and was more sensitive to the observed community displacement than non-phylogenetic measures such as Bray-Curtis or Jaccard. Similarly, LinDA was retained for differential abundance because it provided greater resolution of the longitudinal taxonomic response.*
>
> *Although a large panel of diversity metrics and differential-abundance methods could be presented, doing so would substantially expand the manuscript without necessarily improving biological interpretation. Some measures are also less sensitive, by design, to the type of change observed here. For example, Shannon diversity does not incorporate phylogenetic relatedness, while Bray-Curtis and Jaccard do not account for phylogenetic distances between taxa.*
>
> *Importantly, our conclusion of a transient, moderate restructuring of the microbiome was not based on selecting analyses that minimized the observed changes.*
